# High-density linkage mapping in a pine tree reveals a genomic region associated with inbreeding depression and provides clues to the extent and distribution of meiotic recombination

**DOI:** 10.1186/1741-7007-11-50

**Published:** 2013-04-18

**Authors:** Emilie Chancerel, Jean-Baptiste Lamy, Isabelle Lesur, Céline Noirot, Christophe Klopp, François Ehrenmann, Christophe Boury, Grégoire Le Provost, Philippe Label, Céline Lalanne, Valérie Léger, Franck Salin, Jean-Marc Gion, Christophe Plomion

**Affiliations:** 1 INRA, UMR1202 BIOGECO, F-33610 Cestas, France; 2 Université de Bordeaux, UMR1202 BIOGECO, F-33170 Talence, France; 3 HelixVenture, F-33700, Mérignac, France; 4 Plateforme bioinformatique Toulouse Midi-Pyrénées, UBIA, INRA, F-31326 Auzeville Castanet-Tolosan, France; 5 INRA, UR0588 Amélioration Génétique et Physiologie Forestières, F-45075 Orléans, France; 6 INRA, UMR547 PIAF, Les Cézeaux, 24 Avenue des Landais, F-63177 Aubière cedex, France; 7 CIRAD, UMR AGAP, Campus de Baillarguet TA 10C, F-34398 Montpellier Cedex 5, France

**Keywords:** Unigene, SNP array, Linkage mapping, Segregation distortion, Recombination, Maritime pine, *Pinus pinaster*

## Abstract

**Background:**

The availability of a large expressed sequence tags (EST) resource and recent advances in high-throughput genotyping technology have made it possible to develop highly multiplexed SNP arrays for multi-objective genetic applications, including the construction of meiotic maps. Such approaches are particularly useful in species with a large genome size, precluding the use of whole-genome shotgun assembly with current technologies.

**Results:**

In this study, a 12 k-SNP genotyping array was developed for maritime pine from an extensive EST resource assembled into a unigene set. The offspring of three-generation outbred and inbred mapping pedigrees were then genotyped. The inbred pedigree consisted of a classical F2 population resulting from the selfing of a single inter-provenance (Landes x Corsica) hybrid tree, whereas the outbred pedigree (G2) resulted from a controlled cross of two intra-provenance (Landes x Landes) hybrid trees. This resulted in the generation of three linkage maps based on SNP markers: one from the parental genotype of the F2 population (1,131 markers in 1,708 centimorgan (cM)), and one for each parent of the G2 population (1,015 and 1,110 markers in 1,447 and 1,425 cM for the female and male parents, respectively). A comparison of segregation patterns in the progeny obtained from the two types of mating (inbreeding and outbreeding) led to the identification of a chromosomal region carrying an embryo viability locus with a semi-lethal allele. Following selfing and segregation, zygote mortality resulted in a deficit of Corsican homozygous genotypes in the F2 population. This dataset was also used to study the extent and distribution of meiotic recombination along the length of the chromosomes and the effect of sex and/or genetic background on recombination. The genetic background of trees in which meiotic recombination occurred was found to have a significant effect on the frequency of recombination. Furthermore, only a small proportion of the recombination hot- and cold-spots were common to all three genotypes, suggesting that the spatial pattern of recombination was genetically variable.

**Conclusion:**

This study led to the development of classical genomic tools for this ecologically and economically important species. It also identified a chromosomal region bearing a semi-lethal recessive allele and demonstrated the genetic variability of recombination rate over the genome.

## Background

Maritime pine (*Pinus pinaster* Ait.) is a diploid species with 24 chromosomes (2n = 2x = 24). It plays an important ecological and economic role in southwestern Europe, where over four million hectares (ha) are covered by planted and natural forests of this species. Its wood has various end uses (lumber, pulp and paper, particleboard, resin) and several breeding programs have been developed in France, Portugal and Spain, to improve wood productivity and quality, and resistance to biotic and abiotic stresses (reviewed by Mullin *et al*. [1]).

Like other gymnosperms, it has a large genome size due to retrotransposon expansion [2]. This genome, amounting to 24 Gb/C [3] is about 200 times larger than that of the model plant *Arabidopsis thaliana*. Despite this very large difference between the chromosomes of any conifer and *Arabidopsis*, genetic mapping studies in pines and spruces have clearly demonstrated that the number of crossing-over events per chromosome is highly conserved across the plant kingdom, with two to four chiasmata per bivalent (1 chiasma = 50 centimorgans (cM), [4]) regardless of the physical size and fraction of coding DNA. The large genomes of conifers have greatly hindered their sequencing (but see [5]), but they have also prompted large-scale investigations of expressed gene sequences for the inference of putative unigene sets (reviewed by McKay and Dean [6]) and initiatives to map these genes (reviewed by Ritland *et al*. [7]). Indeed, advances in high-throughput genotyping technology have led to the establishment of dense gene-based maps for spruces [8] and pines [9]. It is anticipated (as illustrated here) that conifer genetic mapping activities will continue to grow and flourish to study the genetic architecture of quantitative traits and facilitate the future assembly of the genome sequences of these species. These next-generation linkage maps are being established with single-nucleotide polymorphism (SNP) markers, the rapid discovery of which is being facilitated by massively parallel sequencing, which also provides information about their abundance in transcribed regions. Furthermore, the availability of mature high-throughput genotyping technologies is making possible the multiplex analysis of thousands of SNPs at relatively low cost [10,11].

Following the validation of SNPs on the basis of their Mendelian segregation in mapping pedigrees, SNP-arrays are now a tool of choice for population and conservation genomics (for example, [12]) and for genomic selection (for example, [13]). In addition to the various downstream genetic applications of linkage mapping, meiotic maps also offer more fundamental opportunities, such as: i) understanding genome evolution, as neatly illustrated by Pavy *et al*. [8] for the evolutionary history of gene duplication and the extent of macrosynteny across conifer genera; ii) studies of the environmental and biological factors (sex, genetic background) affecting meiotic recombination [14] and analysis of the distribution of crossover events on chromosomes [15,16]. Indeed, meiotic recombination events are not randomly distributed in the genome, but instead occur in specific regions called recombination hotspots [17,18], and recombination is known to have a major impact on mutation and selection [19]; and iii) the identification of loci displaying a departure from Mendelian expectations (segregation distortion), indicating that selection has occurred during one or several phases of the plant’s life cycle [20]. In this context, comparative analyses of segregation distortion between inbred and outbred genotypes is of particular interest, as this approach could be used to detect genomic regions bearing loci with lethal or semi-lethal alleles, which are believed to be abundant in conifers (reviewed by Williams [21]).

We had four objectives in this study: i) to establish a gene catalog (unigene set) from the assembly of expressed sequenced tags (ESTs) generated mostly with the Roche’ 454 sequencing platform; ii) to design a custom SNP-array by *in silico* mining for single-nucleotide and insertion/deletion polymorphisms; iii) to validate the SNP assay by genotyping two mapping populations with different mating types (inbred versus outbred), and different genetic compositions of the parental genotypes (intraprovenance versus interprovenance hybrids); and iv) to generate and compare linkage maps, for the identification of chromosomal regions associated with deleterious mutations, and to determine whether the extent of meiotic recombination and its distribution along the length of the chromosomes are affected by sex or genetic background. The genomic resources described in this study (unigene set, SNP-array, gene-based linkage maps) have been made publicly available. They constitute a robust platform for future comparative mapping in conifers and modern approaches aimed at improving the breeding of maritime pine.

## Results

### Description of the maritime pine unigene set

We obtained 2,017,226 high-quality sequences, 1,892,684 of which belonged to the 73,883 multisequence clusters (or contigs) identified, the remaining 124,542 ESTs corresponding to singletons. This created a gene index of 198,425 different sequences, assuming that the singleton ESTs corresponded to unique transcripts. The number of unique sequences is almost certainly overestimated, because some sequences probably arise from non-overlapping regions of the same cDNA or correspond to alternative transcripts. The assembly was denoted PineContig_v2 and is available from [22].

### SNP-assay genotyping statistics

We used the maritime pine unigene set to develop a 12 k SNP array for use in genetic linkage mapping. The mean call rate (percentage of valid genotype calls) was 91% and 94% for the G2 and F2 mapping populations, respectively.

**Figure 1:**
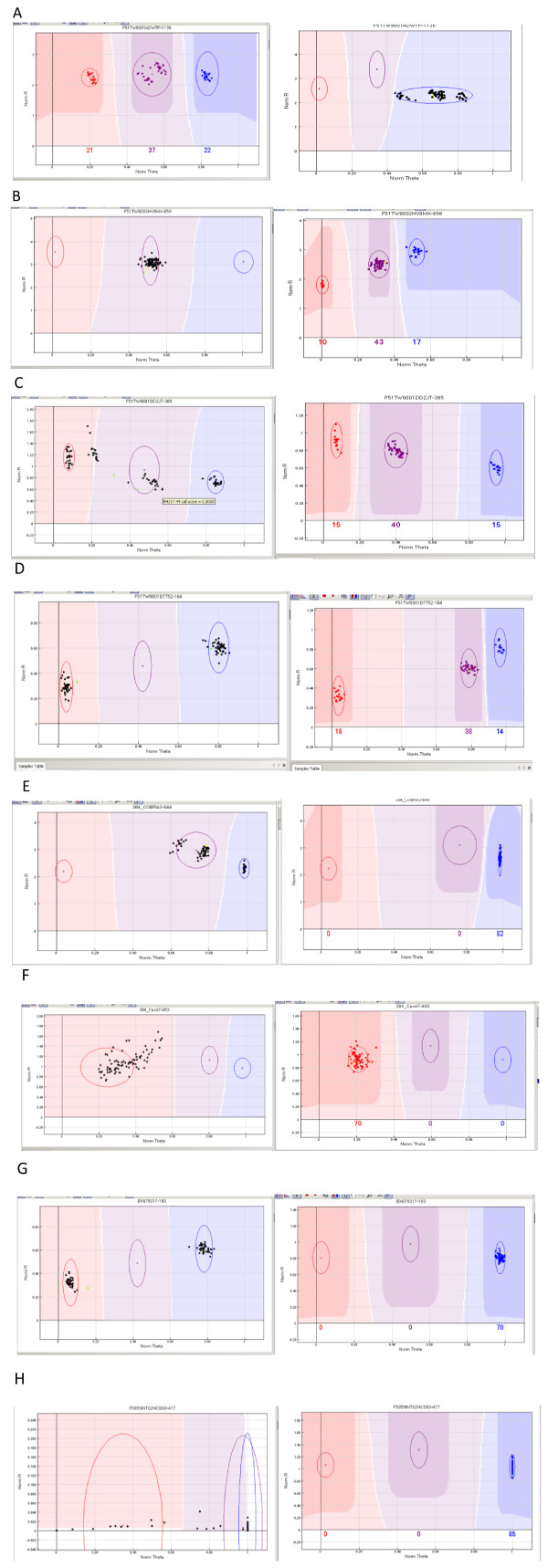
**Illustration of loci giving inconsistent results in the two mapping populations studied (F2 and G2): A, B, C, D polymorphic *versus *failed; E, F, G, H monomorphic *versus *failed.** Counts for each class are available in Additional file 1. *x*-axis (norm Theta; normalized *Theta*) is ((2Π)Tan^-1^(Cy5/Cy3)). Values close to 0 indicate homozygosity for one allele and values close to 1 indicate homozygosity for the alternative allele. *y*-axis (NormR; Normalized R) is the normalized sum of intensities for the two dyes (Cy3 ad Cy5).

Samples that performed poorly were identified by plotting the sample call rate against the 10%GeneCall score. In total, four samples from the G2 population and one sample from the F2 population were found to have low call rates and 10% GC scores and were excluded from further analysis. We thus genotyped 83 and 69 offspring for the G2 and F2 populations, respectively. Poorly performing loci are generally excluded on the basis of the GenTrain and Cluster separation scores obtained when Genome studio software is applied to the whole dataset. In a preliminary study, thresholds of ClusterSep score <0.6 and GenTrain score <0.4 were used to exclude loci with a poor performance. However, visual inspection clearly revealed the presence of SNPs that performed well but had low scores. Conversely, some poorly performing loci had scores above these thresholds. We, therefore, decided to inspect all the scatter plots for the 9,279 SNPs by eye. Three people were responsible for this task and any dubious SNP graphs were noted and double-checked. Overall, 2,156 (23.2%) and 2,276 (24.5%) of the SNPs were considered to have performed poorly in the G2 and F2 populations, respectively. Surprisingly, a significant number of poorly performing SNPs were not common to the two datasets. Cases of well-defined polymorphic locus in one pedigree that performed poorly in the other pedigree could be classified into four categories [see Additional file 1 for their occurrence]:

– Multiple closely located clusters, also referred to as cluster compression (illustrated in Figure 1A). This first category, in which homozygous and heterozygous clusters were closer to each other than expected, accounted for 66.2% of the poorly performing loci in the F2 and G2 pedigrees,

– Heterozygous-like pattern (17%), suggesting the amplification of duplicated loci, with the two paralogs fixed for alternative homozygous genotypes (Figure 1B),

– Presence of more than three clusters or subgroups (15.6%, Figure 1C), suggesting nonspecific amplification of the targeted loci,

– Presence of two clusters in the homozygous configuration (1.2%, Figure 1D), a segregation pattern that was not expected from the parental genotypes.

Similarly, loci monomorphic in one pedigree but performing poorly in the other could be classified into four other categories:

– Cluster compression (Figure 1E), accounting for 79.2% of the poorly performing loci in the F2 and G2 pedigrees.

– Cluster with a scattered distribution (20.4%, Figure 1F)

– Clusters in homozygous configurations (0.2%, Figure 1G)

– Failed assays (0.2%, Figure 1H)

In the G2 pedigree, we found 2,264 polymorphic loci (2,210 SNPs and 54 indels) corresponding to 1,473 PineContig_v2 contigs, including 1,660 SNPs segregating in a 1:1 ratio (760 and 900 SNPs being informative for the female and the male parent, respectively) and 604 SNPs segregating in a 1:2:1 ratio.

In the F2 pedigree, we found 1,215 polymorphic loci (1,184 SNPs and 31 indels) segregating in a 1:2:1 ratio and corresponding to 881 PineContig_v2 contigs.

The conversion rate (number of polymorphic SNPs/indels divided by the total number of SNPs/indels in the assay, that is, 9,279 SNPs) was 24.4% for the G2 population and 13.1% for the F2 population. The conversion rates for SNPs resulting in nucleotide replacement were 35% for the G2 population and 18% for the F2 population, whereas those for 1 bp indel mutations were almost zero (1.8% for G2 and 1% for F2). Indels should, therefore, be avoided when designing an Infinium assay on the basis of 454 reads. Polymorphic SNPs were made available through the National Center for Biotechnology Information (NCBI) dbSNP database [23]. The accession numbers are listed in Additional file 2.

### Validation of the SNP assay

The presence of several SNPs within a single contig made it possible to validate the genotyping assay. For the F2 population, 215 contigs contained more than one SNP. We carried out 22,712 genotyping comparisons and found no genotyping inconsistencies between SNPs from the same contig. Thus, assuming that the probability of crossover between SNPs from the same contig is zero between generations, we obtained a genotyping error of 0%. For the G2 population, 424 contigs contained more than one SNP. We carried out 91,015 genotype comparisons and detected 154 recombination events between SNPs from the same contig, corresponding to a genotyping error of 0.17%. This result confirms the high reproducibility of customized Infinium assays based on careful bioinformatic analysis.

### Comparison of segregation patterns between inbred and outbred mating types

**Figure 2:**
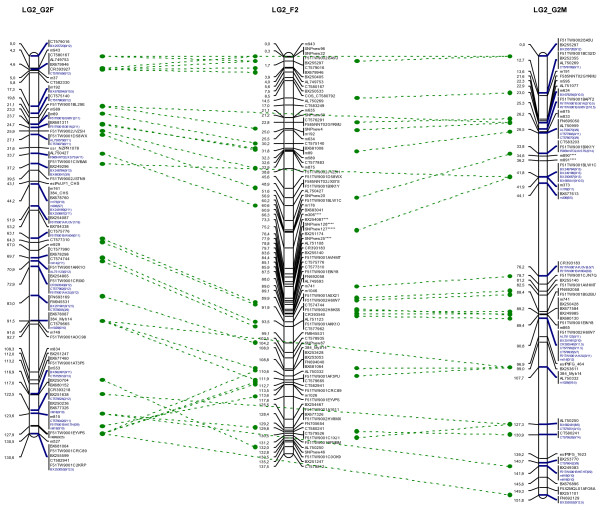
**Example of linkage group (LG2) obtained from segregation data for the G2 and F2 mapping populations.** Markers common to the G2 female (G2F), G2 male (G2M) and F2 maps are linked by green dashed lines. Framework markers (segregating in a 1:1 ratio) are indicated in black, whereas accessory markers (segregating in a 1:2:1 ratio) are indicated in blue followed, in brackets, by the distance (in cM) to the nearest framework marker and the corresponding LOD score. Markers displaying segregation distortion in the initial dataset are indicated with an asterisk (*). The whole map is available in Additional file 3. LOD, logarithm of the odds.

We searched for chromosomal regions containing gene loci with sublethal or lethal alleles, by comparing the segregation pattern for the offspring obtained by outbreeding (G2 pedigree) with that for the offspring obtained by inbreeding (F2 pedigree). This test was based on the assumption that clusters of distorted SNP markers in the F2 progeny that are not distorted in the G2 progeny indicate the presence of lethal or sublethal equivalents revealed by inbreeding. Using a type I error risk of 1%, we initially found 27 distorted markers in the F2 progeny, 25 of which were clustered at three locations: two in linkage group (LG) 10 and one in LG2 [see Additional file 3 and Figure 2]. The number of offspring genotyped in the F2 progeny differed between SNP assays (from 69 F2s for the 12 k SNP assay to 472 F2s for the 384-plex assay) and the segregation data were dependent on sample size. We, therefore, systematically checked clustered distorted SNP markers from the 12 k assay in a larger sample (380 F2s) with a targeted medium-throughput genotyping assay (Mass Array, Sequenom). Only one cluster of four distorted markers remained in LG2 after this validation step [see Additional file 4]. Only two distorted markers gave Basic Local Alignment Search Tool (BLAST) X hits in SwissProt (Probable histone H2A.3 for ‘SNPnew127’ and ‘SNPnew128’ in contig F51TW9001A6567 of PineContigv2). No BLAST hit was found for loci ‘m306’ in contig CL2488CT12CN14 and ‘SNPnew25’ in contig BX254626. A detailed analysis of allele transmission from the grandparental genotypes to the F2 offspring clearly showed that the sublethal allele was inherited from the Corsican paternal grandparent [see Additional file 5].

We also detected segregation distortion in the G2 pedigree for 13 (2.4%) and 6 (1%) markers in the G2F and G2M maps, respectively. One group of eight distorted markers clustered in LG6 of the G2F map, whereas the other distorted loci were distributed more or less evenly, in all LGs. However, it was not possible to verify this cluster of distorted markers in a larger sample, because of the small number of genotypes available for the G2 pedigree. The genetic basis of the segregation distortion may be the abortion of male or female gametes (prezygotic selection) or the selective fertilization of particular genotypes (postzygotic selection). In the case of prezygotic selection, we would expect to see segregation distortion in one of the parental maps only, whereas we would expect to see segregation distortion in the corresponding genomic region on both parental maps for postzygotic selection. If real, given that this hotspot of distorted markers was found only on the G2F map, it could indicate the presence of a locus under female gametic selection.

### Mapping results

#### G2 mapping population

The following numbers of test-cross SNP markers/contigs were available from the 12 k SNP assay in the G2 pedigree [see Additional file 6]: 760 SNPs in 543 contigs (G2F) and 900 SNPs in 615 contigs (G2M), from which a total of 442 and 500 loci (that is, contigs) were mapped as framework markers (map 1, that is, the most reliable map established by JoinMap software, see methods section) in the female and male maps, respectively. Including the markers already available (SNPs, simple sequence repeats (SSRs) and EST-Ps), we eventually mapped a total of 550 and 619 markers on the G2F and G2M maps, respectively, 25 of these loci being common to both maps (indicated by dashed green lines in Figure 2 and Additional file 3). Accessory test-cross (56 and 82 loci for G2F and G2M, respectively) or intercross (409 loci) markers were localized to their most probable framework marker location (indicated in blue in Figure 2 and Additional file 3). Given the low information content assigned to pairs of markers segregating in 1:1 and 1:2:1 ratios [24], only markers showing multiple parallel linkages were retained. Overall, 1,015 and 1,110 markers (mostly corresponding to gene loci) were mapped on the 12 LGs of the female and male maps, respectively [see Additional file 2].

The number of mapped markers per LG (map 1) ranged from 33 to 62 for G2F and from 42 to 62 for G2M, with a mean of 46 mapped markers per LG for G2F and 52 for G2M [see Additional file 7]. The number of linkage groups, 12 on both maps, corresponded to the haploid number of chromosomes.

The length of the linkage groups ranged from 101 to 138 cM for G2F, and 80 to 152 cM for G2M, with mean values of 121 cM for G2F and 119 cM for G2M. Observed genome lengths were 1,447 cM (1 locus/2.6 cM) for G2F and 1,425 cM (1 locus/2.3 cM) for G2M. Expected genome lengths were similar between the two maps, at 1,514 cM for G2F and 1,482 cM for G2M. Observed genome coverage was estimated at 96% for both G2F and G2M, whereas expected genome coverage was close to 100%. There was no correlation between LG length and the number of mapped markers.

#### F2 mapping population

In total, 1,215 SNPs (in 881 contigs) from the 12 k assay and 330 SNPs (in 296 contigs) from previous SNP assays (a 1,536 SNP-assay developed by Chancerel *et al*. [25] and two unpublished 384-SNP assays) were available for mapping [see Additional file 6]. We eventually mapped 1,121 contigs (map 1), with 865 genes from the 12 k assay and 256 from the other SNP assays, onto 13 LGs (LG8 was split into two subgroups). We also mapped 10 other markers (from map 3) as accessory markers [see Additional file 3]. Thus, 1,131 SNP markers were finally positioned on the F2 map.

The number of markers mapped per LG (map 1) ranged from 69 to 122, with a mean of 93 markers per LG [see Additional file 7]. The length of the linkage groups ranged from 115 to 183 cM (183 cM if 50 cM was added to take into account the gap in LG8), with a mean length of 138 cM (142 cM, taking into account the 50 cM gap). Observed genome length was 1,708 cM (1 locus/1.5 cM), which corresponds to an observed genome coverage of 98%. Expected genome length was estimated at 1,745 cM, which corresponds to an expected coverage of 100%. There was no correlation between LG length and the number of mapped markers.

#### Assignment of homologous LGs

LGs that were homologous between F2 and G2F or F2 and G2M maps were identified on the basis of a subset of 198 and 240 common genes, respectively. As expected, a high degree of macrocollinearity was observed over the 12 LGs. However, 15 cases of LG assignment or order discrepancies were identified, suggesting either the presence of paralogous loci (which was obviously the case for two markers mapped to different LGs on the G2 and F2 maps: AL750495 in LG10_G2F and LG8_G2M, and CT577280 in LG7_F2 and LG4_G2M) or a linkage ordering problem (which was the case for 13 non-distorted markers presenting different map locations in homologous linkage groups (BX678432 in LG2_F2 and LG2_G2M, CR393801 in LG4_F2 and LG4_G2F, CT580300 in LG4_F2 and LG4_G2F, m26 in LG4_F2 and LG4_G2F, AL749536 in LG4_F2 and LG4_G2F, m592 in LG4_F2 and LG4_G2F, m593 in LG4_F2 and LG4_G2F, CT577468 in LG4_F2 and LG4_G2F, FN256629 in LG4_F and LG4_G2M, m738/m739/m740 (same contig) in LG7_F2 and LG7_G2M, 384_LIM2 in LG7_F2 and LG7_G2M, BX250169 in LG7_F2 and LG7_G2M, m590 in LG7_F2 and LG7_G2M). These 15 genes were excluded from the list of anchor markers. In addition to the anchor markers between F2 and G2 maps, 25 testcross markers (that is, 25 contigs for which two SNPs were polymorphic in either parent) were used to confirm the homology between LGs on the G2F and G2M maps.

#### Gene density

A Chi^2^ test was performed on the three maps to determine whether the number of genes was evenly distributed between the maritime pine chromosomes. The number of markers per cM (gene density) was found to differ significantly from a uniform distribution between the 12 linkage groups, at the 5% level for G2F and F2 (*P*-value G2F = 0.012, *P*-value F2 = 0.00007), and this difference was just outside the limits of statistical significance for G2M (*P*-value G2M = 0.074). On all three maps, there were fewer genes in LG 8 and a larger number of genes in LG 6 and LG12.

### Factors affecting recombination

**Figure 3:**
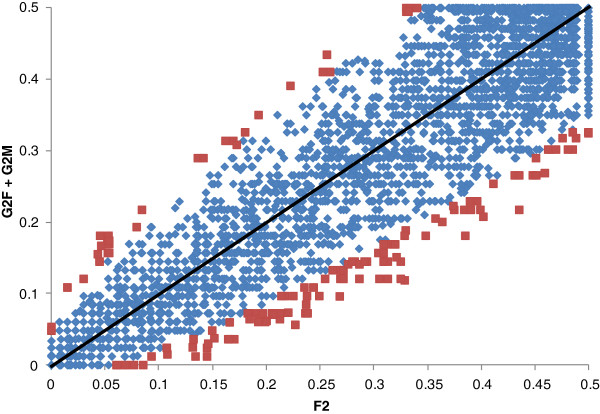
**Scatter plot showing all pair-wise recombination rates between the intraprovenance hybrids (*y *axis: G2F and G2M maps were pooled because no effect of sex was detected) and the interprovenance hybrid (*x *axis: F2 map).** Significant Z-tests are highlighted in red.

We used the Wilcoxon signed rank test to test the hypothesis that ‘map lengths are equal between the three maps: G2F, G2M and F2.’ This hypothesis was not rejected for the comparison between G2F and G2M, *P*-value _(G2F versus G2M)_ = 0.78, indicating that sex had no significant effect on map length in this mapping population. The same test was applied for the comparisons between G2F and F2 and between G2M and F2, with significant differences detected in both cases: *P*-value _(F2 versus G2F)_ = 0.0004 and *P*-value _(F2 versus G2M)_ = 0.005. We checked that the effect of genetic background on the frequency of recombination was not due to the presence of more markers on the F2 map than on the G2F and G2M maps, by carrying out a Wilcoxon signed rank test for all pair-wise recombinations between the common markers in each LG. This test clearly showed that the ‘genetic status’ (intra- versus interprovenance hybrids in our case) of the parental genotypes in which meiotic recombination occurred had a significant effect on the frequency of recombination, with nine LGs presenting significant differences between both F2 and G2F, and F2 and G2M, three LGs presenting a significant difference between F2 and G2F or F2 and G2M, and one LG presenting no effect [see Additional file 8]. Finally, a Z-test was applied to each pair-wise comparison, for the identification of significant pairs among those used to perform the Wilcoxon rank-test (highlighted in red in Figure 3). There was a clear trend toward a greater incidence of significant pair-wise recombination for the F2 map (interprovenance hybrid) than for the G2F or G2M maps (intraprovenance hybrids).

### Distribution of recombination along the chromosomes

**Figure 4:**
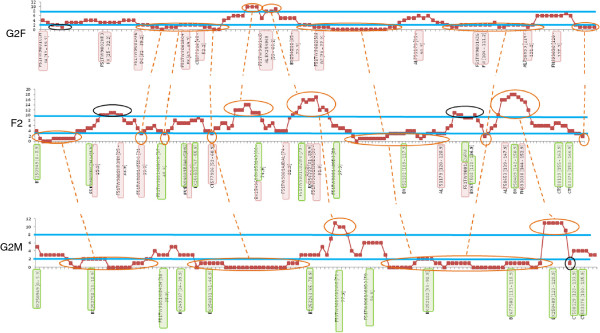
**Representation of marker density for linkage group #3 of the G2F, G2M and F2 maps, highlighting recombination coldspots and hotspots [see Additional file****10****for the whole map].** Marker density was determined by shifting the interval along the map in 1 cM increments. The horizontal lines indicate the lower and upper thresholds defining a gene cluster or a gap. *x*-axis: map distance over the whole linkage group (marker position is as in Additional file 3, with common markers highlighted in green (between G2F and F2) and pink (between G2M and F2), and enclosed in squares for markers common to G2F, G2M and F2). *y*-axis: number of genes in the interval. Clusters common to the F2 map and at least one G2 map are indicated by orange circles connected by dotted orange lines. Clusters common to the G2F and G2M maps are indicated by black circles connected by dotted black lines. Clusters observed on only one map are indicated by black circles.

We also investigated whether the distribution of recombination along the maritime pine chromosomes was affected by the genetic background in which meiotic recombination occurred, by kernel density function analysis. This approach made it possible to set appropriate band widths (per map and per LG) for gene counts, rather than having to fix an arbitrary interval, as in most methods. Based on a comparative analysis of observed and expected marker distributions, we first determined the upper and lower thresholds defining recombination hotspots (larger gaps between markers than expected and coldspots (tightly linked markers), respectively [see Additional file 9]. An analysis of the F2 map showed that a cluster of at least 10 markers (*P* = 3 × 10^-9^) could be considered to constitute a recombination coldspot, whereas a cluster of no more than three markers (*P* = 3.6 × 10^-10^) could be interpreted as a recombination hotspot. For the G2F and G2M maps, recombination coldspots were defined as a cluster of at least eight markers (*P*_*G2F*_ = 0.002; *P*_*G2M*_ = 4.5 × 10^-25^), whereas hotspots were defined as a cluster of no more than two markers (*P*_*G2F*_ = 0.002; *P*_G2M_ = 1.4 × 10^-26^). A plot of gene density over each linkage group, generated by sliding (every 1 cM) an interval corresponding to the predetermined bandwidth, revealed the presence of significant gene clusters or gaps in the three maps (Figure 4 and Additional file 10). By aligning homologous linkage groups, we were able to compare the numbers and locations of recombination coldspots and hotspots between the three maps obtained for the different genotypes (two intraprovenance hybrids for the G2 population and one interprovenance hybrid for the F2 population). We detected a mean of 2.8 coldspots and 5.6 hotspots of recombination per chromosome, respectively. Most (67%) of the hotspots were common to at least two genotypes (27% being common to all three genotypes), but only 48% of the coldspots were common to at least two genotypes (only 7.5% were common to all three genotypes). This result suggests that the spatial structure of recombination is genetically variable, with some recombination hotspots and coldspots specific to a given genotype. Based on the number of shared and specific recombination coldspots and hotspots (Venn diagram in Additional file 10), we calculated a Jaccard index to assess the similarity between the three maps (three pair-wise comparisons). Surprisingly, the recombination patterns of the G2F and G2M maps were found to be more similar to that of the F2 map than to each other.

## Discussion

In this study, we developed modern genomic tools (unigene set, SNP-array and gene-based linkage maps) and applied them to the identification of a deleterious allele segregating at an embryo viability locus, and to studies of the extent and distribution of recombination along the chromosomes and the factors (sex, genetic background) potentially accounting for differences.

### Development of genomic tools to facilitate genetic research in maritime pine

#### Unigene set

In a recent review, McKay *et al*. [5] summarized the transcriptomic resources currently available for the five best-studied coniferous genera. For maritime pine, the first unigene set was derived from 30 k Sanger ESTs and contained 4,483 contigs and 9,247 singletons [25]. A second version (available from [26]) was established with about 0.88 million curated reads, mostly obtained from high-throughput sequencing (454'Roche platform) and assembled into 55,322 unigenes [27]. The third version, presented here, corresponds to the largest sequence data collection obtained to date, with over two million 454 reads assembled into 73,883 contigs and 124,542 singletons. It, therefore, constitutes a major step toward the establishment of a gene catalog for this species. The Roche 454 pyrosequencing platform was chosen because it provides long reads (325 bp in cleaned reads, on average, in this study) that are particularly useful for *de novo* transcriptome assembly, particularly when no reference gene model is available. We will not discuss the content of version#3 further here, because the three datasets were merged together (as they used essentially different sequence reads: Sanger, 454, Illumina) to obtain a large annotated catalog of full-length cDNAs. In the absence of a sequence genome for a conifer, such a catalog will serve as a reference for guiding the assembly of further short-read sequences. This approach is considered the most cost-effective method for both: i) gene expression profiling [28] to determine the molecular mechanisms involved in tree growth and adaptation (for example, [29]); and ii) polymorphism detection [30,31] for applications in evolutionary ecology (for example, [12]), conservation and breeding (for example, [13]). In parallel with the production of *Pinus pinaster* ESTs, the transcriptomes of more than a dozen conifer species were sequenced and assembled [32]. These species included three pine species, but not *Pinus pinaster*. The 1,000 Plant Transcriptome project [33] will also provide transcriptome data for at least 48 conifer species. Overall, this vast body of data will provide a remarkable resource for comparative genomics in conifers, with maritime pine continuing to play a key role in the development of transcriptomic resources for population and quantitative genomics studies.

#### SNP array

Next-generation sequencing of the transcriptome is a powerful strategy for identifying large numbers of SNPs in functionally important regions of the genome [30]. For non-model species, including conifers, this approach is particularly effective when coupled with existing unigene sets, because the reference contigs facilitate the effective assembly of newly generated short reads (as illustrated by Rigault *et al*. [34] and Pavy *et al*. [8] for spruce). In this study, we identified a large number of gene-associated SNPs by *in silico* mining of the maritime pine unigene assembly. It should be noted that the SNPs were selected exclusively from sequence reads associated with cDNA libraries constructed with Aquitaine genotypes. In addition, given the high sequence error rate associated with 454 sequencing (approximately 0.5% [35]), we used stringent criteria (minimum allele frequency (MAF) ≥33%, coverage ≥10x) to avoid the selection of SNPs present at such low frequencies that they are likely to be the product of sequencing error. Consequently, SNPs with low MAFs are less likely to be represented in our genotyping array, and this selection procedure would introduce an ascertainment bias if applied to natural populations from other maritime pine provenances. As our goal was to design a SNP array for use with the Illumina Infinium assay, we also limited our selection to SNPs that were likely to perform well (assay design tool (ADT) score ≥0.75) with this technology, introducing a second bias toward less polymorphic genes, because this score is lower when the flanking sequences contain SNPs. Furthermore, using RNA as the starting material undoubtedly resulted in genes not being equally represented, with highly transcribed genes probably overrepresented in our sample.

For the 6,299 nucleotide replacement SNPs, 25% failed and 40% to 57% were monomorphic, depending on the population, whereas 19% of the assays failed and 80% of the markers were monomorphic for insertion-deletion mutations. Thus, indel mutations are more prone to sequencing errors with the Roche sequencing platform and should clearly be avoided in the Infinium assay. Taking into account only the markers polymorphic in both of the pedigrees studied, 1,970 different gene loci were successfully tagged with at least one SNP and mapped (either as framework or accessory markers) within the genome.

#### Linkage maps

High-density linkage maps are crucial to our understanding of quantitative trait variation, especially for species without a reference genome assembly. With the recent development and thorough assessment of SNP markers, saturated, high-density genetic linkage maps have been established for several conifers, including *Cryptomeria japonica* (1,216 markers, 968 corresponding to SNPs, over 1,405cM, [36]), *Picea mariana* and *Picea glauca* (consensus map of these two species comprising 1,801 gene loci over 2,083 cM, [8]), *Pinus taeda* (1,816 genes over 1,898cM, [9]) and *Pinus pinaster* (this study). As in these aforementioned studies, the expected map coverage rate for the maritime pine linkage maps was high (about 100%), indicating that the maps developed in this work are saturated. Thus, the mean distance between adjacent markers (2.6, 2.3 and 1.5 cM in the G2F, G2M and F2 maps, respectively) was strongly skewed toward small distances [see Additional file 11]. These next-generation linkage maps will facilitate the analysis of conifer genome evolution, by making comparative mapping possible at a scale that was not achievable with previous, low-throughput marker systems (for example, [37]).

### Comparison of segregation patterns between inbred and outbred matings indicates the presence of a chromosomal region with a deleterious mutation acting at the postzygotic stage

Departure from Mendelian expectations, which is also known as segregation distortion (SD, [38]), is frequently reported in linkage mapping studies (reviewed by Li *et al*. [39]). If a gene causing SD is segregating in a population, then the markers close to it tend to display distorted segregation ratios. Thus, as a rule of thumb, the clustering of markers displaying SD in particular genomic regions (so-called segregation distortion regions, SDRs) may indicate that segregation distortion is caused by genetic factors rather than statistical bias or genotyping errors. However, as illustrated in this study, small population size may lead to false positives and the identification of spurious SDRs. Care should therefore be taken to validate SDRs before any biological interpretation is attempted.

Biologically, aberrant Mendelian segregation can be attributed to selection occurring at different stages of the plant’s life cycle, from gametophyte development to seed germination and plant growth [20,40]. In this study, a single cluster of distorted markers was detected and validated in LG2 of the F2 map, whereas the corresponding genomic region on the two G2 maps displayed no deviation from the expected Mendelian segregation ratio. This strongly suggests the presence of a deleterious mutation (or a cluster of tightly linked embryo viability loci), revealed by inbreeding, that influences the fitness of the F2 zygotes at some point between fertilization and the age of 10 years (as the tissues sampled for DNA extraction were taken from 10-year-old trees). This conclusion is supported by two additional observations. First, this F2 family was selected specifically because of its low rate of seed abortion (frequency of embryo-less seeds, as estimated by assessing floating in water, was lower than for other available F2s from the maritime pine breeding program, unpublished results), making it particularly suitable to genetic analysis requiring a large sample size. In our study, 638 seeds were initially planted in a nursery in June 1998; 626 seedlings germinated (that is, only 1.9% died soon after germination) and were transplanted into the field in March 1999. Total height was then measured every fall, beginning in 1999. Fifteen seedlings died during the first growing season in the field (assessment in the fall of 1999). The following year, 43 other seedlings died, but no further deaths were recorded thereafter. It is difficult to determine whether these deaths were due to some crisis during transplantation from the nursery to the field or to genetic load. However, peak mortality did not occur in the nursery or just after field transfer, and the semilethal allele was inherited from the Corsican paternal grandparent. These findings suggest that this SDR decreases the fitness of homozygous Corsican genotypes in early stages of development and later in tree growth. Unfortunately, no post-mortem analysis involving the sampling of plant material from the whole progeny just after germination was performed, to determine whether the dead plants were all homozygous for the Corsican allele in the SDR concerned.

Second, in a previous study, Plomion *et al*. [41] compared the segregation patterns of random amplified polymorphic DNA (RAPD) markers in megagametophytes (a maternally derived haploid tissue surrounding the embryo) from the same hybrid tree (H12), sampled from either inbred (self-cross) or outbred (open-pollinated cross) seeds. They observed no significant SD for loci in the dataset resulting from selfing, suggesting that gametic selection, leading to gamete abortion or lower gamete fitness, can be ruled out as a possible cause of SD in this study.

Genomic regions containing lethal or sublethal alleles have already been detected in several conifers, through linkage mapping approaches (reviewed in Williams [21]). The number of such lethal or sublethal equivalents is generally high in populations, as revealed by the typical high level of inbreeding depression in these outcrossing species (reviewed by Williams and Savolainen [42]), but their severity varies in the population, with some genotypes (like that selected in this study) bearing mutations that are, *a priori,* less deleterious than others. The nature of the underlying loci remains unclear. Some of these genetic factors are involved in early embryo development, resulting in a lower yield of filled seeds upon selfing, others decrease seedling growth and cause abnormal phenotypes, whereas others are directly involved in seedling mortality at later stages of development, from a few weeks [43] to a few months after germination, as shown here. In addition to providing fundamental knowledge, the analysis of segregation distortion and the identification of SDR are of great importance for the correct determination of quantitative trait loci (QTL) positions and for the estimation of QTL effects. Indeed, SD influences the estimation of recombination frequency and may, therefore, decrease the accuracy of QTL mapping in this mapping population.

### The extent and spatial distribution of meiotic recombination is genetically variable

Recombination is a driving force behind the generation of genetic diversity and is also a key process shaping genomic architecture [19]. An understanding of the factors controlling the frequency and genomic distribution of meiotic recombination is, therefore, essential if we are to manipulate this process to improve breeding accuracy. This study generated three major results.

First, we confirmed that, despite their large physical size, pine chromosomes display a similar number of crossover events to other smaller plant chromosomes. This observation led Thurieaux [44] to suggest that recombination was confined largely to the coding regions, because all eukaryotes have approximately the same number of genes, as demonstrated by the genome sequences of various organisms (for example, in *Arabidopsis*[45], rice [46,47], maize [48] and sorghum [49]), although other genomic features may affect recombination. At the microscale, no consistent relationship has yet been established between recombination rate and gene content [50,51], suggesting that it is probably not correct to assume that all plant recombination hotspots correspond to gene-rich regions. It will not be possible to determine whether recombination hotspots correspond to gene-rich regions in conifers until a complete conifer genome sequence is obtained. However, as gene-rich regions tend to be associated with high rates of recombination in other plants, it seems likely that relationships between crossover frequency and gene density will not deviate from this trend in conifers. For example, in bread wheat (17Gb/C), a non-uniform crossover gradient along chromosome 3B has been observed, with lower frequencies of crossover in the gene-poor centromeric region and the highest frequencies of crossover in the distal subtelomeric regions, in which gene density is higher [52]. At a finer scale, these authors also demonstrated that gene content was one of the factors driving recombination in this species [53].

Second, we observed that meiotic recombination was not randomly distributed along the length of the maritime pine chromosomes, suggesting that recombination occurs at specific sites, the recombination hotspots (reviewed by Lichten and Goldman [54]). An uneven distribution of markers is a classical observation in most papers reporting saturated linkage maps for plants and animals. Tests of departure from a Poisson distribution have always been based on a single or a series of different, arbitrarily fixed intervals (as illustrated by Moriguchi *et al.*[36] in *Cryptomeria japonica*). To our knowledge, only Pavy *et al*. [8] have previously implemented a statistical approach, based on kernel density function, in *Picea spp*., to overcome the need to use such fixed bandwidths in analyses of ‘gene-rich regions’ as an indicator of suppressed recombination. In this study, we used the same strategy, combining it with a sliding window approach, to improve the resolution of recombination hotspots and coldspots. Interestingly, in most LGs of the G2F and G2M maps, a sharp cold spot located in the middle of the linkage group was surrounded by two large hot spots. This suggests that these cold spots may correspond to the centromeric regions of the chromosome, in which the frequency of recombination is known to be low [51-55] and in which markers tend to cluster on meiotic maps. However, further studies are required to confirm this assertion. This signature was less clear in the F2 map, which contained about twice as many coldspots as the G2 maps (48 in F2 versus 27 in G2F and 28 in G2M), with a similar number of hotspots (71 versus 62 and 69). An uneven distribution of crossover events has been reported for both species with small genomes and those with large genomes ( [51] for *Arabidopsis*, [53] for wheat) and an understanding of the distribution of recombination events is critical for various genetic applications. First, following on from the discussion above, if recombination occurs in hotspots and these hotspots bear most of the genes, then differential sequencing efforts will be required to obtain data for all of the genes in conifer genome sequencing programs. Second, as illustrated by Wang *et al*. for rice [56], the map-based cloning of a QTL is facilitated if the QTL is located in a genomic region containing a recombination hotspot, simply because it is easier to identify large numbers of recombinants from segregating populations. This information may be useful for the characterization of genes underlying major QTLs in species with large genomes, such as pines, as already reported for wheat [57].

Third, our results show that the extent and spatial distribution of meiotic recombination is genetically variable. The interprovenance hybrid had recombination rates 1.2 times higher (measured on the basis of total map distance) than those of either of the intraprovenance hybrids. This suggests that the genetic divergence of bivalents may account for the extent of recombination at meiosis. However, a comparison of gene heterozygozity between the three genotypes on the basis of both mapping data [see Additional file 6] and the *in silico* prediction of polymorphisms [see Additional file 12] showed that the diversity of the interprovenance hybrid was intermediate with respect to the diversity of the two intraprovenance hybrids. These two findings indicate that the genetic distance (at least within the gene space, in which most crossover events are thought to occur) between the bivalents does not alter meiotic pairing to a point that would lead to differences in recombination frequencies, as shown in interspecific hybrids by *in situ* hybridization [58] and linkage mapping [59]. Moreover, the high degree of collinearity between the maps for the intra- and interprovenance hybrids shows that no genome rearrangement occurred during hybridization that might have led to a recombination disorder. We can conclude that the observed difference in map length reflects differences between genotypes. The distribution of recombination events differed between the three genotypes, which had only some hotspots, and even fewer coldspots in common. This suggests that the spatial pattern of recombination along the chromosome is also genetically variable and under polygenic control, as demonstrated by Comeron *et al.*[16] in *Drosophila melanogaster*. Recombination is known to be genetically variable [15,60,61] and under the control of multiple *trans* and *cis* genetic modifiers. Sequence polymorphisms [62,63] and/or the methylation status of these genetic factors may underlie these differences in recombination pattern and should be investigated further in conifers.

Whether the results obtained depend on the type of markers used needs to be addressed. First, it should be noticed that the total map length obtained in the present study with coding sequences, was similar to that obtained for the same genotypes using anonymous RAPD [41] or amplified fragment length polymorphism (AFLP) [3] markers (supposedly corresponding to non-coding DNA). Second, maps combining gene-based markers and genomic DNA markers (for example, proteins and RAPDs in [64], EST-Ps and AFLPs in [37], SNPs and AFLPs in [25]) were also constructed in this species and did not show any clustering of one or another marker type. Therefore, it is assumed that the recombinational landscape presented in this paper should not be biased by the type of marker (coding *versus* non-coding) used for linkage analysis.

## Conclusion

We present the most comprehensive unigene set to date for maritime pine and three SNP-based linkage maps at a much higher resolution than previously published for this species. The two major findings of this study are: i) a hotspot of recombination, identified on the bases of SD for various markers in analyses of the segregation data obtained for the inbred and outbred pedigrees, revealed the presence of a region containing a semilethal recessive allele inherited from the Corsican grandparental genotype. The localization of this zygotic lethal factor will be of key importance for the interpretation of the effect of the QTL in further studies using this unique F2 progeny to dissect the genetic architecture of quantitative traits; and ii) the extent and distribution of recombination along the chromosomes at a cM resolution was found to be genetically variable and not related to the genetic distance between parental genotypes. Our data provide the first insight into the intraspecific variation of recombination in a conifer species.

These three gene-based linkage maps have been merged, to provide a composite map on which the distribution of genetic diversity and linkage disequilibrium along the chromosomes was studied. They will also provide positional and functional candidate genes, within the QTL region for important traits such as water use efficiency.

## Methods

### Genetic material and DNA extraction

The two mapping populations (G2 and F2, Additional file 13) used in this study have been described elsewhere [25]: G2 is a three-generation outbred pedigree (full-sib progeny), whereas F2 is a three-generation inbred pedigree. Young needles of each individual were harvested and stored at −80°C until DNA extraction. Pieces of frozen needles (around 30 to 40 mg) were crushed using a mixer mill (Retsch MM300, Haan, Germany). Genomic DNA was isolated with the Invisorb Plant DNA 96 kit from Invitek GmbH (Berlin, Germany), according to the manufacturer’s instructions. All concentrations were determined with a Nanodrop spectrophotometer (NanoDrop Technologies, Wilmington, DE, USA) and fluorescence assays (Quant-IT kit, Invitrogen, Carlsbad, CA, USA). All samples with concentrations exceeding 50 ng/μl (based on fluorescence measurements) were kept and sent to Genediffusion (Pasteur Institute, Lille, France) for Infinium assays on the iScan platform (Illumina Inc., San Diego, CA, USA).

### Bioinformatic analysis

#### Development of genomic resources

Sanger ESTs were available from the sequencing of 12 suppressive subtractive hybridization (SSH) and/or conventional cDNA libraries [25]. In this study, pyrosequencing (454 titanium, Roche, Branford, CT, USA) was also conducted for 15 additional cDNA libraries. A description of the libraries generated is provided in Additional file 14. It should be noted that (i) maritime pine libraries were derived from several genotypes and correspond to different tissues (differentiating xylem, roots, bud, needles and somatic embryo) or different experimental treatments (that is, drought-stressed plant), and (ii) that most of the sequences (97%) were obtained by the pyrosequencing approach. In addition, 2,358 Sanger ESTs (libraries 26235, 26097 and 12219 in Additional file 14) were recovered from the NCBI dbEST and Genbank databases.

#### Cleaning procedure

All 454 reads were produced with the Smart PCR cDNA synthesis kit. Data were cleaned with the SmartKitCleaner and Pyrocleaner tools [65], based on the following steps: i) clipping of adaptors with cross_match [66]; ii) removal of reads outside of the length range (150 to 600); iii) removal of reads with a percentage of Ns greater than 2%; iv) removal of reads with low complexity, based on a sliding window (window: 100, step: 5, min value: 40). All Sanger reads were cleaned with Seqclean [67]. After cleaning, 2,016,588 sequences were available for the assembly.

#### Assembly procedure and annotation

Sanger sequences and 454-reads were assembled with the SIGENAE pipeline [68] based on TGICL software [67], with the same parameters described by Ueno *et al*. [69]. This software uses the CAP3 assembler [70], which takes into account the quality of sequenced nucleotides when calculating the alignment score.

The resulting unigene set was called ‘PineContig_v2’. This unigene set was annotated by BLAST analysis against the following databases: i) Reference databases: UniProtKB/Swiss-Prot Release August 2010, UniProtKB/TrEMBL Release August 2010, RefSeq Protein of 8 June 2010, Pfam Release 24.0 of July 2009 and RefSeq RNA of 8 June 2010; and ii) species-specific TIGR databases: *Arabidopsis* AGI 15.0, *Vitis* VvGI 7.0, *Medicago* MtGI 10.0, TIGR *Populus* PplPGI 5.0, *Oryza* OGI 18.0, *Picea* SGI 4.0, *Helianthus* HaGI 6.0 and *Nicotiana* NtGI 6.0.

Repeat sequences were detected with RepeatMasker. Contigs and annotations can be browsed and data mining carried out with BioMart, at [22].

#### Detection of nucleotide polymorphism

**Figure 5:**
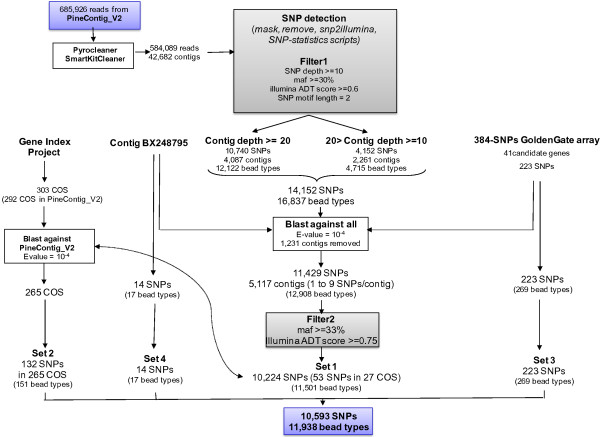
**Flowchart describing the steps in the identification of SNPs in the Aquitaine population.** PineContig_V2 is the unigene set developed in this study. ADT, Assay Design Tool; COS, comparative orthologous sequence; MAF, minimum allele frequency.

Four subsets of this vast body of data (detailed below) were screened for the development of the 12 k Illumina Infinium SNP array. A flowchart describing the steps involved in the identification of SNPs segregating in the Aquitaine population is shown in Figure 5.

• *In silico* SNPs detected in Aquitaine genotypes (set#1). In total, 685,926 sequences from Aquitaine genotypes (454 and Sanger reads) derived from 17 cDNA libraries were extracted from PineContig_v2 [see Additional file 15]. We focused on this ecotype of maritime pine because our long-term objective is to carry out genomic selection in the breeding program focusing principally on this provenance. Data were cleaned with the SmartKitCleaner and Pyrocleaner tools [65]. The remaining 584,089 reads were distributed into 42,682 contigs (10,830 singletons, 15,807 contigs with 2 to 4 reads, 6,871 contigs with 5 to 10 reads, 3,927 contigs with 11 to 20 reads, 5,247 contigs with more than 20 reads, Additional file 16). SNP detection was performed for contigs containing more than 10 reads. A first Perl script (‘mask’*)* was used to mask singleton SNPs [71]. A second Perl script, ‘Remove’*,* was then used to remove the positions containing alignment gaps for all reads. The number of false positives was minimized by establishing a priority list of SNPs in the assay on the basis of MAF, depending on the depth of each SNP. Finally, a third script, ‘snp2illumina’*,* was used to extract SNPs and short indels of less than 7 bp, which were output as a SequenceList file compatible with Illumina ADT software. The resulting file contained the SNP names and surrounding sequences with polymorphic loci indicated by IUPAC codes for degenerate bases. We generated statistical data for each SNP — MAF, minimum allele number (MAN), depth and frequencies of each nucleotide for a given SNP — with a fourth script, ‘SNP_statistics’. We established the final set of SNPs by considering as ‘true’ (that is, not due to sequencing errors) all non-singleton biallelic polymorphisms detected on more than five reads, with a MAF of at least 33% and an Illumina score greater than 0.75 (Filter 2 in Figure 5). Based on these filter parameters, 10,224 polymorphisms (SNPs and 1 bp insertion/deletions, referred to hereafter as SNPs) were detected

• *In silico* SNPs detected in comparative orthologous sequences (COS) between pine and spruce (set#2). For the COS between pine and spruce identified by Chancerel *et al*. [25], 303 resulted in a hit with at least one maritime pine EST. By blasting these sequences against PineContig_v2 (BlastN, Evalue = 10^-4^), we identified 292 contigs containing 185 COS markers, 53 of which were already present in set#1, and 132 of which were specific to set#2.

• *In vitro* SNPs selected from a previous SNP array (set#3). In total, 223 SNPs originally detected and validated in a set of 41 resequenced candidate genes (*in vitro* SNPs [71]) were selected for the 12 k SNP assay.

• SNPs detected in a gene fragment including at least one SNP associated with growth (set#4). An SNP associated with height growth has been identified [72]) in contig CT-3782 of the first *Pinus pinaster* assembly described by Chancerel *et al*. [25]. By blasting the CT-3782 contig sequence against PineContig_v2 (BlastN, Evalue = 10^-4^), we identified BX248795 as homologous to CT-3782. We found 14 *in silico* SNPs in this contig, which were included in the 12 k SNP assay.

Finally, based on these four different SNP sets, 10,593 SNPs (corresponding to 11,938 bead types, Additional file 17) were available for genotyping after filtering with the ADT of Illumina. All but three of the SNPs had a score above 0.63.

### SNP genotyping assay

Genotyping was carried out at Genediffusion (Institut Pasteur, Lille, France) with the Illumina Infinium assay, used according to the manufacturer’s instructions (Illumina). In total, 87 and 70 offspring were initially genotyped for the G2 and F2 mapping populations, respectively. The Infinium assay is based on the direct hybridization of genomic targets to array-bound sequences. Single-base extension is followed by fluorescence staining, signal amplification, scanning and analysis with Genome Studio software v. 1.0. From the initial set of 10,593 SNPs, 1,314 (12.4%) did not pass Illumina production quality control and were eliminated. The remaining 9,279 SNPs (6,299 SNPs *sensu stricto* and 2,980 indels distributed in 4,613 different contigs) were individually inspected with Genome Studio software, with a GenCall score cutoff of 0.15 (according to Illumina’s recommendations) to detect failed, monomorphic and polymorphic SNPs. We considered loci for which two or three scatter plots (depending on the type of marker segregation) were identified without ambiguity to be polymorphic markers. SNP clusters were modified manually, to refine cluster positions when necessary.

### Analysis of segregation distortion

For each locus, we tested the pattern of allelic segregation for goodness-of-fit to expected Mendelian segregation ratios, in Chi^2^ tests (*P* ≤0.01). We assumed that clusters of distorted loci in the F2 inbred progeny that were not distorted in the G2 outbred progeny indicated the presence of a deleterious allele revealed by inbreeding. Given the small number of F2 progenies (69) genotyped with the 12 k SNP array, markers displaying SD were examined on a larger and independent sample of F2s (380 trees), to check for the presence of hotspots of SD. We used the medium-throughput MassARRAY iPLEX genotyping assay from Sequenom (San Diego, CA, USA) for this purpose.

In total, 34 SNPs — 25 distorted (in 19 contigs) and 9 non distorted markers (in 9 contigs distributed in different LGs) — in the F2 progeny were included in two multiplex assays (22plex and 12plex, Additional file 18) with MassArray assay design 4.1 software (Sequenom). Six pairs of SNPs displaying SD and located in the same contig were used in the assay, to assess the reproducibility of this genotyping method. Four pairs were successfully genotyped and showed no genotyping inconsistencies. The hybrid parent (H12) used as a positive control also displayed no genotyping inconsistencies, confirming the high degree of reproducibility of the iPLEX GOLD method.

DNA extraction and quantification were carried out as described above. In total, 15 ng of DNA was required for the reaction. Genotyping was carried out at the Genomic and Sequencing Facility of Bordeaux (France), with the iPLEX Gold genotyping kit (Sequenom), according to the manufacturer’s instructions. The iPLEX Gold SNP genotyping method involves several steps: PCR amplification is carried out first, followed by SAP treatment (to digest unincorporated dNTPs). A single-base extension reaction is then performed, followed by an ion-exchange cleanup step. Finally, the products are detected in a MassArray mass spectrophotometer and the data are acquired in real time with MassArray RT software. Alleles were automatically assigned by MassArray TyperAnalyser 4.0.22 software and associated with a reliability value. Positive (hybrid parent of the F2 mapping population) and negative controls were including in the genotyping process. Visual inspection was carried out for all the SNPs, to detect any incorrect assignments made by the ‘Autocluster’ option of the MassArray Typer Analyser software. Finally, locus segregation was tested for goodness of fit to expected Mendelian segregation ratios, in Chi^2^ tests (*P* ≤0.01).

### Linkage mapping strategy

For linkage analysis, we retained only one SNP if several were present with the same contig.

#### G2 pedigree

Genetic linkage analysis was performed by the ‘two-way pseudotestcross’ mapping strategy [73]. Linkage maps were constructed for each parental tree (female accession 9.106.3 and male accession 10.159.3). The polymorphic SNPs of the 12 k SNP array were combined with 380 other markers [see Additional file 19-A] including 299 SNPs from a previous 1,536 SNP assay [25], 50 EST-polymorphisms (EST-Ps) and 31 SSRs [37,74]. Conformity to Mendelian segregation ratios was evaluated in Chi^2^ tests (*P* <0.01) and linkage analysis was performed with JoinMap v 4.1 [75], using CP (cross pollination) as population type and a LOD threshold ≥3. Phases (coupling and repulsion) of the marker loci were detected automatically by JoinMap, with the ‘CP’ option, which allows loci of different phases to be linked on the same chromosome.

The mapping procedure was as described by Chancerel *et al*. [25]. Briefly, we used the regression algorithm (with Kosambi mapping function), which generally generates three different maps with different levels of statistical support (map 1, map 2 and map 3, in decreasing order of statistical support). All test-cross markers segregating in a 1:1 ratio (including those displaying SD) were taken into account. For each parental map, we retained map 1, on which we positioned, as accessory markers, the additional markers mapped in map 3 and less informative intercross markers segregating in a 1:2:1 ratio. The relative position of each accessory marker with respect to its most probable location was determined on the basis of two-point LOD scores and recombination frequencies, which were obtained from the ‘Maximum linkage’ table of JoinMap. Hereafter, the resulting linkage maps will be named G2F and G2M, for the female and male parents, respectively. Linkage groups were named as in the study by Chancerel *et al*. [25].

#### F2 pedigree

The map of the interprovenance hybrid tree (H12) that was selfed to generate the F2 mapping population was constructed on the basis of three different SNP assays [see Additional file 19-B]: a previous 1,536plex [25] providing 193 SNPs, two 384-plexes developed for QTL analysis (unpublished) and providing 137 SNPs, and the 12 k Infinium assay described here. All polymorphic markers segregated in a 1:2:1 ratio in the progeny (heterozygous in the F1 parent), and locus segregation was tested for goodness of fit to expected Mendelian segregation ratios, in Chi^2^ tests (*P* ≤0.01). Linkage analysis was conducted with JoinMap v4.1, using F2 as the population type. Marker order and relative genetic distances were calculated by the regression mapping algorithm, with the following parameters: Kosambi mapping function and a LOD threshold ≥3. We retained map 1, on which we positioned, as accessory markers, the additional loci mapped in map 3. The position of each accessory marker relative to its most probable location was determined on the basis of the two-point LOD scores and recombination frequencies available from the ‘Maximum linkage’ table of JoinMap. Linkage groups were named as in the study by Chancerel *et al*. [25]. The linkage map of the interprovenance hybrid tree is referred to as the F2 map.

### Estimation of genome length and map coverage

Observed genome length (G0) was calculated as the sum of the map lengths of all linkage groups. As LG8 was divided into two parts in the F2 pedigree, we added 50 cM to G0 to account for this gap. Expected genome length (G_e_) was calculated by method#4 of Chakravarti *et al*. [76], as G_e_ = Σ (length of the linkage groups * (m + 1)/(m-1)), where m is the number of markers in map 1. This estimation assumes a uniform distribution of map locations. Observed map coverage (C_o_) was calculated as the ratio of observed and estimated genome lengths. Expected genome coverage (C_e_) was calculated as described by Bishop *et al*. [77]:

$$\begin{array}{l} C_{e}=1-[2R/\left(N+1\right)\left\{{\left(1-X/\left(2*G_{e}\right)\right)}^{N+1}-{\left(1-X/G_{e}\right)}^{N+1}\right\}\\ \;+\left(1-\left(R*X\right)/G_{e}\right)*{\left(1-X/G_{e}\right)}^{N}], \end{array}$$

where R is the haploid number of chromosomes (12 in our case), N is the number of loci positioned on map 1, X is the maximum observed map distance between two adjacent markers in cM, at or above a minimum LOD threshold value of 6, 7 and 8. X was set to 50 cM for the F2 maps, to take into account the splitting of LG8 into two subgroups.

### Analysis of marker distribution and comparison of recombination frequencies

#### Distribution of mapped genes between chromosomes

We first tested whether the mapped genes were evenly distributed between the linkage groups, by comparing observed and estimated numbers of genes per linkage group in a Chi^2^ test (*P* <0.05). The expected number of genes for each LG was obtained by multiplying the ratio ‘size of LG/total genome length’ by the total number of mapped genes (map 1).

#### Distribution of mapped genes along chromosomes

Gene distribution was then analyzed to determine whether the mapped markers (in map 1) were uniformly distributed within each of the LGs of each map (G2F, G2M, F2) or whether they displayed some kind of clustering. To this end, we used a kernel density function to calculate an optimized window size (bandwidth) for dividing the genome into blocks, in which the number of genes was determined. Kernel density estimation is a nonparametric technique for density estimation in which a known density function (here, a Gaussian function) is averaged across the observed data points to create a smooth approximation. The smoothness of the density approximation depends on the bandwidth. In our case, we used a fixed and robust bandwidth estimator [78], based on the algorithm of Jones *et al*. [79]. Bandwidth values [see Additional file 20] were calculated for each linkage group of each map independently and the distribution of gene density was plotted for each linkage group, by sliding (every 1 cM) an interval corresponding to the genomic bandwidth.

Marker distribution was then analyzed by comparing the observed distribution of the number of markers per block with that expected under a Poisson distribution (*P*(*X* = *k*) = *λ*^*k*^*e*^− *λ*^/*k* !), where λ is the mean number of markers per block and k varies from 0 to 10 markers per block) in Chi^2^ tests. The results of Chi^2^ tests may be inaccurate for small expected numbers, so this test was carried out with the data for the whole map rather than on a per linkage group basis. A lower threshold defining recombination coldspots was defined as the point at which the observed number of markers exceeded the number expected, and Chi^2^ test results remained significant. Similarly, an upper threshold defining recombination hotspots was defined as the point at which the observed number of markers was lower than expected, and Chi^2^ test results remained significant.

#### Comparison of recombination rates between genotypes

We used two statistical tests to compare recombination rates between the different genotypes (two intraprovenance hybrids: 9.106.3 and 10.159.3, and one interprovenance hybrid: H12) from which linkage maps were constructed. Wilcoxon signed rank tests with continuity correction were first performed to test the following hypotheses: i) ‘Map lengths (based on LG sizes) are not significantly different between G2F, G2M and F2’ (that is, test for sex and genetic background effects); and ii) ‘Recombination rate (based on pair-wise data) is not significantly different between F2 and G2F or between F2 and G2M’ (that is, test for genetic background effect only). This second hypothesis was tested with markers common (without segregation distortion) to the two pedigrees only. The number of markers common to G2F and G2M was too small for the testing of this hypothesis (that is, sex effect) with pair-wise recombination data. The ‘Maximum linkage’ table of JoinMap provided two-point recombination frequencies and the Wilcoxon signed rank test was performed for each linkage group of each map and for the entire genome.

A Z-test was then performed to test the null hypothesis that there was no significant difference in recombination rate for any of the marker pairs common to all three genotypes.

$$Z=\left(01-02\right)/\sqrt{\left(01\left(1-01\right)/n1+02\left(1-02\right)/n2\right)}$$

where:

– θ1 is the recombination rate in F2

– θ2 is the recombination rate in G2F or G2M

– n1 is the mean number of informative meioses in F2 (that is 69 of 70)

– n2 is the mean number of informative meioses in G2 (that is 83 of 87)

Scatter plots showing all pair-wise recombination rates were finally obtained for each LG and for the whole genome. Significant Z-tests are highlighted.

### Availability

The browsing of maritime pine contigs, annotations and SNPs and data mining by BioMart can be carried out at [22].

Information about the linkage maps is available from the PinusMap database, available from [80] and the Pine Cmap database, available from [81].

Roche 454 sequencing data are available at the short-read archive of the NCBI database [82].

Polymorphic SNPs are available from the NCBI dbSNP database [23]. Accession numbers are listed in Additional file 2.

## Abbreviations

ADT: Assay design tool; AFLP: Amplified fragment length polymorphism; BLAST: Basic local alignment search tool; bp: Base pair; cM: Centimorgan; COS: Comparative orthologous sequences; CP: Cross pollination; ESTs: expressed sequence tags; LG: Linkage group; LOD: Logarithm of the odds; MAF: Minimum allel frequency; MAN: Minimum allel number; PCR: Polymerase chain reaction; QTL: Quantitative trait loci; RAPID: Random amplified polymorphism DNA; SD: Segregation distortion; SDR: Segregation distortion region; SNP: Single nucleotide polymorphism; SSH: Suppressive subtractive hybridization; SSRs: Simple sequence repeats.

## Competing interests

The authors declare they have no competing interests.

## Authors’ contributions

PL, GLP, CL and VL sampled plant material, extracted total RNA and constructed the cDNA libraries; CK and CN assembled the Sanger sequences and 454 ESTs and made the unigene set available in a EnsEmbl-like browser; IL identified *in silico* SNPs in the maritime pine EST database; EC sampled plant material, extracted DNA and checked DNA quality; CB, EC and FS developed and performed the iPlex mass array genotyping; EC, JBL, JMG, and CP analyzed the data; FE developed the PinusMap database; CP wrote the manuscript, conceived, designed and coordinated the project. All authors read and approved the final manuscript.

## Supplementary Material

Additional file 1 **Occurrence of loci displaying inconsistent failure in the two mapping populations studied (F2 and G2): A, B, C, D, polymorphic vs. failed; E, F, G, H monomorphic vs. failed (see illustration in Figure** 1**).** Click here for file

Additional file 2 List of SNP markers with dbSNP accessions, corresponding contig ID in PineContig_v2, and linkage group assignment on the G2F, G2M and F2 linkage maps. Click here for file

Additional file 3 **Genetic linkage maps obtained from segregation data for the G2 and F2 mapping populations.** Markers common to the female (G2F), male (G2M) and F2 maps are linked by green dashed lines. Framework markers (segregating in a 1:1 ratio) are indicated in black, whereas accessory markers (segregating in a 1:2:1 ratio) are indicated in blue, followed, in brackets, by the distance (in cM) to the nearest framework marker and the corresponding LOD score. Markers displaying segregation distortion in the initial dataset are indicated with an asterisk (*). Click here for file

Additional file 4 **Segregation pattern in the F2 progeny before and after validation on a larger sample size.** Only SNPs from the 12 k array were genotyped by the iPLEX Sequenom assay. Click here for file

Additional file 5 **Analysis of allele transmission and segregation distortion in the F2 pedigree.** Alleles inherited from the Corsican grandparent are highlighted in green. Click here for file

Additional file 6 Summary of polymorphic and mapped markers on map 1 and map 3 for the G2F, G2M and F2 linkage maps. Click here for file

Additional file 7 Map length and number of markers for the three maps: G2F, G2M and F2. Click here for file

Additional file 8 **Result of the Wilcoxon signed rank test** (***P*-values) of pair-wise recombination for markers common to F2 and G2F and to F2 and G2M.** Click here for file

Additional file 9 **Distribution of the observed number of markers relative to the expected number of markers, assuming a Poisson distribution for the F2, G2F and G2M parental trees.** Blocks with the same marker counts were summed and the resulting frequencies compared with the expected frequencies generated from the Poisson distribution function (indeed, if recombination on each chromosome were completely random, a Poisson distribution with a variance equal to the mean would be expected, as suggest by Haldane (1931). Haldane, J. B. S. (1931) The cytological basis of genetical interference. *Cytologia* 3:54–65). Black squares indicate the lower and upper thresholds (in terms of the number of markers per block) defining hotspots and coldspots of recombination, respectively. Click here for file

Additional file 10 **This supplementary data file contains three items.** *A representation of marker density in the linkage groups of the G2F, G2M and F2 maps, highlighting coldspots and hotspots of recombination. Marker density was determined by shifting an interval along the map in 1 cM increments. The horizontal lines indicate the lower and upper thresholds defining gene clusters and gaps, respectively. x-axis: map distance for the whole linkage group (marker position as in Additional file 3, common markers are highlighted in green (between G2F and F2) and in pink (between G2M and F2), and markers common to G2F, G2M and F2 are enclosed in a box. y-axis: number of genes in the interval. Clusters common to the F2 map and at least one G2 map are indicated by orange circles connected by dotted orange lines. Clusters common to the G2F and G2M maps are indicated by black circles connected by dotted black lines. Clusters observed on only one map are indicated by black circles. *A table indicating the number of recombination hot- and coldspots on the G2F, G2M and F2 linkage maps. *A Venn diagram based on the table, to visualize the number of cold- (in black) and hotspots (in red) specific to a given map or common to different maps. Click here for file

Additional file 11 Distribution of the map distance between two adjacent mapped markers for the three maps (G2F, G2M, F2). x-axis: distance between the markers, y-axis: number of intervals. Click here for file

Additional file 12 **Polymorphism rate (last column) estimated from *in silico *screening of aligned 454 reads for the three mapped genotypes (10.159.3, G2M map; 9.106.3, G2F map; and H12, F2 map).** Click here for file

Additional file 13 Pinus pinaster pedigrees used for linkage mapping. Click here for file

Additional file 14 **Overview of the EST datasets used to construct PineContig_v2. The accession ID of 454 data is as for the Sequence Read Archive of the NCBI database **[82]**.** Click here for file

Additional file 15 PineContig_v2 libraries produced with Aquitaine genotypes of maritime pine. Click here for file

Additional file 16 Distribution of the 584,089 cleaned reads obtained from Aquitaine genotypes. Click here for file

Additional file 17 Summary of SNPs included in the 12 k bead type Infinium assay. Click here for file

Additional file 18 **Information obtained with MassArray assay design 4.1 software (Sequenom).** Two multiplexes (W1 with 22 SNPs and W2 with 12 SNPs) were developed. Click here for file

Additional file 19 Summary of the various types of markers combined with the 12 k-SNP markers for the construction of the G2 (A) and F2 (B) linkage maps. Click here for file

Additional file 20 Bandwidth values obtained from Kernel density analysis for the F2, G2F and G2M linkage maps. Click here for file
