# Supplementary material for: High-density linkage mapping in a pine tree reveals a genomic region associated with inbreeding depression and provides clues to the extent and distribution of meiotic recombination
Source: BMC Biol. 2013 Apr 18;11:50. doi: 10.1186/1741-7007-11-50 (PMC3660193; doi:10.1186/1741-7007-11-50)
Supplement: Additional file 1 — Occurrence of loci displaying inconsistent failure in the two mapping populations studied (F2 and G2): A, B, C, D, polymorphic vs. failed; E, F, G, H monomorphic vs. failed (see illustration in Figure 1). [file 1741-7007-11-50-S1.doc]

**Additional file 1**: Occurrence of loci displaying inconsistent failure in the two mapping populations studied (F2 and G2): A, B, C, D, polymorphic *vs*. failed; E, F, G, H monomorphic vs. failed (see illustration in Figure 1).

|  | A | B | C | D | Total |
| --- | --- | --- | --- | --- | --- |
| Polymorphic G2/Failed F2 | 139  (76.8%) | 26  (14.4%) | 16  (8.8%) | 0  (0%) | 181 |
| Polymorphic F2/Failed G2 | 18  (32.1%) | 14  (25%) | 21  (37.5%) | 3  (5.4%) | 56 |

|  | E | F | G | H | Total |
| --- | --- | --- | --- | --- | --- |
| Monomorphic G2/Failed F2 | 155  (69.8%) | 66  (29.7%) | 0  (0%) | 1  (0.5%) | 222 |
| Monomorphic F2/Failed G2 | 203  (88.3%) | 26  (11.3%) | 1  (0.4%) | 0  (0%) | 230 |
