# Supplementary material for: High-density linkage mapping in a pine tree reveals a genomic region associated with inbreeding depression and provides clues to the extent and distribution of meiotic recombination
Source: BMC Biol. 2013 Apr 18;11:50. doi: 10.1186/1741-7007-11-50 (PMC3660193; doi:10.1186/1741-7007-11-50)
Supplement: Additional file 3 — Genetic linkage maps obtained from segregation data for the G2 and F2 mapping populations. Markers common to the female (G2F), male (G2M) and F2 maps are linked by green dashed lines. Framework markers (segregating in a 1:1 ratio) are indicated in black, whereas accessory markers (segregating in a 1:2:1 ratio) are indicated in blue, followed, in brackets, by the distance (in cM) to the nearest framework marker and the corresponding LOD score. Markers displaying segregation distortion in the initial dataset are indicated with an asterisk (*). [file 1741-7007-11-50-S3.ppt]

## Slide 1
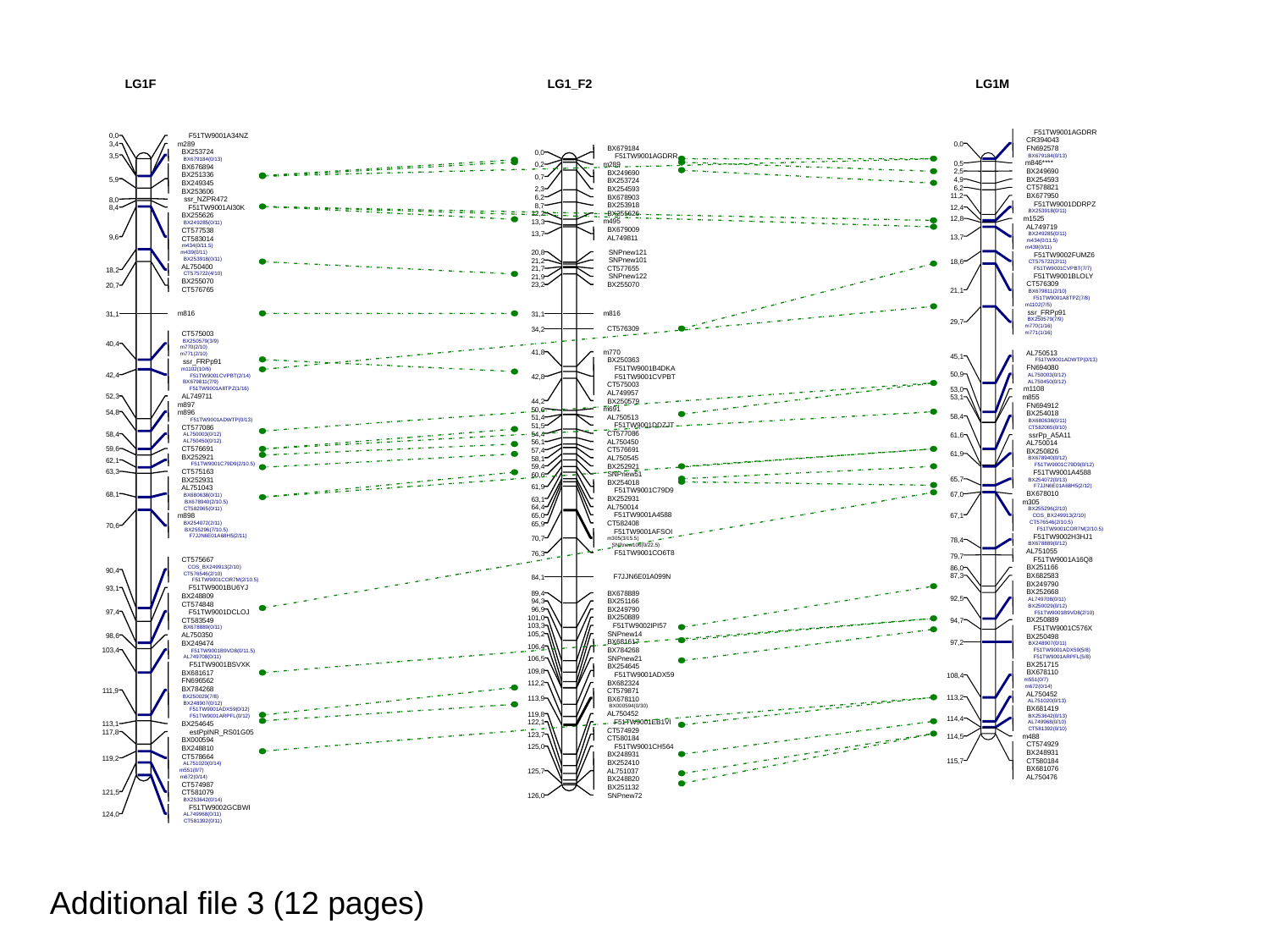

LG1F
F51TW9001A34NZ
0,0
m289
3,4
BX253724
3,5
BX679184(0/13)
BX676894
BX251336
5,9
BX249345
BX253606
ssr_NZPR472
8,0
F51TW9001AI30K
8,4
BX255626
BX249285(0/11)
CT577538
9,6
CT583014
m434(0/11.5)
m439(0/11)
BX253918(0/11)
AL750400
18,2
CT575722(4/10)
BX255070
20,7
CT576765
m816
31,1
CT575003
BX250579(3/9)
40,4
m770(2/10)
m771(2/10)
ssr_FRPp91
m1102(10/6)
42,4
F51TW9001CVPBT(2/14)
BX679811(7/9)
F51TW9001A8TPZ(1/16)
AL749711
52,3
m897
54,8
m896
F51TW9001ADWTP(0/13)
CT577086
58,4
AL750003(0/12)
AL750450(0/12)
CT576691
59,6
BX252921
62,1
F51TW9001C79D9(2/10.5)
CT575163
63,3
BX252931
AL751043
68,1
BX680638(0/11)
BX678940(2/10.5)
CT582065(0/11)
m898
BX254072(2/11)
70,6
BX255296(7/10.5)
F7JJN6E01A68H5(2/11)
CT575667
COS_BX249913(2/10)
90,4
CT576546(2/10)
F51TW9001COR7M(2/10.5)
F51TW9001BU6YJ
93,1
BX248809
CT574848
97,4
F51TW9001DCLOJ
CT583549
BX678889(0/11)
AL750350
98,6
BX249474
103,4
F51TW9001B9VD8(0/11.5)
AL749708(0/11)
F51TW9001BSVXK
BX681617
FN696562
BX784268
111,9
BX250029(7/8)
BX248907(0/12)
F51TW9001ADX59(0/12)
F51TW9001ARPFL(0/12)
BX254645
113,1
estPpINR_RS01G05
117,8
BX000594
BX248810
CT578664
119,2
AL751020(0/14)
m551(0/7)
m672(0/14)
CT574987
121,5
CT581079
BX253642(0/14)
F51TW9002GCBWI
124,0
AL749968(0/11)
CT581392(0/11)
LG1_F2
BX679184
0,0
F51TW9001AGDRR
m289
0,2
BX249690
0,7
BX253724
BX254593
2,3
BX678903
6,2
BX253918
8,7
BX255626
12,2
m495
13,3
BX679009
13,7
AL749811
SNPnew121
20,8
SNPnew101
21,2
CT577655
21,7
SNPnew122
21,9
BX255070
23,2
m816
31,1
CT576309
34,2
m770
41,8
BX250363
F51TW9001B4DKA
F51TW9001CVPBT
42,8
CT575003
AL749957
BX250579
44,2
m691
50,6
AL750513
51,4
F51TW9001DDZJT
51,5
CT577086
54,4
AL750450
56,1
CT576691
57,4
AL750545
58,1
BX252921
59,4
SNPnew51
60,6
BX254018
61,9
F51TW9001C79D9
BX252931
63,1
AL750014
64,4
F51TW9001A4588
65,0
CT582408
65,9
F51TW9001AFSOI
70,7
m305(3/15.5)
SNPnew106(0/22.5)
F51TW9001CO6T8
76,3
F7JJN6E01A099N
84,1
BX678889
89,4
BX251166
94,3
BX249790
96,9
BX250889
101,0
F51TW9002IPI57
103,3
SNPnew14
105,2
BX681617
106,4
BX784268
SNPnew21
106,5
BX254645
109,8
F51TW9001ADX59
BX682324
112,2
CT579871
113,9
BX678110
BX000594(0/30)
AL750452
119,8
F51TW9001EB1VI
122,1
CT574929
123,7
CT580184
F51TW9001CH564
125,0
BX248931
BX252410
AL751037
125,7
BX248820
BX251132
SNPnew72
126,0
LG1M
F51TW9001AGDRR
CR394043
0,0
FN692578
BX679184(0/13)
m846****
0,5
BX249690
2,5
BX254593
4,9
CT578821
6,2
BX677950
11,2
F51TW9001DDRPZ
12,4
BX253918(0/11)
m1525
12,8
AL749719
BX249285(0/11)
13,7
m434(0/11.5)
m439(0/11)
F51TW9002FUMZ6
18,6
CT575722(2/11)
F51TW9001CVPBT(7/7)
F51TW9001BLOLY
CT576309
21,1
BX679811(2/10)
F51TW9001A8TPZ(7/8)
m1102(7/5)
ssr_FRPp91
BX250579(7/9)
29,7
m770(1/16)
m771(1/16)
AL750513
45,1
F51TW9001ADWTP(0/13)
FN694080
50,9
AL750003(0/12)
AL750450(0/12)
m1108
53,0
m855
53,1
FN694912
BX254018
58,4
BX680638(0/11)
CT582065(0/10)
ssrPp_A5A11
61,6
AL750014
BX250826
61,9
BX678940(0/12)
F51TW9001C79D9(0/12)
F51TW9001A4588
65,7
BX254072(0/13)
F7JJN6E01A68H5(2/12)
BX678010
67,0
m305
BX255296(2/10)
67,1
COS_BX249913(2/10)
CT576546(2/10.5)
F51TW9001COR7M(2/10.5)
F51TW9002H3HJ1
78,4
BX678889(0/12)
AL751055
79,7
F51TW9001A16Q8
BX251166
86,0
BX682583
87,3
BX249790
BX252668
92,5
AL749708(0/11)
BX250029(0/12)
F51TW9001B9VD8(2/10)
BX250889
94,7
F51TW9001C576X
BX250498
97,2
BX248907(0/11)
F51TW9001ADX59(5/8)
F51TW9001ARPFL(5/8)
BX251715
BX678110
108,4
m551(0/7)
m672(0/14)
AL750452
113,2
AL751020(0/13)
BX681419
BX253642(0/13)
114,4
AL749968(0/10)
CT581392(0/10)
m488
114,5
CT574929
BX248931
CT580184
115,7
BX681076
AL750476
Additional file 3 (12 pages)

## Slide 2
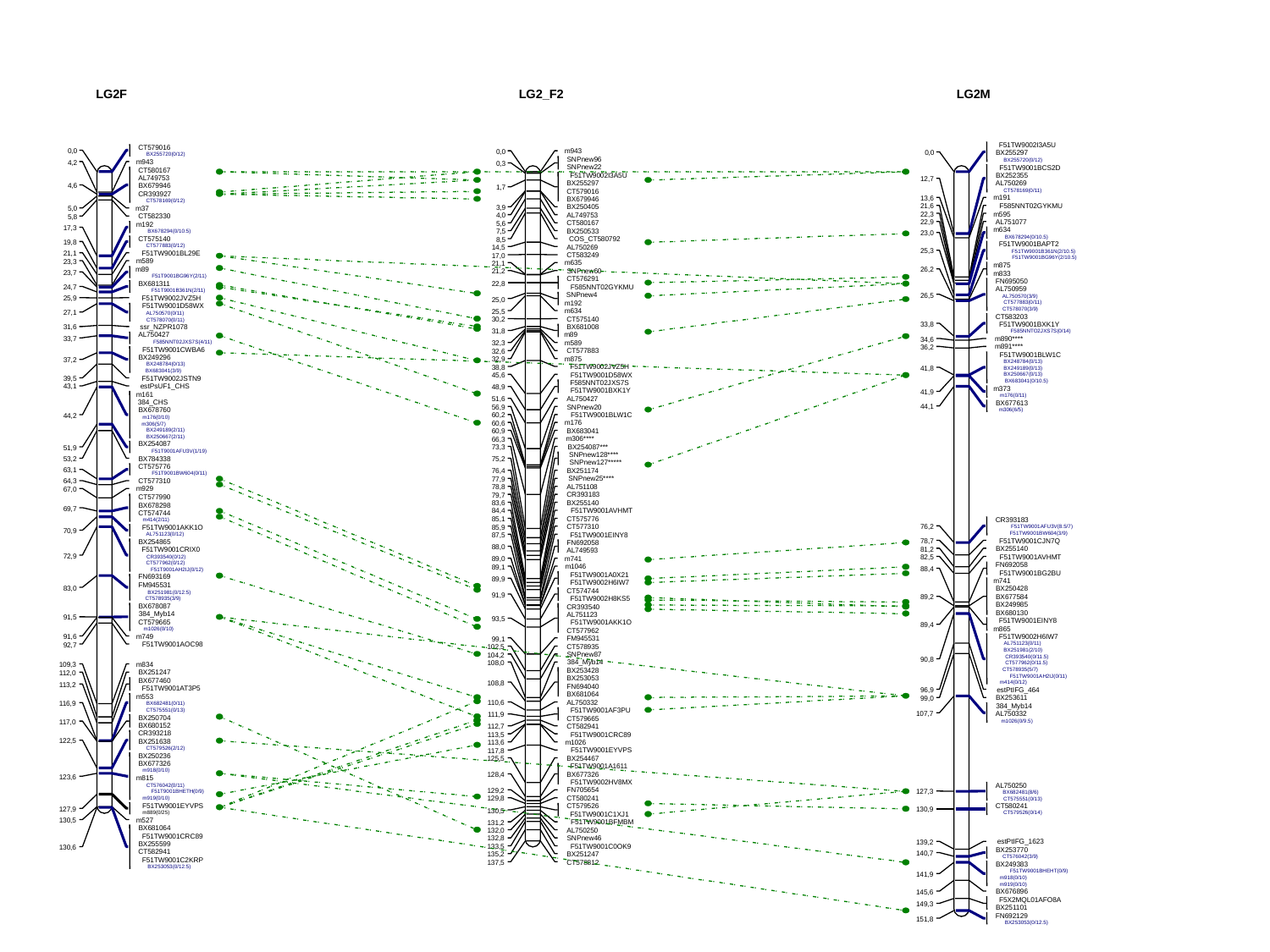

LG2F
CT579016
0,0
BX255720(0/12)
m943
4,2
CT580167
AL749753
4,6
BX679946
CR393927
CT578169(0/12)
m37
5,0
CT582330
5,8
m192
17,3
BX678294(0/10.5)
CT575140
19,8
CT577883(0/12)
F51TW9001BL29E
21,1
m589
23,3
m89
23,7
F51T9001BG96Y(2/11)
BX681311
24,7
F51T9001B361N(2/11)
F51TW9002JVZ5H
25,9
F51TW9001D58WX
27,1
AL750570(0/11)
CT578070(0/11)
ssr_NZPR1078
31,6
AL750427
33,7
F585NNT02JXS7S(4/11)
F51TW9001CWBA6
BX249296
37,2
BX248784(0/13)
BX683041(3/9)
F51TW9002JSTN9
39,5
estPsUF1_CHS
43,1
m161
384_CHS
BX678760
44,2
m176(0/10)
m306(5/7)
BX249189(2/11)
BX250667(2/11)
BX254087
51,9
F51T9001AFU3V(1/19)
BX784338
53,2
CT575776
63,1
F51T9001BW604(0/11)
CT577310
64,3
m929
67,0
CT577990
BX678298
69,7
CT574744
m414(2/11)
F51TW9001AKK1O
70,9
AL751123(0/12)
BX254865
F51TW9001CRIX0
72,9
CR393540(0/12)
CT577962(0/12)
F51T9001AH2IJ(0/12)
FN693169
FM945531
83,0
BX251981(0/12.5)
CT578935(3/9)
BX678087
384_Myb14
91,5
CT579665
m1026(0/10)
m749
91,6
F51TW9001AOC98
92,7
m834
109,3
BX251247
112,0
BX677460
113,2
F51TW9001AT3P5
m553
116,9
BX682481(0/11)
CT575551(0/13)
BX250704
117,0
BX680152
CR393218
122,5
BX251638
CT579526(2/12)
BX250236
BX677326
m918(0/10)
123,6
m815
CT576042(0/11)
F51T9001BHETH(0/9)
m919(0/10)
F51TW9001EYVPS
127,9
m889(0/25)
m527
130,5
BX681064
F51TW9001CRC89
BX255599
130,6
CT582941
F51TW9001C2KRP
BX253053(0/12.5)
LG2_F2
m943
0,0
SNPnew96
0,3
SNPnew22
F51TW9002I3A5U
BX255297
1,7
CT579016
BX679946
BX250405
3,9
AL749753
4,0
CT580167
5,6
BX250533
7,5
COS_CT580792
8,5
AL750269
14,5
CT583249
17,0
m635
21,1
SNPnew60
21,2
CT576291
22,8
F585NNT02GYKMU
SNPnew4
25,0
m192
m634
25,5
CT575140
30,2
BX681008
31,8
m89
m589
32,3
CT577883
32,6
m875
32,9
F51TW9002JVZ5H
38,8
F51TW9001D58WX
45,6
F585NNT02JXS7S
48,9
F51TW9001BXK1Y
AL750427
51,6
SNPnew20
56,9
F51TW9001BLW1C
60,2
m176
60,6
BX683041
60,9
m306****
66,3
BX254087***
73,3
SNPnew128****
75,2
SNPnew127*****
BX251174
76,4
SNPnew25****
77,9
AL751108
78,8
CR393183
79,7
BX255140
83,6
F51TW9001AVHMT
84,4
CT575776
85,1
CT577310
85,9
F51TW9001EINY8
87,5
FN692058
88,0
AL749593
m741
89,0
m1046
89,1
F51TW9001A0X21
89,9
F51TW9002H6IW7
CT574744
91,9
F51TW9002H8KS5
CR393540
AL751123
93,5
F51TW9001AKK1O
CT577962
FM945531
99,1
CT578935
102,5
SNPnew87
104,2
384_Myb14
108,0
BX253428
BX253053
108,8
FN694040
BX681064
AL750332
110,6
F51TW9001AF3PU
111,9
CT579665
CT582941
112,7
F51TW9001CRC89
113,5
m1026
113,6
F51TW9001EYVPS
117,8
BX254467
125,5
F51TW9001A1611
BX677326
128,4
F51TW9002HV8MX
FN705654
129,2
CT580241
129,8
CT579526
130,5
F51TW9001C1XJ1
F51TW9001BFMBM
131,2
AL750250
132,0
SNPnew46
132,8
F51TW9001C0OK9
133,5
BX251247
135,2
CT578812
137,5
LG2M
F51TW9002I3A5U
0,0
BX255297
BX255720(0/12)
F51TW9001BCS2D
BX252355
12,7
AL750269
CT578169(0/11)
m191
13,6
F585NNT02GYKMU
21,6
m595
22,3
AL751077
22,9
m634
23,0
BX678294(0/10.5)
F51TW9001BAPT2
25,3
F51TW9001B361N(2/10.5)
F51TW9001BG96Y(2/10.5)
m875
26,2
m833
FN695050
AL750959
26,5
AL750570(3/9)
CT577883(0/11)
CT578070(3/9)
CT583203
33,8
F51TW9001BXK1Y
F585NNTO2JXS7S(0/14)
m890****
34,6
m891****
36,2
F51TW9001BLW1C
BX248784(0/13)
41,8
BX249189(0/13)
BX250667(0/13)
BX683041(0/10.5)
m373
41,9
m176(0/11)
BX677613
44,1
m306(6/5)
CR393183
76,2
F51TW9001AFU3V(8.5/7)
F51TW9001BW604(3/9)
F51TW9001CJN7Q
78,7
BX255140
81,2
F51TW9001AVHMT
82,5
FN692058
88,4
F51TW9001BG2BU
m741
BX250428
BX677584
89,2
BX249985
BX680130
F51TW9001EINY8
89,4
m865
F51TW9002H6IW7
AL751123(0/11)
BX251981(2/10)
CR393540(0/11.5)
90,8
CT577962(0/11.5)
CT578935(5/7)
F51TW9001AH2IJ(0/11)
m414(0/12)
estPtIFG_464
96,9
BX253611
99,0
384_Myb14
107,7
AL750332
m1026(0/9.5)
AL750250
127,3
BX682481(8/6)
CT575551(0/13)
CT580241
130,9
CT579526(0/14)
estPtIFG_1623
139,2
BX253770
140,7
CT576042(3/9)
BX249383
F51TW9001BHEHT(0/9)
141,9
m918(0/10)
m919(0/10)
BX676896
145,6
F5X2MQL01AFO8A
BX251101
149,3
FN692129
151,8
BX253053(0/12.5)

## Slide 3
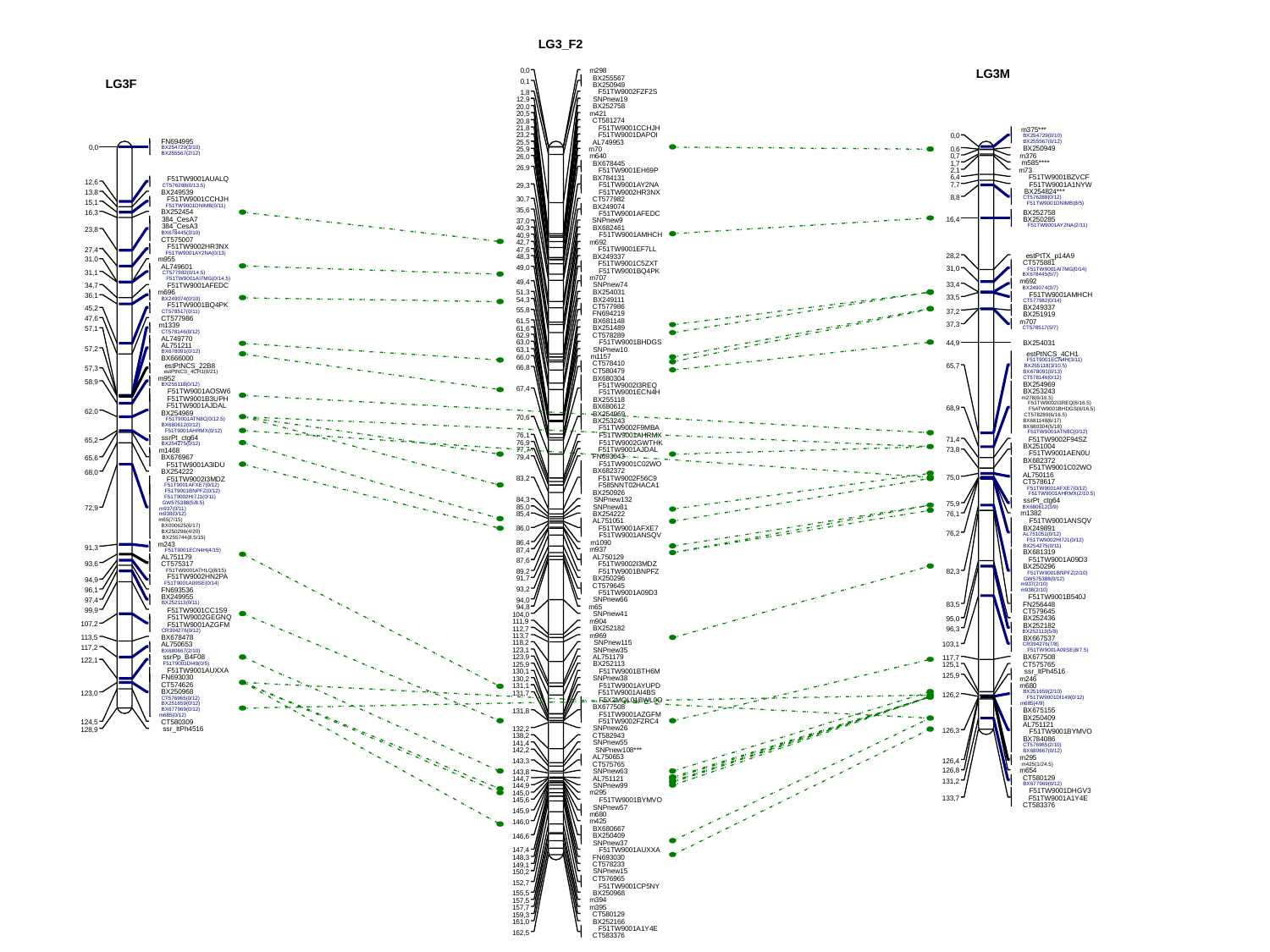

LG3_F2
m298
0,0
BX255567
0,1
BX250949
F51TW9002FZF2S
1,8
SNPnew19
12,9
BX252758
20,0
m421
20,5
CT581274
20,8
F51TW9001CCHJH
21,8
F51TW9001DAPOI
23,2
AL749953
25,5
m70
25,9
m640
26,0
BX678445
26,9
F51TW9001EH69P
BX784131
F51TW9001AY2NA
29,3
F51TW9002HR3NX
CT577982
30,7
BX249074
35,6
F51TW9001AFEDC
SNPnew9
37,0
BX682461
40,3
F51TW9001AMHCH
40,9
m692
42,7
F51TW9001EF7LL
47,6
BX249337
48,3
F51TW9001C5ZXT
49,0
F51TW9001BQ4PK
m707
49,4
SNPnew74
BX254031
51,3
BX249111
54,3
CT577986
55,8
FN694219
BX681148
61,5
BX251489
61,6
CT578289
62,9
F51TW9001BHDGS
63,0
SNPnew10
63,1
m1157
66,0
CT578410
66,8
CT580479
BX680304
F51TW9002I3REQ
67,4
F51TW9001ECN4H
BX255118
BX680612
BX254969
70,6
BX253243
F51TW9002F9MBA
F51TW9001AHRMX
76,1
F51TW9002GWTHK
76,9
F51TW9001AJDAL
77,7
FN693043
79,4
F51TW9001C02WO
BX682372
F51TW9002F56C9
83,2
F585NNT02HACA1
BX250926
SNPnew132
84,3
SNPnew81
85,0
BX254222
85,4
AL751051
F51TW9001AFXE7
86,0
F51TW9001ANSQV
m1090
86,4
m937
87,4
AL750129
87,6
F51TW9002I3MDZ
F51TW9001BNPFZ
89,2
BX250296
91,7
CT579645
93,2
F51TW9001A09D3
SNPnew66
94,0
m65
94,8
SNPnew41
104,0
m904
111,9
BX252182
112,7
m969
113,7
SNPnew115
118,2
SNPnew35
123,1
AL751179
123,9
BX252113
125,9
F51TW9001BTH6M
130,1
SNPnew38
130,2
F51TW9001AYUPD
131,1
F51TW9001AI4BS
131,7
F5X2MQL01BWL0O
BX677508
131,8
F51TW9001AZGFM
F51TW9002FZRC4
SNPnew26
132,2
CT582943
138,2
SNPnew55
141,4
SNPnew108***
142,2
AL750653
143,3
CT575765
SNPnew63
143,8
AL751121
144,7
SNPnew99
144,9
m295
145,0
F51TW9001BYMVO
145,6
SNPnew57
145,9
m680
m425
146,0
BX680667
BX250409
146,6
SNPnew37
F51TW9001AUXXA
147,4
FN693030
148,3
CT578233
149,1
SNPnew15
150,2
CT576965
152,7
F51TW9001CP5NY
BX250968
155,5
m394
157,5
m395
157,7
CT580129
159,3
BX252166
161,0
F51TW9001A1Y4E
162,5
CT583376
LG3M
m375***
0,0
BX254729(0/10)
BX255567(0/12)
BX250949
0,6
m376
0,7
m585****
1,7
m73
2,1
F51TW9001BZVCF
6,4
F51TW9001A1NYW
7,7
BX254824***
8,8
CT576288(0/12)
F51TW9001DNIMB(8/5)
BX252758
16,4
BX250285
F51TW9001AY2NA(2/11)
estPtTX_p14A9
28,2
CT575881
31,0
F51TW9001AI7MG(0/14)
BX678445(5/7)
m692
33,4
BX249074(3/7)
F51TW9001AMHCH
33,5
CT577982(0/14)
BX249337
37,2
BX251919
m707
37,3
CT578517(5/7)
BX254031
44,9
estPtNCS_4CH1
F51T9001ECN4H(3/11)
65,7
BX255118(3/10.5)
BX678091(0/13)
CT578146(0/12)
BX254969
BX253243
m278(6/16.5)
F51TW9002I3REQ(6/16.5)
68,9
F5ATW9001BHDGS(6/16.5)
CT578289(6/16.5)
BX681148(6/17)
BX680304(5/18)
F51TW9001ATN8C(0/12)
F51TW9002F94SZ
71,4
BX251004
73,8
F51TW9001AEN0U
BX682372
F51TW9001C02WO
AL750116
75,0
CT578617
F51TW9001AFXE7(0/12)
F51TW9001AHRMX(2/10.5)
ssrPt_ctg64
75,9
BX680612(3/9)
m1382
76,1
F51TW9001ANSQV
BX249891
76,2
AL751051(0/12)
F51TW9002HI7J1(0/12)
BX254275(0/11)
BX681319
F51TW9001A09D3
BX250296
82,3
F51TW9001BNPFZ(2/10)
GW575388(0/12)
m937(2/10)
m938(2/10)
F51TW9001B540J
FN256448
83,5
CT579645
BX252436
95,0
BX252182
96,3
BX252113(5/8)
BX667537
103,1
CR394276(7/8)
F51TW9001A09SE(8/7.5)
BX677508
117,7
CT575765
125,1
ssr_ItPh4516
125,9
m246
m680
BX251659(2/10)
126,2
F51TW9001DI149(0/12)
m685(4/9)
BX675155
BX250409
AL751121
126,3
F51TW9001BYMVO
BX784086
CT576965(2/10)
BX680667(0/12)
m295
126,4
m425(1/24.5)
m654
126,8
CT580129
131,2
BX677969(0/12)
F51TW9001DHGV3
F51TW9001A1Y4E
133,7
CT583376
LG3F
FN694995
0,0
BX254729(3/10)
BX255567(2/12)
F51TW9001AUALQ
12,6
CT576288(0/13.5)
BX249539
13,8
F51TW9001CCHJH
15,1
F51TW9001DNIMB(0/11)
BX252454
16,3
384_CesA7
384_CesA3
23,8
BX678445(3/10)
CT575007
F51TW9002HR3NX
27,4
F51TW9001AY2NA(0/13)
m955
31,0
AL749601
31,1
CT577982(0/14.5)
F51TW9001AI7MG(0/14.5)
F51TW9001AFEDC
34,7
m696
36,1
BX249074(0/10)
F51TW9001BQ4PK
45,2
CT578517(0/11)
CT577986
47,6
m1339
57,1
CT578146(0/12)
AL749770
AL751211
57,2
BX678091(0/12)
BX666000
estPtNCS_22B8
57,3
estPtNCS_4CH1(0/21)
m952
58,9
BX255118(0/12)
F51TW9001AOSW6
F51TW9001B3UPH
F51TW9001AJDAL
62,0
BX254969
F51T9001ATN8C(0/12.5)
BX680612(0/12)
F51T9001AHRMX(0/12)
ssrPt_ctg64
65,2
BX254275(0/12)
m1468
BX676967
65,6
F51TW9001A3IDU
BX254222
68,0
F51TW9002I3MDZ
F51T9001AFXE7(0/12)
F51T9001BNPFZ(0/12)
F51T9002HI7J1(0/11)
GW575388(5/8.5)
72,9
m937(0/11)
m938(0/12)
m65(7/15)
BX000625(6/17)
BX250296(4/20)
BX255744(8.5/15)
m243
91,3
F51T9001ECN4H(4/15)
AL751179
93,6
CT575317
F51TW9001ATHLQ(8/15)
F51TW9002HN2PA
94,9
F51T9001A09SE(0/14)
FN693536
96,1
BX249955
97,4
BX252113(0/11)
F51TW9001CC1S9
99,9
F51TW9002GEGNQ
107,2
F51TW9001AZGFM
CR394276(0/12)
BX678478
113,5
AL750653
117,2
BX680667(2/10)
ssrPp_B4F08
122,1
F51T9001DI49(0/5)
F51TW9001AUXXA
FN693030
CT574626
BX250968
123,0
CT576965(0/12)
BX251659(0/12)
BX677969(0/12)
m685(0/12)
CT580309
124,5
ssr_ItPh4516
128,9

## Slide 4
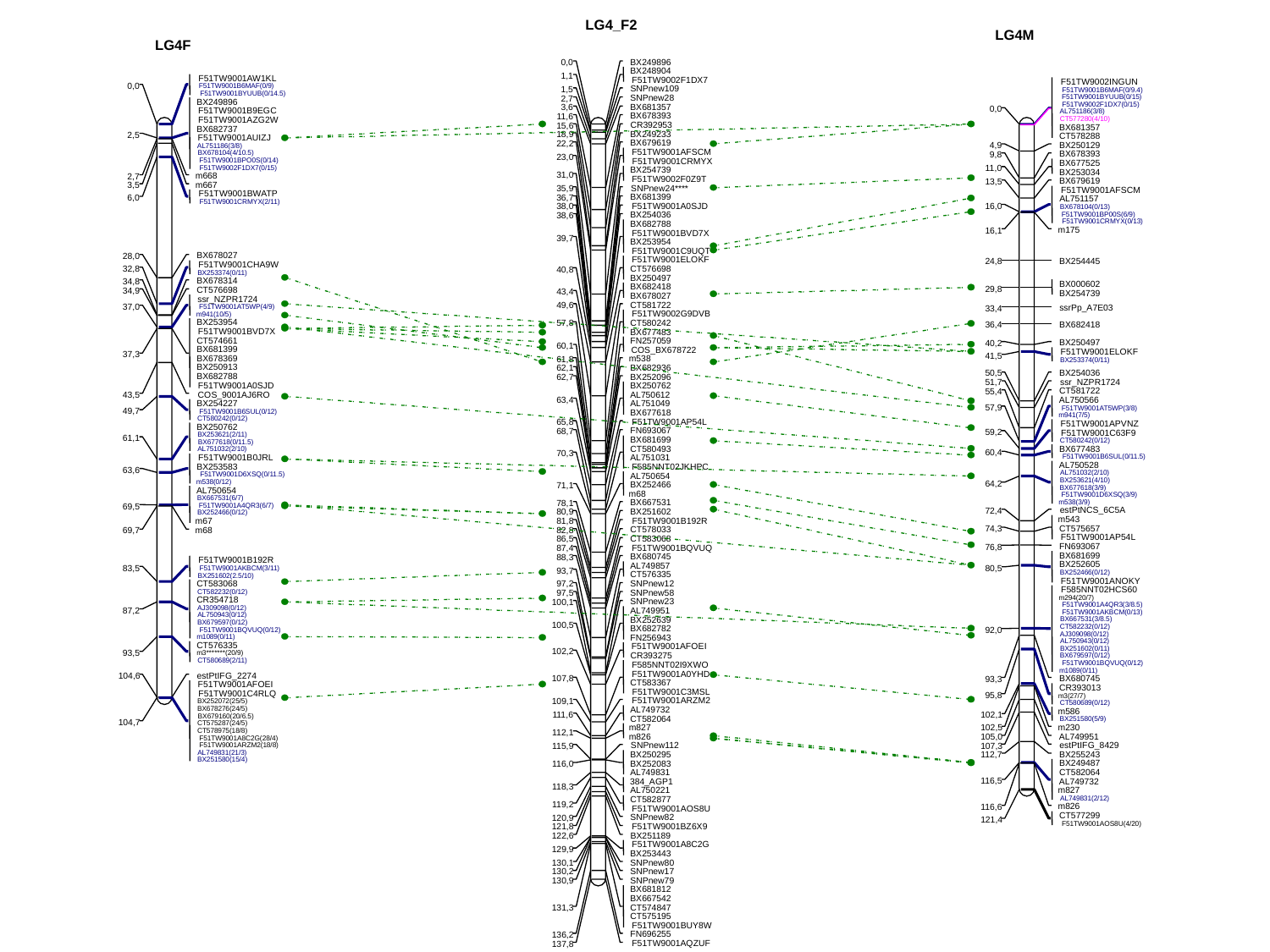

LG4_F2
BX249896
0,0
BX248904
1,1
F51TW9002F1DX7
SNPnew109
1,5
SNPnew28
2,7
BX681357
3,6
BX678393
11,6
CR392953
15,6
BX249233
18,9
BX679619
22,2
F51TW9001AFSCM
23,0
F51TW9001CRMYX
BX254739
31,0
F51TW9002F0Z9T
SNPnew24****
35,9
BX681399
36,7
F51TW9001A0SJD
38,0
BX254036
38,6
BX682788
F51TW9001BVD7X
39,7
BX253954
F51TW9001C9UQT
F51TW9001ELOKF
CT576698
40,8
BX250497
BX682418
43,4
BX678027
CT581722
49,6
F51TW9002G9DVB
CT580242
57,8
BX677483
FN257059
60,1
COS_BX678722
m538
61,8
BX682936
62,1
BX252096
62,7
BX250762
AL750612
63,4
AL751049
BX677618
F51TW9001AP54L
65,8
FN693067
68,7
BX681699
CT580493
70,3
AL751031
F585NNT02JKHPC
AL750654
BX252466
71,1
m68
BX667531
78,1
BX251602
80,9
F51TW9001B192R
81,8
CT578033
82,8
CT583068
86,5
F51TW9001BQVUQ
87,4
BX680745
88,3
AL749857
93,7
CT576335
SNPnew12
97,2
SNPnew58
97,5
SNPnew23
100,1
AL749951
BX252639
100,5
BX682782
FN256943
F51TW9001AFOEI
102,2
CR393275
F585NNT02I9XWO
F51TW9001A0YHD
107,8
CT583367
F51TW9001C3MSL
F51TW9001ARZM2
109,1
AL749732
111,6
CT582064
m827
112,1
m826
SNPnew112
115,9
BX250295
BX252083
116,0
AL749831
384_AGP1
118,3
AL750221
CT582877
119,2
F51TW9001AOS8U
SNPnew82
120,9
F51TW9001BZ6X9
121,8
BX251189
122,6
F51TW9001A8C2G
129,9
BX253443
SNPnew80
130,1
SNPnew17
130,2
SNPnew79
130,9
BX681812
BX667542
CT574847
131,3
CT575195
F51TW9001BUY8W
FN696255
136,2
F51TW9001AQZUF
137,8
LG4M
F51TW9002INGUN
F51TW9001B6MAF(0/9.4)
F51TW9001BYUUB(0/15)
F51TW9002F1DX7(0/15)
0,0
AL751186(3/8)
CT577280(4/10)
BX681357
CT578288
BX250129
4,9
BX678393
9,8
BX677525
11,0
BX253034
BX679619
13,5
F51TW9001AFSCM
AL751157
16,0
BX678104(0/13)
F51TW9001BP00S(6/9)
F51TW9001CRMYX(0/13)
m175
16,1
BX254445
24,8
BX000602
29,8
BX254739
ssrPp_A7E03
33,4
BX682418
36,4
BX250497
40,2
F51TW9001ELOKF
41,5
BX253374(0/11)
BX254036
50,5
ssr_NZPR1724
51,7
CT581722
55,4
AL750566
57,9
F51TW9001AT5WP(3/8)
m941(7/5)
F51TW9001APVNZ
59,2
F51TW9001C63F9
CT580242(0/12)
BX677483
60,4
F51TW9001B6SUL(0/11.5)
AL750528
AL751032(2/10)
BX253621(4/10)
64,2
BX677618(3/9)
F51TW9001D6XSQ(3/9)
m538(3/9)
estPtNCS_6C5A
72,4
m543
CT575657
74,3
F51TW9001AP54L
FN693067
76,8
BX681699
BX252605
80,5
BX252466(0/12)
F51TW9001ANOKY
F585NNT02HCS60
m294(20/7)
F51TW9001A4QR3(3/8.5)
F51TW9001AKBCM(0/13)
BX667531(3/8.5)
CT582232(0/12)
92,0
AJ309098(0/12)
AL750943(0/12)
BX251602(0/11)
BX679597(0/12)
F51TW9001BQVUQ(0/12)
m1089(0/11)
BX680745
93,3
CR393013
95,8
m3(27/7)
CT580689(0/12)
m586
102,1
BX251580(5/9)
m230
102,5
AL749951
105,0
estPtIFG_8429
107,3
BX255243
112,7
BX249487
CT582064
116,5
AL749732
m827
AL749831(2/12)
m826
116,6
CT577299
121,4
F51TW9001AOS8U(4/20)
LG4F
F51TW9001AW1KL
0,0
F51TW9001B6MAF(0/9)
F51TW9001BYUUB(0/14.5)
BX249896
F51TW9001B9EGC
F51TW9001AZG2W
BX682737
2,5
F51TW9001AUIZJ
AL751186(3/8)
BX678104(4/10.5)
F51TW9001BPO0S(0/14)
F51TW9002F1DX7(0/15)
m668
2,7
m667
3,5
F51TW9001BWATP
6,0
F51TW9001CRMYX(2/11)
BX678027
28,0
F51TW9001CHA9W
32,8
BX253374(0/11)
BX678314
34,8
CT576698
34,9
ssr_NZPR1724
37,0
F51TW9001AT5WP(4/9)
m941(10/5)
BX253954
F51TW9001BVD7X
CT574661
BX681399
37,3
BX678369
BX250913
BX682788
F51TW9001A0SJD
COS_9001AJ6RO
43,5
BX254227
49,7
F51TW9001B6SUL(0/12)
CT580242(0/12)
BX250762
BX253621(2/11)
61,1
BX677618(0/11.5)
AL751032(2/10)
F51TW9001B0JRL
BX253583
63,6
F51TW9001D6XSQ(0/11.5)
m538(0/12)
AL750654
BX667531(6/7)
69,5
F51TW9001A4QR3(6/7)
BX252466(0/12)
m67
m68
69,7
F51TW9001B192R
83,5
F51TW9001AKBCM(3/11)
BX251602(2.5/10)
CT583068
CT582232(0/12)
CR354718
AJ309098(0/12)
87,2
AL750943(0/12)
BX679597(0/12)
F51TW9001BQVUQ(0/12)
m1089(0/11)
CT576335
93,5
m3*******(20/9)
CT580689(2/11)
estPtIFG_2274
104,6
F51TW9001AFOEI
F51TW9001C4RLQ
BX252072(25/5)
BX678276(24/5)
BX679160(20/6.5)
104,7
CT575287(24/5)
CT578975(18/8)
F51TW9001A8C2G(28/4)
F51TW9001ARZM2(18/8)
AL749831(21/3)
BX251580(15/4)

## Slide 5
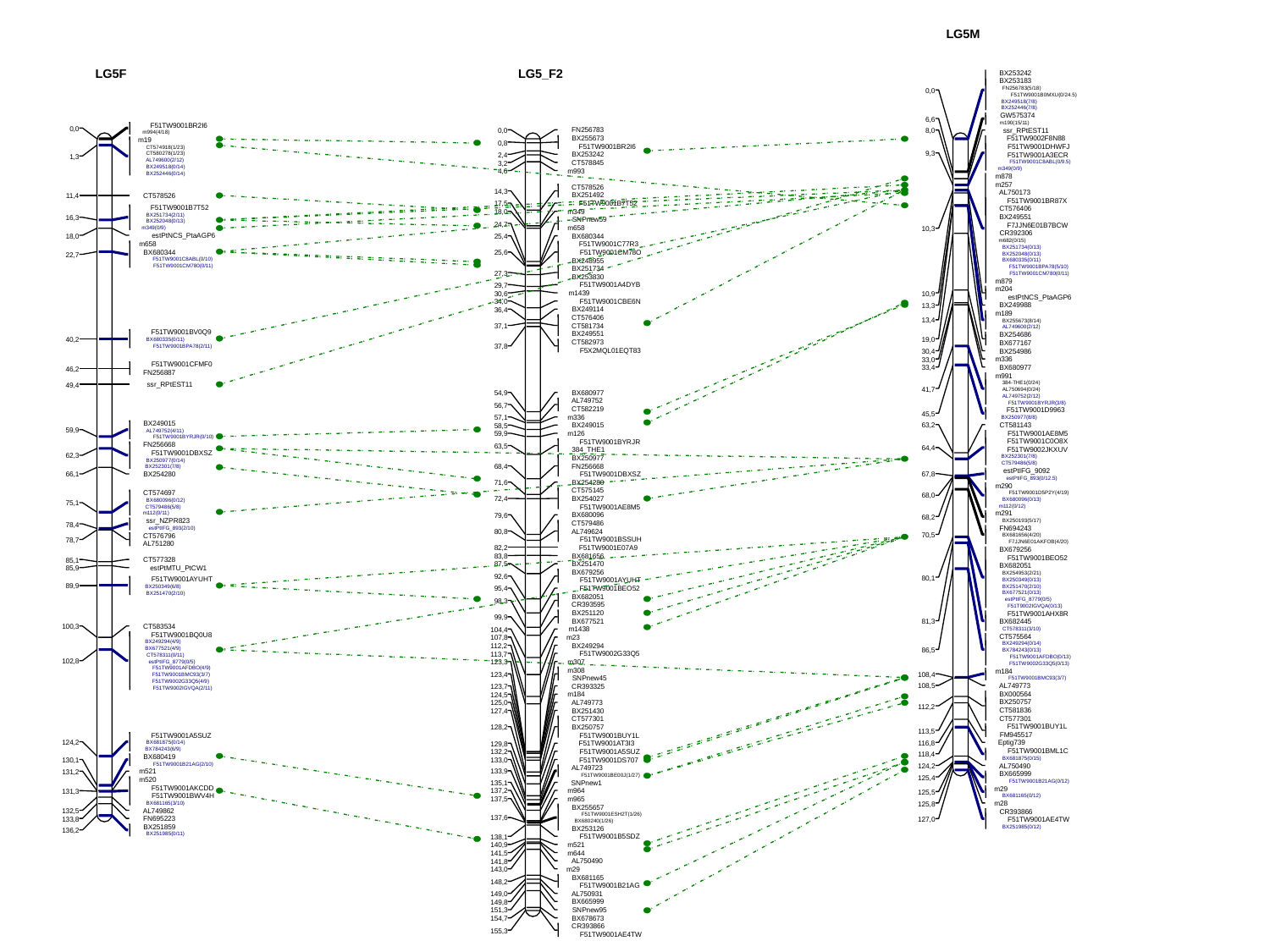

LG5M
BX253242
BX253183
FN256783(5/18)
0,0
F51TW9001B0MXU(0/24.5)
BX249518(7/8)
BX252446(7/8)
GW575374
6,6
m190(15/11)
ssr_RPtEST11
8,0
F51TW9002F8N88
F51TW9001DHWFJ
9,3
F51TW9001A3ECR
F51TW9001C8ABL(0/9.5)
m349(0/9)
m878
m257
AL750173
F51TW9001BR87X
CT576406
BX249551
F7JJN6E01B7BCW
10,3
CR392306
m682(0/15)
BX251734(0/13)
BX252048(0/13)
BX680335(0/11)
F51TW9001BPA78(5/10)
F51TW9001CM780(0/11)
m879
m204
10,9
estPtNCS_PtaAGP6
BX249988
13,3
m189
13,4
BX255673(8/14)
AL749600(2/12)
BX254686
19,0
BX677167
BX254986
30,4
m336
33,0
BX680977
33,4
m991
384-THE1(0/24)
41,7
AL750694(0/24)
AL749752(2/12)
F51TW9001BYRJR(3/8)
F51TW9001D9963
45,5
BX250977(8/8)
CT581143
63,2
F51TW9001AE8M5
F51TW9001C0O8X
64,4
F51TW9002JKXUV
BX252301(7/8)
CT579486(5/8)
estPtIFG_9092
67,8
estPtIFG_893(0/12.5)
m290
F51TW9001D5P2Y(4/19)
68,0
BX680096(0/13)
m112(0/12)
m291
68,2
BX250193(5/17)
FN694243
70,5
BX681656(4/20)
F7JJN6E01AKFOB(4/20)
BX679256
F51TW9001BEO52
BX682051
BX254953(2/21)
80,1
BX250349(0/13)
BX251470(2/10)
BX677521(0/13)
estPtIFG_8779(0/5)
F51T9002IGVQA(0/13)
F51TW9001AHX8R
81,3
BX682445
CT578311(3/10)
CT575564
BX249294(0/14)
86,5
BX784243(0/13)
F51TW9001AFDBO(0/13)
F51TW9002G33Q5(0/13)
m184
108,4
F51TW9001BMC93(3/7)
AL749773
108,5
BX000564
BX250757
112,2
CT581836
CT577301
F51TW9001BUY1L
113,5
FM945517
Eptig739
116,8
F51TW9001BML1C
118,4
BX681875(0/15)
AL750490
124,2
BX665999
125,4
F51TW9001B21AG(0/12)
m29
125,5
BX681165(0/12)
m28
125,8
CR393866
127,0
F51TW9001AE4TW
BX251985(0/12)
LG5F
F51TW9001BR2I6
0,0
m994(4/18)
m19
CT574918(1/23)
CT580278(1/23)
1,3
AL749600(2/12)
BX249518(0/14)
BX252446(0/14)
CT578526
11,4
F51TW9001B7T52
BX251734(2/11)
16,3
BX252048(0/13)
m349(0/9)
estPtNCS_PtaAGP6
18,0
m658
BX680344
22,7
F51TW9001C8ABL(0/10)
F51TW9001CM780(0/11)
F51TW9001BV0Q9
40,2
BX680335(0/11)
F51TW9001BPA78(2/11)
F51TW9001CFMF0
46,2
FN256887
ssr_RPtEST11
49,4
BX249015
59,9
AL749752(4/11)
F51TW9001BYRJR(0/10)
FN256668
F51TW9001DBXSZ
62,3
BX250977(0/14)
BX252301(7/8)
BX254280
66,1
CT574697
BX680096(0/12)
75,1
CT579486(5/8)
m112(0/11)
ssr_NZPR823
78,4
estPtIFG_893(2/10)
CT576796
78,7
AL751280
CT577328
85,1
estPtMTU_PtCW1
85,9
F51TW9001AYUHT
89,9
BX250349(6/8)
BX251470(2/10)
CT583534
100,3
F51TW9001BQ0U8
BX249294(4/9)
BX677521(4/9)
CT578311(0/11)
102,8
estPtIFG_8779(0/5)
F51TW9001AFDBO(4/9)
F51TW9001BMC93(3/7)
F51TW9002G33Q5(4/9)
F51TW9002IGVQA(2/11)
F51TW9001A5SUZ
124,2
BX681875(0/14)
BX784243(6/9)
BX680419
130,1
F51TW9001B21AG(2/10)
m521
131,2
m520
F51TW9001AKCDD
131,3
F51TW9001BWV4H
BX681165(3/10)
AL749862
132,5
FN695223
133,8
BX251859
136,2
BX251985(0/11)
LG5_F2
FN256783
0,0
BX255673
0,8
F51TW9001BR2I6
BX253242
2,4
CT578845
3,2
m993
4,6
CT578526
14,3
BX251492
F51TW9001B7T52
17,5
m349
18,0
SNPnew59
24,7
m658
BX680344
25,4
F51TW9001C77R3
F51TW9001CM78O
25,6
BX248955
BX251734
27,3
BX253830
F51TW9001A4DYB
29,7
m1439
30,6
F51TW9001CBE6N
34,0
BX249114
36,4
CT576406
CT581734
37,1
BX249551
CT582973
37,8
F5X2MQL01EQT83
BX680977
54,9
AL749752
56,7
CT582219
m336
57,1
BX249015
58,5
m126
59,9
F51TW9001BYRJR
63,5
384_THE1
BX250977
FN256668
68,4
F51TW9001DBXSZ
BX254280
71,6
CT575145
BX254027
72,4
F51TW9001AE8M5
BX680096
79,6
CT579486
AL749624
80,8
F51TW9001BSSUH
F51TW9001E07A9
82,2
BX681656
83,8
BX251470
87,5
BX679256
92,6
F51TW9001AYUHT
F51TW9001BEO52
95,4
BX682051
98,3
CR393595
BX251120
99,9
BX677521
m1438
104,4
m23
107,8
BX249294
112,2
F51TW9002G33Q5
113,7
m307
123,3
m308
123,4
SNPnew45
CR393325
123,7
m184
124,5
AL749773
125,0
BX251430
127,4
CT577301
BX250757
128,2
F51TW9001BUY1L
F51TW9001AT3I3
129,8
F51TW9001A5SUZ
132,2
F51TW9001DS707
133,0
AL749723
133,9
F51TW9001BE00J(1/27)
SNPnew1
135,1
m964
137,2
m965
137,5
BX255657
F51TW9001ESH2T(1/26)
137,6
BX680240(1/26)
BX253126
F51TW9001B5SDZ
138,1
m521
140,9
m644
141,5
AL750490
141,8
m29
143,0
BX681165
148,2
F51TW9001B21AG
AL750931
149,0
BX665999
149,8
SNPnew95
151,3
BX678673
154,7
CR393866
155,3
F51TW9001AE4TW

## Slide 6
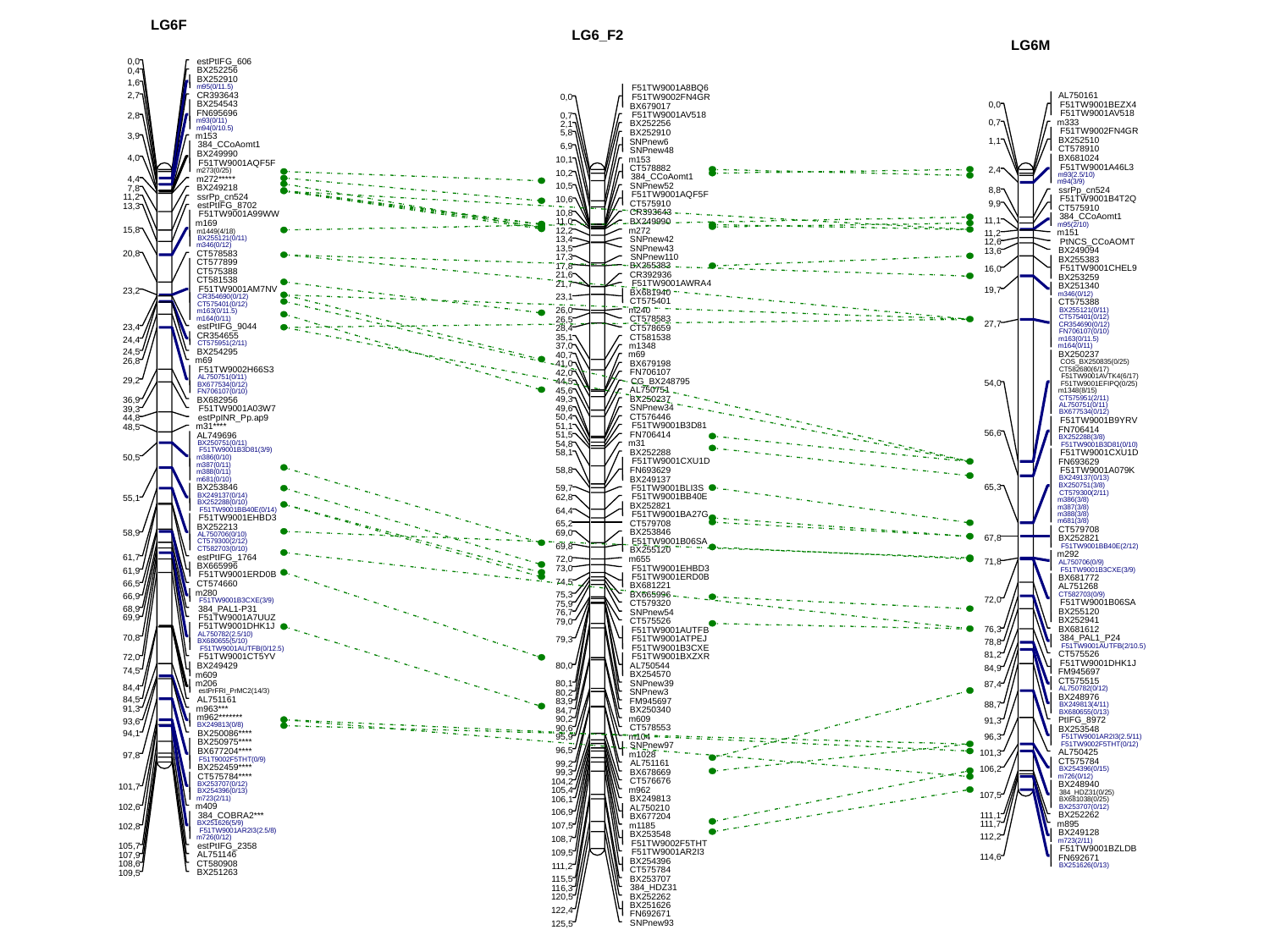

LG6F
estPtIFG_606
0,0
BX252256
0,4
BX252910
1,6
m95(0/11.5)
CR393643
2,7
BX254543
FN695696
2,8
m93(0/11)
m94(0/10.5)
m153
3,9
384_CCoAomt1
BX249990
4,0
F51TW9001AQF5F
m273(0/25)
m272*****
4,4
BX249218
7,8
ssrPp_cn524
11,2
estPtIFG_8702
13,3
F51TW9001A99WW
m169
15,8
m1449(4/18)
BX255121(0/11)
m346(0/12)
CT578583
20,8
CT577899
CT575388
CT581538
F51TW9001AM7NV
23,2
CR354690(0/12)
CT575401(0/12)
m163(0/11.5)
m164(0/11)
estPtIFG_9044
23,4
CR354655
24,4
CT575951(2/11)
BX254295
24,5
m69
26,8
F51TW9002H66S3
AL750751(0/11)
29,2
BX677534(0/12)
FN706107(0/10)
BX682956
36,9
F51TW9001A03W7
39,3
estPpINR_Pp.ap9
44,8
m31****
48,5
AL749696
BX250751(0/11)
F51TW9001B3D81(3/9)
50,5
m386(0/10)
m387(0/11)
m388(0/11)
m681(0/10)
BX253846
BX249137(0/14)
55,1
BX252288(0/10)
F51TW9001BB40E(0/14)
F51TW9001EHBD3
BX252213
58,9
AL750706(0/10)
CT579300(2/12)
CT582703(0/10)
estPtIFG_1764
61,7
BX665996
61,9
F51TW9001ERD0B
CT574660
66,5
m280
66,9
F51TW9001B3CXE(3/9)
384_PAL1-P31
68,9
F51TW9001A7UUZ
69,9
F51TW9001DHK1J
AL750782(2.5/10)
70,8
BX680655(5/10)
F51TW9001AUTFB(0/12.5)
F51TW9001CT5YV
72,0
BX249429
74,5
m609
m206
84,4
estPrFRI_PrMC2(14/3)
AL751161
84,5
m963***
91,3
m962*******
93,6
BX249813(0/8)
BX250086****
94,1
BX250975****
BX677204****
97,8
F51T9002F5THT(0/9)
BX252459****
CT575784****
BX253707(0/12)
101,7
BX254396(0/13)
m723(2/11)
m409
102,6
384_COBRA2***
BX251626(5/9)
102,8
F51TW9001AR2I3(2.5/8)
m726(0/12)
estPtIFG_2358
105,7
AL751146
107,9
CT580908
108,6
BX251263
109,5
LG6_F2
F51TW9001A8BQ6
F51TW9002FN4GR
0,0
BX679017
F51TW9001AV518
0,7
BX252256
2,1
BX252910
5,8
SNPnew6
6,9
SNPnew48
m153
10,1
CT578882
10,2
384_CCoAomt1
SNPnew52
10,5
F51TW9001AQF5F
10,6
CT575910
CR393643
10,8
BX249990
11,0
m272
12,2
SNPnew42
13,4
SNPnew43
13,5
SNPnew110
17,3
BX255383
17,8
CR392936
21,6
F51TW9001AWRA4
21,7
BX681940
23,1
CT575401
m240
26,0
CT578583
26,5
CT578659
28,4
CT581538
35,1
m1348
37,0
m69
40,7
BX679198
41,0
FN706107
42,0
CG_BX248795
44,5
AL750751
45,6
BX250237
49,3
SNPnew34
49,6
CT576446
50,4
F51TW9001B3D81
51,1
FN706414
51,5
m31
54,8
BX252288
58,1
F51TW9001CXU1D
FN693629
58,8
BX249137
F51TW9001BLI3S
59,7
F51TW9001BB40E
62,8
BX252821
64,4
F51TW9001BA27G
CT579708
65,2
BX253846
69,0
F51TW9001B06SA
69,8
BX255120
m655
72,0
F51TW9001EHBD3
73,0
F51TW9001ERD0B
74,5
BX681221
BX665996
75,3
CT579320
75,9
SNPnew54
76,7
CT575526
79,0
F51TW9001AUTFB
F51TW9001ATPEJ
79,3
F51TW9001B3CXE
F51TW9001BXZXR
AL750544
80,0
BX254570
SNPnew39
80,1
SNPnew3
80,2
FM945697
83,9
BX250340
84,7
m609
90,2
CT578553
90,6
m104
95,9
SNPnew97
96,5
m1028
AL751161
99,2
BX678669
99,3
CT576676
104,2
m962
105,4
BX249813
106,1
AL750210
106,9
BX677204
m1185
107,5
BX253548
108,7
F51TW9002F5THT
F51TW9001AR2I3
109,5
BX254396
111,2
CT575784
BX253707
115,5
384_HDZ31
116,3
BX252262
120,5
BX251626
122,4
FN692671
SNPnew93
125,5
LG6M
AL750161
F51TW9001BEZX4
0,0
F51TW9001AV518
m333
0,7
F51TW9002FN4GR
BX252510
1,1
CT578910
BX681024
F51TW9001A46L3
2,4
m93(2.5/10)
m94(3/9)
ssrPp_cn524
8,8
F51TW9001B4T2Q
9,9
CT575910
384_CCoAomt1
11,1
m95(2/10)
m151
11,2
PtNCS_CCoAOMT
12,6
BX249094
13,6
BX255383
F51TW9001CHEL9
16,0
BX253259
BX251340
19,7
m346(0/12)
CT575388
BX255121(0/11)
CT575401(0/12)
27,7
CR354690(0/12)
FN706107(0/10)
m163(0/11.5)
m164(0/11)
BX250237
COS_BX250835(0/25)
CT582680(6/17)
F51TW9001AVTK4(6/17)
54,0
F51TW9001EFIPQ(0/25)
m1348(8/15)
CT575951(2/11)
AL750751(0/11)
BX677534(0/12)
F51TW9001B9YRV
FN706414
56,6
BX252288(3/8)
F51TW9001B3D81(0/10)
F51TW9001CXU1D
FN693629
F51TW9001A079K
BX249137(0/13)
BX250751(3/8)
65,3
CT579300(2/11)
m386(3/8)
m387(3/8)
m388(3/8)
m681(3/8)
CT579708
67,8
BX252821
F51TW9001BB40E(2/12)
m292
71,8
AL750706(0/9)
F51TW9001B3CXE(3/9)
BX681772
AL751268
CT582703(0/9)
72,0
F51TW9001B06SA
BX255120
BX252941
BX681612
76,3
384_PAL1_P24
78,8
F51TW9001AUTFB(2/10.5)
CT575526
81,2
F51TW9001DHK1J
84,9
FM945697
CT575515
87,4
AL750782(0/12)
BX248976
88,7
BX249813(4/11)
BX680655(0/13)
PtIFG_8972
91,3
BX253548
96,3
F51TW9001AR2I3(2.5/11)
F51TW9002F5THT(0/12)
AL750425
101,3
CT575784
106,2
BX254396(0/15)
m726(0/12)
BX248940
384_HDZ31(0/25)
107,5
BX681038(0/25)
BX253707(0/12)
BX252262
111,1
m895
111,7
BX249128
112,2
m723(2/11)
F51TW9001BZLDB
114,6
FN692671
BX251626(0/13)

## Slide 7
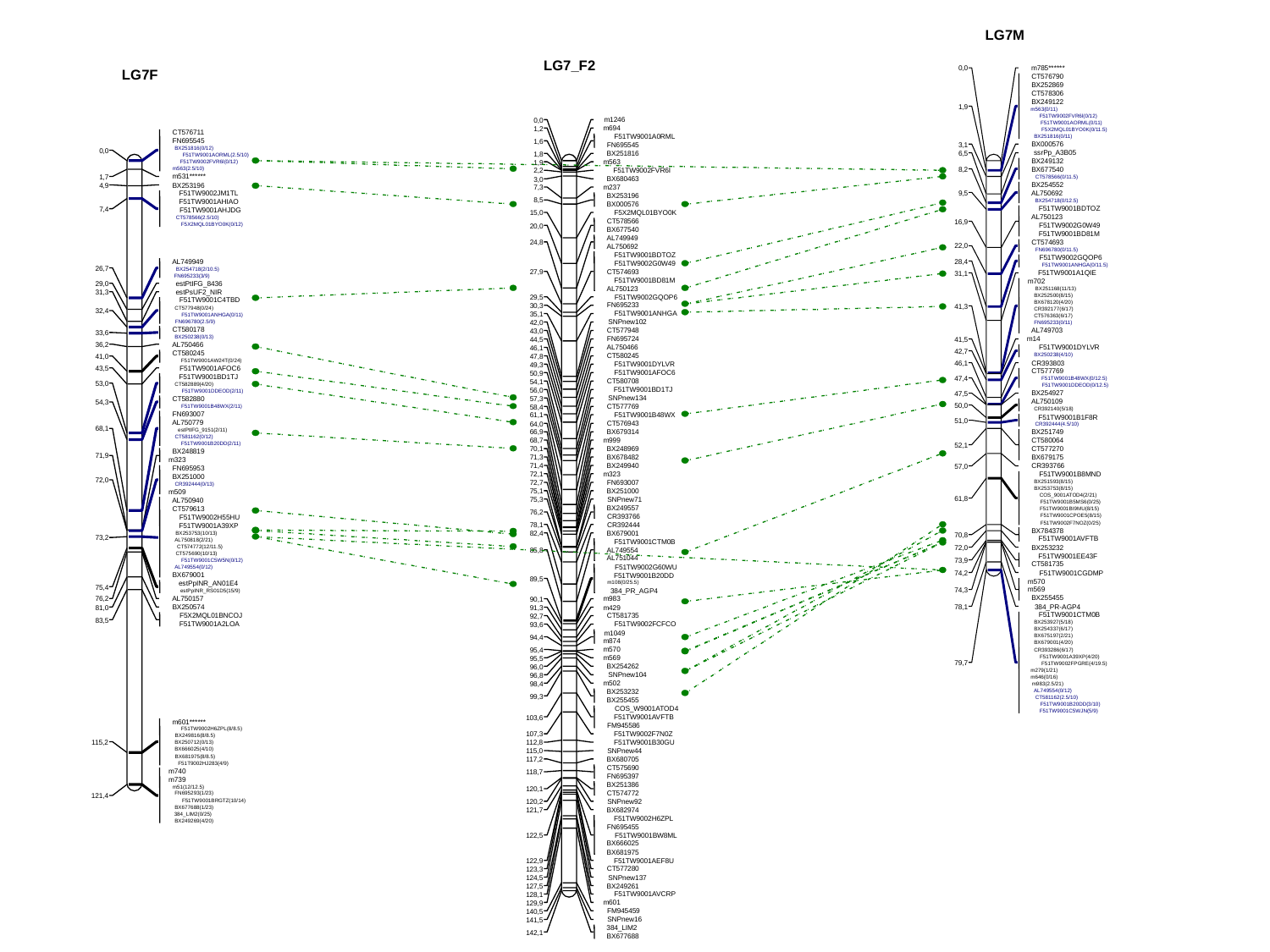

LG7M
m785******
0,0
CT576790
BX252869
CT578306
BX249122
1,9
m563(0/11)
F51TW9002FVR6l(0/12)
F51TW9001AORML(0/11)
F5X2MQL01BYO0K(0/11.5)
BX251816(0/11)
BX000576
3,1
ssrPp_A3B05
6,5
BX249132
8,2
BX677540
CT578566(0/11.5)
BX254552
9,5
AL750692
BX254718(0/12.5)
F51TW9001BDTOZ
AL750123
16,9
F51TW9002G0W49
F51TW9001BD81M
CT574693
22,0
FN696780(0/11.5)
F51TW9002GQOP6
28,4
F51TW9001ANHGA(0/11.5)
F51TW9001A1QIE
31,1
m702
BX251168(11/13)
BX252500(8/15)
BX678120(4/20)
41,3
CR392177(6/17)
CT576363(6/17)
FN695233(0/11)
AL749703
m14
41,5
F51TW9001DYLVR
42,7
BX250238(4/10)
CR393803
46,1
CT577769
47,4
F51TW9001B48WX(0/12.5)
F51TW9001DDEOD(0/12.5)
BX254927
47,5
AL750109
50,0
CR392140(5/18)
F51TW9001B1F8R
51,0
CR392444(4.5/10)
BX251749
CT580064
52,1
CT577270
BX679175
CR393766
57,0
F51TW9001B8MND
BX251593(8/15)
BX253753(8/15)
COS_9001ATOD4(2/21)
61,8
F51TW9001B5MS6(0/25)
F51TW9001BI9MU(8/15)
F51TW9001CPOE5(8/15)
F51TW9002F7NOZ(0/25)
BX784378
70,8
F51TW9001AVFTB
BX253232
72,0
F51TW9001EE43F
73,9
CT581735
F51TW9001CGDMP
74,2
m570
m569
74,3
BX255455
384_PR-AGP4
78,1
F51TW9001CTM0B
BX253927(5/18)
BX254337(6/17)
BX675197(2/21)
BX679001(4/20)
CR393286(6/17)
F51TW9001A39XP(4/20)
79,7
F51TW9002FPGRE(4/19.5)
m279(1/21)
m646(0/16)
m983(2.5/21)
AL749554(0/12)
CT581162(2.5/10)
F51TW9001B20DD(3/10)
F51TW9001C5WJN(5/9)
LG7_F2
m1246
0,0
m694
1,2
F51TW9001A0RML
1,6
FN695545
BX251816
1,8
m563
1,9
F51TW9002FVR6I
2,2
BX680463
3,0
m237
7,3
BX253196
8,5
BX000576
F5X2MQL01BYO0K
15,0
CT578566
20,0
BX677540
AL749949
24,8
AL750692
F51TW9001BDTOZ
F51TW9002G0W49
CT574693
27,9
F51TW9001BD81M
AL750123
F51TW9002GQOP6
29,5
FN695233
30,3
F51TW9001ANHGA
35,1
SNPnew102
42,0
CT577948
43,0
FN695724
44,5
AL750466
46,1
CT580245
47,8
F51TW9001DYLVR
49,3
F51TW9001AFOC6
50,9
CT580708
54,1
F51TW9001BD1TJ
56,0
SNPnew134
57,3
CT577769
58,4
F51TW9001B48WX
61,1
CT576943
64,0
BX679314
66,9
m999
68,7
BX248969
70,1
BX678482
71,3
BX249940
71,4
m323
72,1
FN693007
72,7
BX251000
75,1
SNPnew71
75,3
BX249557
76,2
CR393766
CR392444
78,1
BX679001
82,4
F51TW9001CTM0B
AL749554
85,8
AL751044
F51TW9002G60WU
F51TW9001B20DD
89,5
m108(0/25.5)
384_PR_AGP4
m983
90,1
m429
91,3
CT581735
92,7
F51TW9002FCFCO
93,6
m1049
94,4
m874
m570
95,4
m569
95,5
BX254262
96,0
SNPnew104
96,8
m502
98,4
BX253232
99,3
BX255455
COS_W9001ATOD4
F51TW9001AVFTB
103,6
FM945586
F51TW9002F7N0Z
107,3
F51TW9001B30GU
112,8
SNPnew44
115,0
BX680705
117,2
CT575690
118,7
FN695397
BX251386
120,1
CT574772
SNPnew92
120,2
BX682974
121,7
F51TW9002H6ZPL
FN695455
F51TW9001BW8ML
122,5
BX666025
BX681975
F51TW9001AEF8U
122,9
CT577280
123,3
SNPnew137
124,5
BX249261
127,5
F51TW9001AVCRP
128,1
m601
129,9
FM945459
140,5
SNPnew16
141,5
384_LIM2
142,1
BX677688
LG7F
CT576711
FN695545
BX251816(0/12)
0,0
F51TW9001AORML(2.5/10)
F51TW9002FVR6l(0/12)
m563(2.5/10)
m531******
1,7
BX253196
4,9
F51TW9002JM1TL
F51TW9001AHIAO
7,4
F51TW9001AHJDG
CT578566(2.5/10)
F5X2MQL01BYO0K(0/12)
AL749949
26,7
BX254718(2/10.5)
FN695233(3/9)
estPtIFG_8436
29,0
estPsUF2_NIR
31,3
F51TW9001C4TBD
CT577948(0/24)
32,4
F51TW9001ANHGA(0/11)
FN696780(2.5/9)
CT580178
33,6
BX250238(0/13)
AL750466
36,2
CT580245
41,0
F51TW9001AW24T(0/24)
F51TW9001AFOC6
43,5
F51TW9001BD1TJ
53,0
CT582889(4/20)
F51TW9001DDEOD(2/11)
CT582880
54,3
F51TW9001B48WX(2/11)
FN693007
AL750779
68,1
estPtIFG_9151(2/11)
CT581162(0/12)
F51TW9001B20DD(2/11)
BX248819
71,9
m323
FN695953
BX251000
72,0
CR392444(0/13)
m509
AL750940
CT579613
F51TW9002H55HU
F51TW9001A39XP
BX253753(10/13)
73,2
AL750818(2/21)
CT574772(12/11.5)
CT575690(10/13)
F51TW9001C5W5N(0/12)
AL749554(0/12)
BX679001
estPpINR_AN01E4
75,4
estPpINR_RS01D5(15/9)
AL750157
76,2
BX250574
81,0
F5X2MQL01BNCOJ
83,5
F51TW9001A2LOA
m601******
F51TW9002H6ZPL(8/8.5)
BX249816(8/8.5)
115,2
BX250712(0/13)
BX666025(4/10)
BX681975(8/8.5)
F51T9002HJ283(4/9)
m740
m739
m51(12/12.5)
FN695293(1/23)
121,4
F51TW9001BRGTZ(10/14)
BX677688(1/23)
384_LIM2(0/25)
BX249269(4/20)

## Slide 8
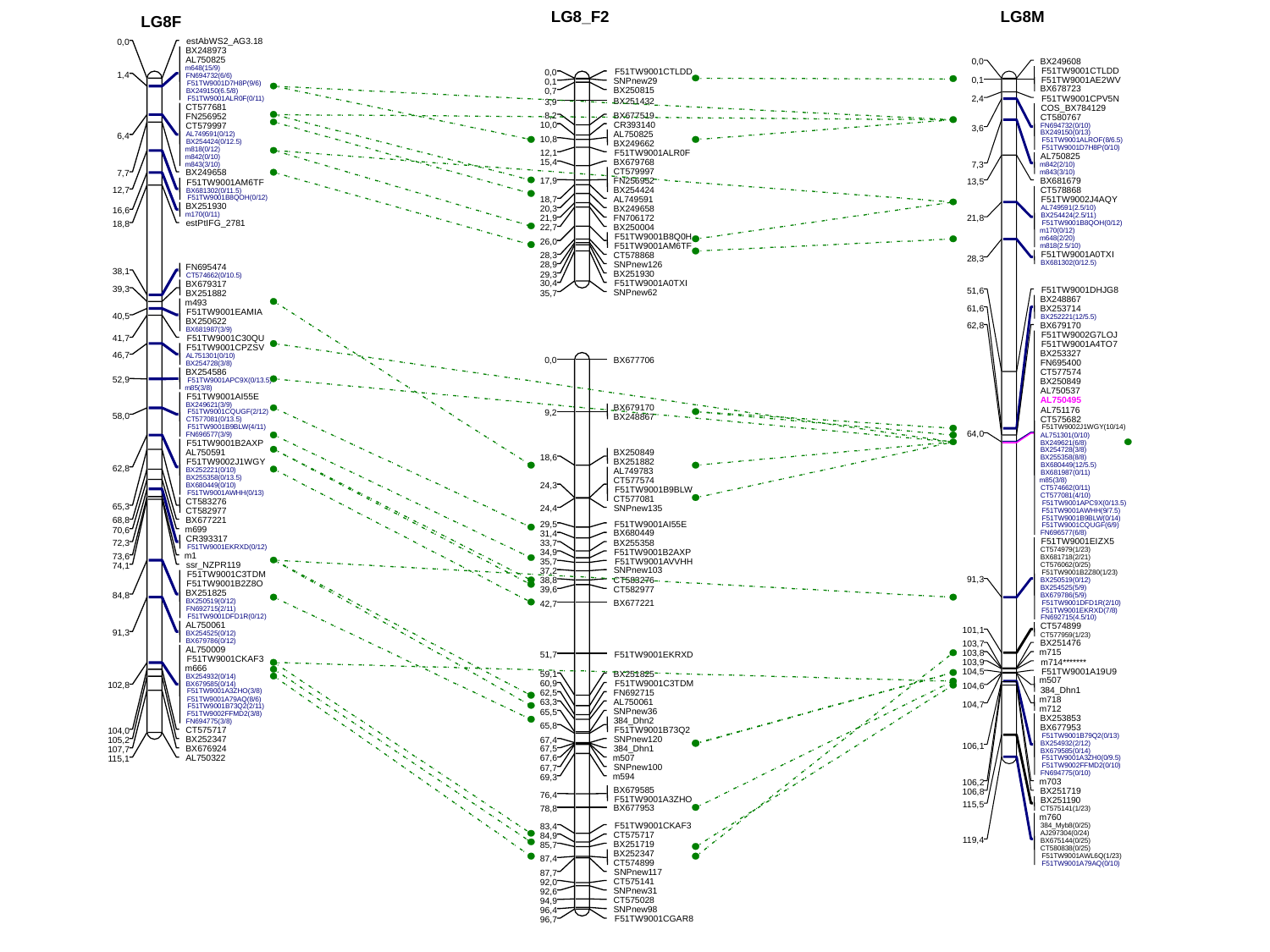

LG8_F2
F51TW9001CTLDD
0,0
SNPnew29
0,1
BX250815
0,7
BX251432
3,9
BX677519
8,2
CR393140
10,0
AL750825
10,8
BX249662
F51TW9001ALR0F
12,1
BX679768
15,4
CT579997
FN256952
17,9
BX254424
AL749591
18,7
BX249658
20,3
FN706172
21,9
BX250004
22,7
F51TW9001B8Q0H
26,0
F51TW9001AM6TF
CT578868
28,3
SNPnew126
28,9
BX251930
29,3
F51TW9001A0TXI
30,4
SNPnew62
35,7
BX677706
0,0
BX679170
9,2
BX248867
BX250849
18,6
BX251882
AL749783
CT577574
24,3
F51TW9001B9BLW
CT577081
SNPnew135
24,4
F51TW9001AI55E
29,5
BX680449
31,4
BX255358
33,7
F51TW9001B2AXP
34,9
F51TW9001AVVHH
35,7
SNPnew103
37,2
CT583276
38,8
CT582977
39,6
BX677221
42,7
F51TW9001EKRXD
51,7
BX251825
59,1
F51TW9001C3TDM
60,9
FN692715
62,5
AL750061
63,3
SNPnew36
65,5
384_Dhn2
65,8
F51TW9001B73Q2
SNPnew120
67,4
384_Dhn1
67,5
m507
67,6
SNPnew100
67,7
m594
69,3
BX679585
76,4
F51TW9001A3ZHO
BX677953
78,8
F51TW9001CKAF3
83,4
CT575717
84,9
BX251719
85,7
BX252347
87,4
CT574899
SNPnew117
87,7
CT575141
92,0
SNPnew31
92,6
CT575028
94,9
SNPnew98
96,4
F51TW9001CGAR8
96,7
LG8M
BX249608
0,0
F51TW9001CTLDD
F51TW9001AE2WV
0,1
BX678723
F51TW9001CPV5N
2,4
COS_BX784129
CT580767
FN694732(0/10)
3,6
BX249150(0/13)
F51TW9001ALROF(8/6.5)
F51TW9001D7H8P(0/10)
AL750825
7,3
m842(2/10)
m843(3/10)
BX681679
13,5
CT578868
F51TW9002J4AQY
AL749591(2.5/10)
BX254424(2.5/11)
21,8
F51TW9001B8QOH(0/12)
m170(0/12)
m648(2/20)
m818(2.5/10)
F51TW9001A0TXI
28,3
BX681302(0/12.5)
F51TW9001DHJG8
51,6
BX248867
61,6
BX253714
BX252221(12/5.5)
BX679170
62,8
F51TW9002G7LOJ
F51TW9001A4TO7
BX253327
FN695400
CT577574
BX250849
AL750537
AL750495
AL751176
CT575682
F51TW9002J1WGY(10/14)
64,0
AL751301(0/10)
BX249621(6/8)
BX254728(3/8)
BX255358(8/8)
BX680449(12/5.5)
BX681987(0/11)
m85(3/8)
CT574662(0/11)
CT577081(4/10)
F51TW9001APC9X(0/13.5)
F51TW9001AWHH(9/7.5)
F51TW9001B9BLW(0/14)
F51TW9001CQUGF(6/9)
FN696577(6/8)
F51TW9001EIZX5
CT574979(1/23)
BX681718(2/21)
CT576062(0/25)
F51TW9001B2Z80(1/23)
91,3
BX250519(0/12)
BX254525(5/9)
BX679786(5/9)
F51TW9001DFD1R(2/10)
F51TW9001EKRXD(7/8)
FN692715(4.5/10)
CT574899
101,1
CT577959(1/23)
BX251476
103,7
m715
103,8
m714*******
103,9
F51TW9001A19U9
104,5
m507
104,6
384_Dhn1
m718
104,7
m712
BX253853
BX677953
F51TW9001B79Q2(0/13)
BX254932(2/12)
106,1
BX679585(0/14)
F51TW9001A3ZH0(0/9.5)
F51TW9002FFMD2(0/10)
FN694775(0/10)
m703
106,2
BX251719
106,8
BX251190
115,5
CT575141(1/23)
m760
384_Myb8(0/25)
AJ297304(0/24)
119,4
BX675144(0/25)
CT580838(0/25)
F51TW9001AWL6Q(1/23)
F51TW9001A79AQ(0/10)
LG8F
estAbWS2_AG3.18
0,0
BX248973
AL750825
m648(15/9)
1,4
FN694732(6/6)
F51TW9001D7H8P(9/6)
BX249150(6.5/8)
F51TW9001ALR0F(0/11)
CT577681
FN256952
CT579997
AL749591(0/12)
6,4
BX254424(0/12.5)
m818(0/12)
m842(0/10)
m843(3/10)
BX249658
7,7
F51TW9001AM6TF
12,7
BX681302(0/11.5)
F51TW9001B8QOH(0/12)
BX251930
16,6
m170(0/11)
estPtIFG_2781
18,8
FN695474
38,1
CT574662(0/10.5)
BX679317
39,3
BX251882
m493
F51TW9001EAMIA
40,5
BX250622
BX681987(3/9)
F51TW9001C30QU
41,7
F51TW9001CPZSV
46,7
AL751301(0/10)
BX254728(3/8)
BX254586
52,9
F51TW9001APC9X(0/13.5)
m85(3/8)
F51TW9001AI55E
BX249621(3/9)
F51TW9001CQUGF(2/12)
58,0
CT577081(0/13.5)
F51TW9001B9BLW(4/11)
FN696577(3/9)
F51TW9001B2AXP
AL750591
F51TW9002J1WGY
62,8
BX252221(0/10)
BX255358(0/13.5)
BX680449(0/10)
F51TW9001AWHH(0/13)
CT583276
65,3
CT582977
BX677221
68,8
m699
70,6
CR393317
72,3
F51TW9001EKRXD(0/12)
m1
73,6
ssr_NZPR119
74,1
F51TW9001C3TDM
F51TW9001B2Z8O
BX251825
84,8
BX250519(0/12)
FN692715(2/11)
F51TW9001DFD1R(0/12)
AL750061
91,3
BX254525(0/12)
BX679786(0/12)
AL750009
F51TW9001CKAF3
m666
BX254932(0/14)
BX679585(0/14)
102,8
F51TW9001A3ZHO(3/8)
F51TW9001A79AQ(8/6)
F51TW9001B73Q2(2/11)
F51TW9002FFMD2(3/8)
FN694775(3/8)
CT575717
104,0
BX252347
105,2
BX676924
107,7
AL750322
115,1

## Slide 9
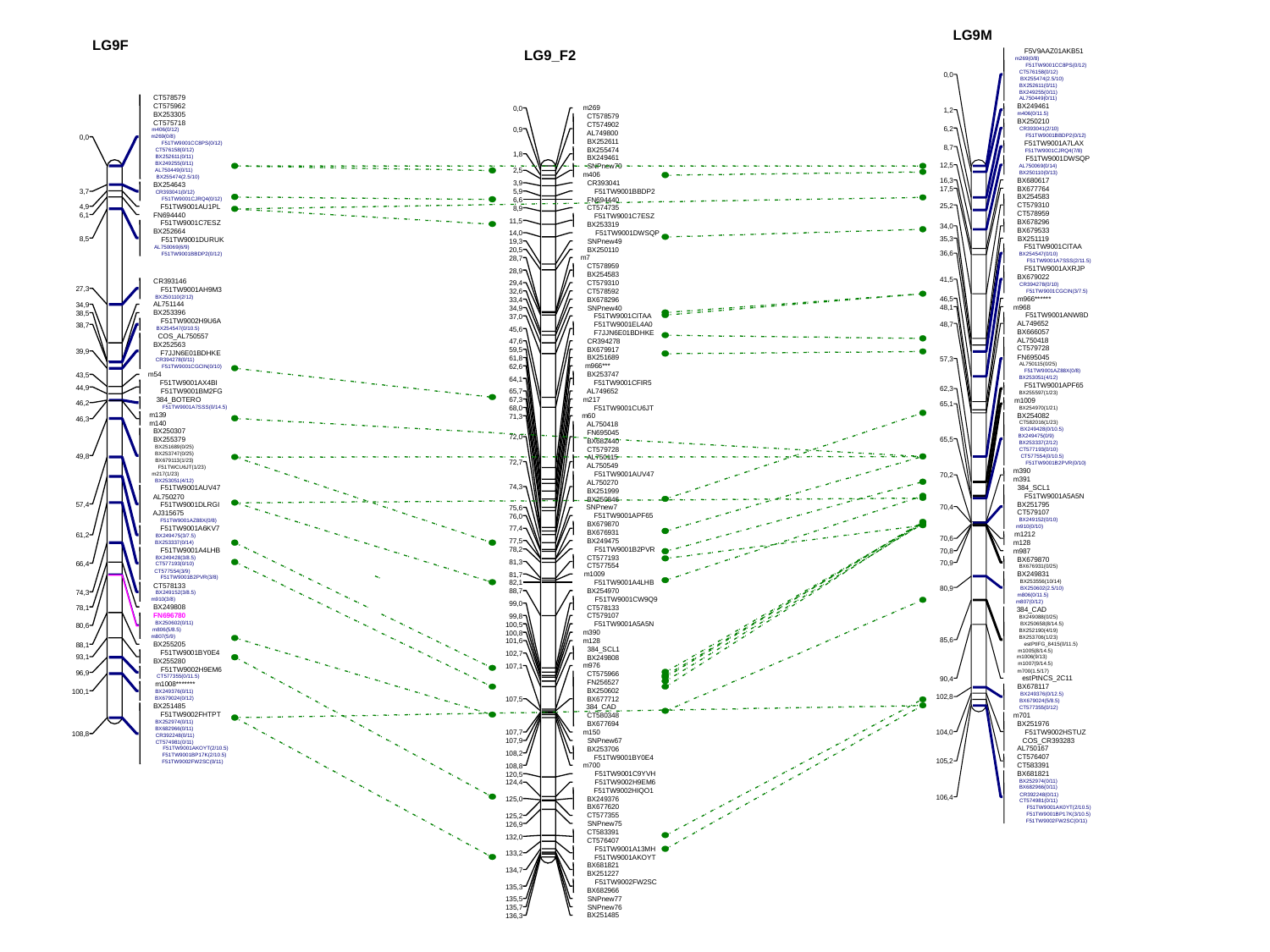

LG9M
F5V9AAZ01AKB51
m269(0/8)
F51TW9001CC8PS(0/12)
CT576158(0/12)
0,0
BX255474(2.5/10)
BX252611(0/11)
BX249255(0/11)
AL750449(0/11)
BX249461
1,2
m406(0/11.5)
BX250210
6,2
CR393041(2/10)
F51TW9001BBDP2(0/12)
F51TW9001A7LAX
8,7
F51TW9001CJRQ4(7/8)
F51TW9001DWSQP
12,5
AL750069(0/14)
BX250110(0/13)
BX680617
16,3
BX677764
17,5
BX254583
CT579310
25,2
CT578959
BX678296
34,0
BX679533
BX251119
35,3
F51TW9001CITAA
36,6
BX254547(0/10)
F51TW9001A7SSS(2/11.5)
F51TW9001AXRJP
BX679022
41,5
CR394278(0/10)
F51TW9001CGCIN(3/7.5)
m966******
46,5
m968
48,1
F51TW9001ANW8D
AL749652
48,7
BX666057
AL750418
CT579728
FN695045
57,3
AL750115(0/25)
F51TW9001AZ88X(0/8)
BX253051(4/12)
F51TW9001APF65
62,3
BX255597(1/23)
m1009
65,1
BX254970(1/21)
BX254082
CT582016(1/23)
BX249428(0/10.5)
BX249475(0/9)
65,5
BX253337(2/12)
CT577193(0/10)
CT577554(0/10.5)
F51TW9001B2PVR(0/10)
m390
70,2
m391
384_SCL1
F51TW9001A5A5N
BX251795
70,4
CT579107
BX249152(0/10)
m910(0/10)
m1212
70,6
m128
m987
70,8
BX679870
70,9
BX676931(0/25)
BX249831
BX253556(10/14)
80,9
BX250602(2.5/10)
m806(0/11.5)
m807(0/12)
384_CAD
BX249088(0/25)
BX250658(8/14.5)
BX252190(4/19)
BX253706(1/23)
85,6
estPtIFG_8415(0/11.5)
m1005(8/14.5)
m1006(9/13)
m1007(9/14.5)
m700(1.5/17)
estPtNCS_2C11
90,4
BX678117
BX249376(0/12.5)
102,8
BX679024(5/8.5)
CT577355(0/12)
m701
BX251976
F51TW9002HSTUZ
COS_CR393283
AL750167
104,0
CT576407
105,2
CT583391
BX681821
BX252974(0/11)
BX682966(0/11)
CR392248(0/11)
106,4
CT574981(0/11)
F51TW9001AK0YT(2/10.5)
F51TW9001BP17K(3/10.5)
F51TW9002FW2SC(0/11)
LG9F
CT578579
CT575962
BX253305
CT575718
m406(0/12)
0,0
m269(0/8)
F51TW9001CC8PS(0/12)
CT576158(0/12)
BX252611(0/11)
BX249255(0/11)
AL750449(0/11)
BX255474(2.5/10)
BX254643
3,7
CR393041(0/12)
F51TW9001CJRQ4(0/12)
F51TW9001AU1PL
4,9
FN694440
6,1
F51TW9001C7ESZ
BX252664
8,5
F51TW9001DURUK
AL750069(6/9)
F51TW9001BBDP2(0/12)
CR393146
27,3
F51TW9001AH9M3
BX250110(2/12)
AL751144
34,9
BX253396
38,5
F51TW9002H9U6A
38,7
BX254547(0/10.5)
COS_AL750557
BX252563
39,9
F7JJN6E01BDHKE
CR394278(0/11)
F51TW9001CGCIN(0/10)
m54
43,5
F51TW9001AX4BI
44,9
F51TW9001BM2FG
384_BOTERO
46,2
F51TW9001A7SSS(0/14.5)
m139
46,3
m140
BX250307
BX255379
BX251689(0/25)
BX253747(0/25)
49,8
BX679113(1/23)
F51TWCU6JT(1/23)
m217(1/23)
BX253051(4/12)
F51TW9001AUV47
AL750270
57,4
F51TW9001DLRGI
AJ315675
F51TW9001AZ88X(0/8)
F51TW9001A6KV7
61,2
BX249475(3/7.5)
BX253337(0/14)
F51TW9001A4LHB
BX249428(3/8.5)
66,4
CT577193(0/10)
CT577554(3/9)
F51TW9001B2PVR(3/8)
CT578133
74,3
BX249152(3/8.5)
m910(3/8)
BX249808
78,1
FN696780
BX250602(0/11)
80,6
m806(5/8.5)
m807(5/9)
BX255205
88,1
F51TW9001BY0E4
93,1
BX255280
F51TW9002H9EM6
96,9
CT577355(0/11.5)
m1008*******
100,1
BX249376(0/11)
BX679024(0/12)
BX251485
F51TW9002FHTPT
BX252974(0/11)
BX682966(0/11)
108,8
CR392248(0/11)
CT574981(0/11)
F51TW9001AKOYT(2/10.5)
F51TW9001BP17K(2/10.5)
F51TW9002FW2SC(0/11)
LG9_F2
m269
0,0
CT578579
CT574902
0,9
AL749800
BX252611
BX255474
1,8
BX249461
SNPnew70
2,5
m406
CR393041
3,9
F51TW9001BBDP2
5,9
FN694440
6,6
CT574735
8,9
F51TW9001C7ESZ
11,5
BX253319
F51TW9001DWSQP
14,0
SNPnew49
19,3
BX250110
20,5
m7
28,7
CT578959
28,9
BX254583
CT579310
29,4
CT578592
32,6
BX678296
33,4
SNPnew40
34,9
F51TW9001CITAA
37,0
F51TW9001EL4A0
45,6
F7JJN6E01BDHKE
CR394278
47,6
BX679917
59,5
BX251689
61,8
m966***
62,6
BX253747
64,1
F51TW9001CFIR5
AL749652
65,7
m217
67,3
F51TW9001CU6JT
68,0
m60
71,3
AL750418
FN695045
72,0
BX682440
CT579728
AL750115
72,7
AL750549
F51TW9001AUV47
AL750270
74,3
BX251999
BX250846
SNPnew7
75,6
F51TW9001APF65
76,0
BX679870
77,4
BX676931
BX249475
77,5
F51TW9001B2PVR
78,2
CT577193
81,3
CT577554
m1009
81,7
F51TW9001A4LHB
82,1
BX254970
88,7
F51TW9001CW9Q9
99,0
CT578133
CT579107
99,8
F51TW9001A5A5N
100,5
m390
100,8
m128
101,6
384_SCL1
102,7
BX249808
m976
107,1
CT575966
FN256527
BX250602
BX677712
107,5
384_CAD
CT580348
BX677694
m150
107,7
SNPnew67
107,9
BX253706
108,2
F51TW9001BY0E4
m700
108,8
F51TW9001C9YVH
120,5
F51TW9002H9EM6
124,4
F51TW9002HIQO1
BX249376
125,0
BX677620
CT577355
125,2
SNPnew75
126,9
CT583391
132,0
CT576407
F51TW9001A13MH
133,2
F51TW9001AKOYT
BX681821
134,7
BX251227
F51TW9002FW2SC
135,3
BX682966
SNPnew77
135,5
SNPnew76
135,7
BX251485
136,3

## Slide 10
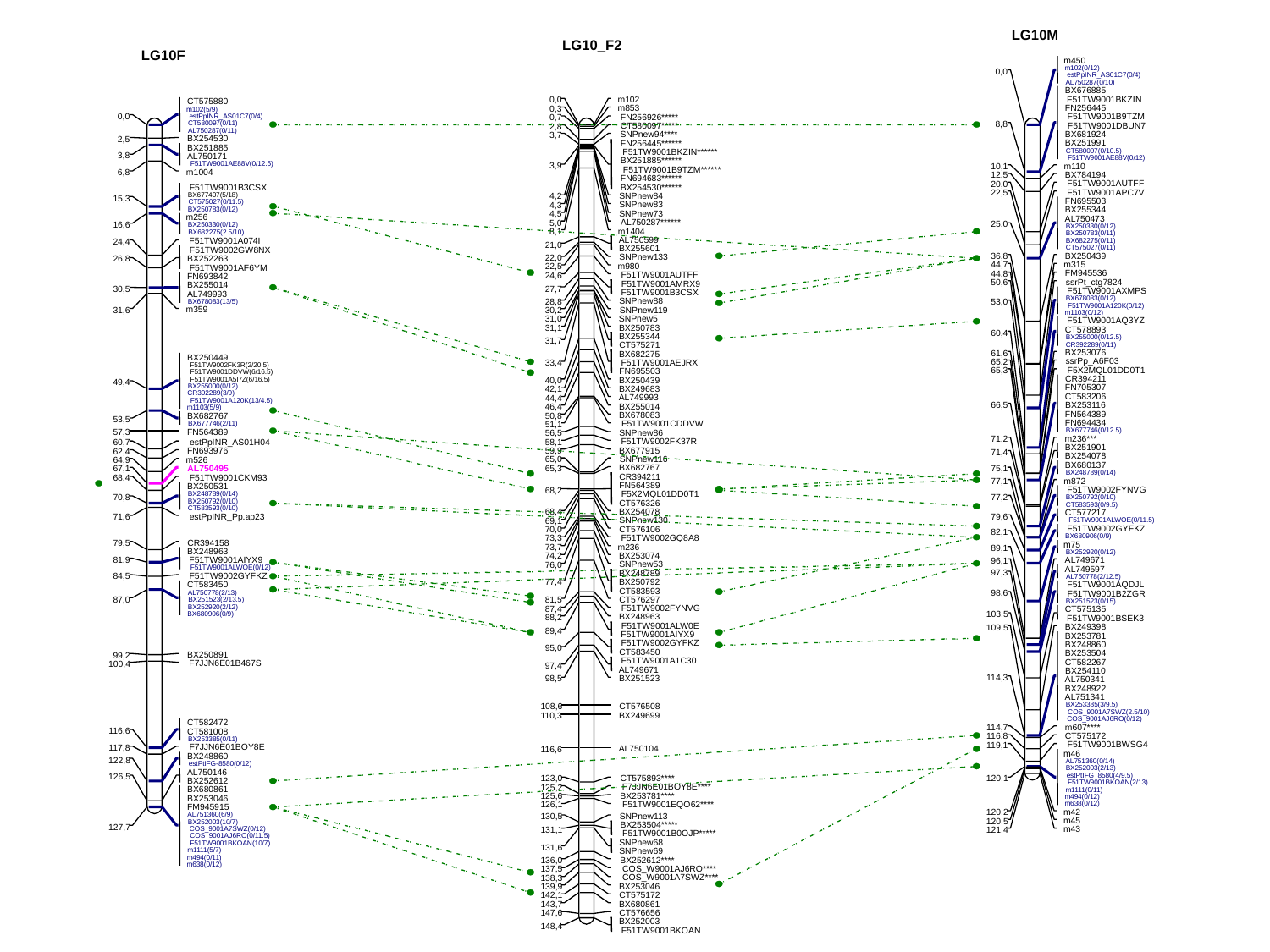

LG10M
m450
m102(0/12)
0,0
estPpINR_AS01C7(0/4)
AL750287(0/10)
BX676885
F51TW9001BKZIN
FN256445
F51TW9001B9TZM
8,8
F51TW9001DBUN7
BX681924
BX251991
CT580097(0/10.5)
F51TW9001AE88V(0/12)
m110
10,1
BX784194
12,5
F51TW9001AUTFF
20,0
F51TW9001APC7V
22,5
FN695503
BX255344
AL750473
25,0
BX250330(0/12)
BX250783(0/11)
BX682275(0/11)
CT575027(0/11)
BX250439
36,8
m315
44,7
FM945536
44,8
ssrPt_ctg7824
50,6
F51TW9001AXMPS
BX678083(0/12)
53,0
F51TW9001A120K(0/12)
m1103(0/12)
F51TW9001AQ3YZ
CT578893
60,4
BX255000(0/12.5)
CR392289(0/11)
BX253076
61,6
ssrPp_A6F03
65,2
F5X2MQL01DD0T1
65,3
CR394211
FN705307
CT583206
66,5
BX253116
FN564389
FN694434
BX677746(0/12.5)
m236***
71,2
BX251901
71,4
BX254078
BX680137
75,1
BX248789(0/14)
m872
77,1
F51TW9002FYNVG
77,2
BX250792(0/10)
CT583593(0/9.5)
CT577217
79,6
F51TW9001ALWOE(0/11.5)
F51TW9002GYFKZ
82,1
BX680906(0/9)
m75
89,1
BX252920(0/12)
AL749671
96,1
AL749597
97,3
AL750778(2/12.5)
F51TW9001AQDJL
98,6
F51TW9001B2ZGR
BX251523(0/15)
CT575135
103,5
F51TW9001BSEK3
BX249398
109,5
BX253781
BX248860
BX253504
CT582267
BX254110
114,3
AL750341
BX248922
AL751341
BX253385(3/9.5)
COS_9001A7SWZ(2.5/10)
COS_9001AJ6RO(0/12)
m607****
114,7
CT575172
116,8
F51TW9001BWSG4
119,1
m46
AL751360(0/14)
BX252003(2/13)
estPtIFG_8580(4/9.5)
120,1
F51TW9001BKOAN(2/13)
m1111(0/11)
m494(0/12)
m638(0/12)
m42
120,2
m45
120,5
m43
121,4
LG10_F2
m102
0,0
m853
0,3
FN256926*****
0,7
CT580097*****
2,8
SNPnew94****
3,7
FN256445******
F51TW9001BKZIN******
BX251885******
3,9
F51TW9001B9TZM******
FN694683******
BX254530******
SNPnew84
4,2
SNPnew83
4,3
SNPnew73
4,5
AL750287******
5,0
m1404
8,1
AL750599
21,0
BX255601
SNPnew133
22,0
m980
22,5
F51TW9001AUTFF
24,6
F51TW9001AMRX9
27,7
F51TW9001B3CSX
SNPnew88
28,8
SNPnew119
30,2
SNPnew5
31,0
BX250783
31,1
BX255344
31,7
CT575271
BX682275
F51TW9001AEJRX
33,4
FN695503
BX250439
40,0
BX249683
42,1
AL749993
44,4
BX255014
46,4
BX678083
50,8
F51TW9001CDDVW
51,1
SNPnew86
56,5
F51TW9002FK37R
58,1
BX677915
59,9
SNPnew116
65,0
BX682767
65,3
CR394211
FN564389
68,2
F5X2MQL01DD0T1
CT576326
BX254078
68,4
SNPnew130
69,1
CT576106
70,0
F51TW9002GQ8A8
73,3
m236
73,7
BX253074
74,2
SNPnew53
76,0
BX248789
BX250792
77,4
CT583593
CT576297
81,5
F51TW9002FYNVG
87,4
BX248963
88,2
F51TW9001ALW0E
89,4
F51TW9001AIYX9
F51TW9002GYFKZ
95,0
CT583450
F51TW9001A1C30
97,4
AL749671
BX251523
98,5
CT576508
108,6
BX249699
110,3
AL750104
116,6
CT575893****
123,0
F7JJN6E01BOY8E****
125,2
BX253781****
125,6
F51TW9001EQO62****
126,1
SNPnew113
130,5
BX253504*****
131,1
F51TW9001B0OJP*****
SNPnew68
131,6
SNPnew69
BX252612****
136,0
COS_W9001AJ6RO****
137,5
COS_W9001A7SWZ****
138,3
BX253046
139,9
CT575172
142,1
BX680861
143,7
CT576656
147,6
BX252003
148,4
F51TW9001BKOAN
LG10F
CT575880
m102(5/9)
0,0
estPpINR_AS01C7(0/4)
CT580097(0/11)
AL750287(0/11)
BX254530
2,5
BX251885
3,8
AL750171
F51TW9001AE88V(0/12.5)
m1004
6,8
F51TW9001B3CSX
BX677407(5/18)
15,3
CT575027(0/11.5)
BX250783(0/12)
m256
16,6
BX250330(0/12)
BX682275(2.5/10)
F51TW9001A074I
24,4
F51TW9002GW8NX
BX252263
26,8
F51TW9001AF6YM
FN693842
BX255014
30,5
AL749993
BX678083(13/5)
m359
31,6
BX250449
F51TW9002FK3R(2/20.5)
F51TW9001DDVW(6/16.5)
F51TW9001A5I7Z(6/16.5)
49,4
BX255000(0/12)
CR392289(3/9)
F51TW9001A120K(13/4.5)
m1103(5/9)
BX682767
53,5
BX677746(2/11)
FN564389
57,3
estPpINR_AS01H04
60,7
FN693976
62,4
m526
64,9
AL750495
67,1
F51TW9001CKM93
68,4
BX250531
BX248789(0/14)
70,8
BX250792(0/10)
CT583593(0/10)
estPpINR_Pp.ap23
71,6
CR394158
79,5
BX248963
81,9
F51TW9001AIYX9
F51TW9001ALWOE(0/12)
F51TW9002GYFKZ
84,5
CT583450
AL750778(2/13)
87,0
BX251523(2/13.5)
BX252920(2/12)
BX680906(0/9)
BX250891
99,2
F7JJN6E01B467S
100,4
CT582472
116,6
CT581008
BX253385(0/11)
F7JJN6E01BOY8E
117,8
BX248860
122,8
estPtIFG-8580(0/12)
AL750146
126,5
BX252612
BX680861
BX253046
FM945915
AL751360(6/9)
BX252003(10/7)
127,7
COS_9001A7SWZ(0/12)
COS_9001AJ6RO(0/11.5)
F51TW9001BKOAN(10/7)
m1111(5/7)
m494(0/11)
m638(0/12)

## Slide 11
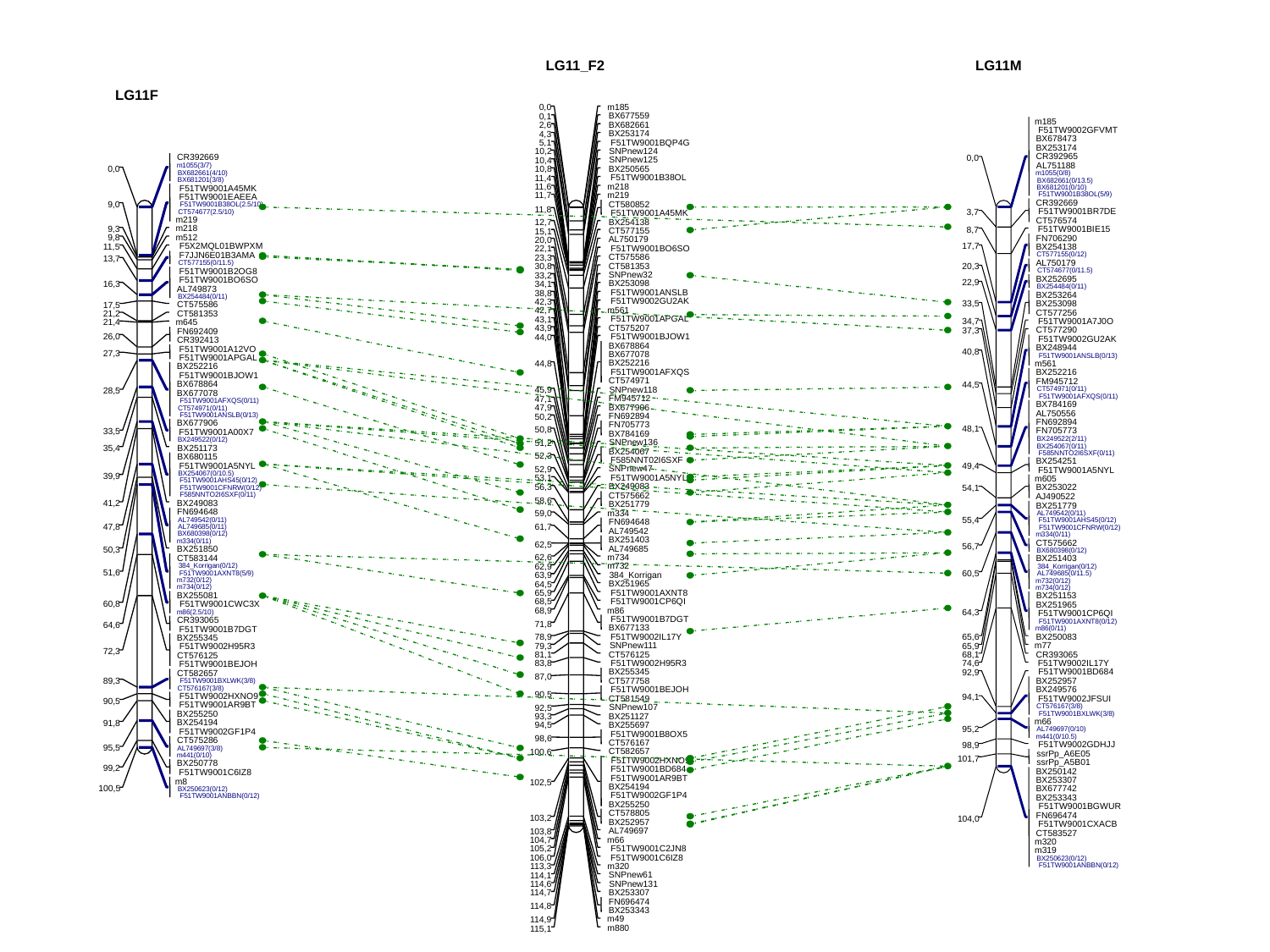

LG11_F2
m185
0,0
BX677559
0,1
BX682661
2,6
BX253174
4,3
F51TW9001BQP4G
5,1
SNPnew124
10,2
SNPnew125
10,4
BX250565
10,8
F51TW9001B38OL
11,4
m218
11,6
m219
11,7
CT580852
11,8
F51TW9001A45MK
BX254138
12,7
CT577155
15,1
AL750179
20,0
F51TW9001BO6SO
22,1
CT575586
23,3
CT581353
30,8
SNPnew32
33,2
BX253098
34,1
F51TW9001ANSLB
38,8
F51TW9002GU2AK
42,3
m561
42,7
F51TW9001APGAL
43,1
CT575207
43,9
F51TW9001BJOW1
44,0
BX678864
BX677078
BX252216
44,8
F51TW9001AFXQS
CT574971
SNPnew118
45,9
FM945712
47,1
BX677906
47,9
FN692894
50,2
FN705773
50,8
BX784169
SNPnew136
51,2
BX254067
52,3
F585NNT02I6SXF
SNPnew47
52,9
F51TW9001A5NYL
53,1
BX249083
56,3
CT575662
58,6
BX251779
m334
59,0
FN694648
61,7
AL749542
BX251403
62,5
AL749685
m734
62,6
m732
62,9
384_Korrigan
63,9
BX251965
64,5
F51TW9001AXNT8
65,9
F51TW9001CP6QI
68,5
m86
68,9
F51TW9001B7DGT
71,8
BX677133
F51TW9002IL17Y
78,9
SNPnew111
79,3
CT576125
81,1
F51TW9002H95R3
83,8
BX255345
87,0
CT577758
F51TW9001BEJOH
90,5
CT581549
SNPnew107
92,5
BX251127
93,3
BX255697
94,5
F51TW9001B8OX5
98,6
CT576167
CT582657
100,6
F51TW9002HXNO9
F51TW9001BD684
F51TW9001AR9BT
102,5
BX254194
F51TW9002GF1P4
BX255250
CT578805
103,2
BX252957
AL749697
103,8
m66
104,7
F51TW9001C2JN8
105,2
F51TW9001C6IZ8
106,0
m320
113,3
SNPnew61
114,1
SNPnew131
114,6
BX253307
114,7
FN696474
114,8
BX253343
m49
114,9
m880
115,1
LG11M
m185
F51TW9002GFVMT
BX678473
BX253174
CR392965
0,0
AL751188
m1055(0/8)
BX682661(0/13.5)
BX681201(0/10)
F51TW9001B38OL(5/9)
CR392669
F51TW9001BR7DE
3,7
CT576574
F51TW9001BIE15
8,7
FN706290
17,7
BX254138
CT577155(0/12)
AL750179
20,3
CT574677(0/11.5)
BX252695
22,9
BX254484(0/11)
BX253264
BX253098
33,5
CT577256
F51TW9001A7J0O
34,7
CT577290
37,3
F51TW9002GU2AK
BX248944
40,8
F51TW9001ANSLB(0/13)
m561
BX252216
FM945712
44,5
CT574971(0/11)
F51TW9001AFXQS(0/11)
BX784169
AL750556
FN692894
48,1
FN705773
BX249522(2/11)
BX254067(0/11)
F585NNTO2I6SXF(0/11)
BX254251
49,4
F51TW9001A5NYL
m605
BX253022
54,1
AJ490522
BX251779
AL749542(0/11)
55,4
F51TW9001AHS45(0/12)
F51TW9001CFNRW(0/12)
m334(0/11)
CT575662
56,7
BX680398(0/12)
BX251403
384_Korrigan(0/12)
60,5
AL749685(0/11.5)
m732(0/12)
m734(0/12)
BX251153
BX251965
64,3
F51TW9001CP6QI
F51TW9001AXNT8(0/12)
m86(0/11)
BX250083
65,6
m77
65,9
CR393065
68,1
F51TW9002IL17Y
74,6
F51TW9001BD684
92,9
BX252957
BX249576
94,1
F51TW9002JFSUI
CT576167(3/8)
F51TW9001BXLWK(3/8)
m66
95,2
AL749697(0/10)
m441(0/10.5)
F51TW9002GDHJJ
98,9
ssrPp_A6E05
101,7
ssrPp_A5B01
BX250142
BX253307
BX677742
BX253343
F51TW9001BGWUR
FN696474
104,0
F51TW9001CXACB
CT583527
m320
m319
BX250623(0/12)
F51TW9001ANBBN(0/12)
LG11F
CR392669
m1055(3/7)
0,0
BX682661(4/10)
BX681201(3/8)
F51TW9001A45MK
F51TW9001EAEEA
9,0
F51TW9001B38OL(2.5/10)
CT574677(2.5/10)
m219
m218
9,3
m512
9,8
F5X2MQL01BWPXM
11,5
F7JJN6E01B3AMA
13,7
CT577155(0/11.5)
F51TW9001B2OG8
F51TW9001BO6SO
16,3
AL749873
BX254484(0/11)
CT575586
17,5
CT581353
21,2
m645
21,4
FN692409
26,0
CR392413
F51TW9001A12VO
27,3
F51TW9001APGAL
BX252216
F51TW9001BJOW1
BX678864
28,5
BX677078
F51TW9001AFXQS(0/11)
CT574971(0/11)
F51TW9001ANSLB(0/13)
BX677906
33,5
F51TW9001A00X7
BX249522(0/12)
BX251173
35,4
BX680115
F51TW9001A5NYL
BX254067(0/10.5)
39,9
F51TW9001AHS45(0/12)
F51TW9001CFNRW(0/12)
F585NNTO2I6SXF(0/11)
BX249083
41,2
FN694648
AL749542(0/11)
47,8
AL749685(0/11)
BX680398(0/12)
m334(0/11)
BX251850
50,3
CT583144
384_Korrigan(0/12)
51,6
F51TW9001AXNT8(5/9)
m732(0/12)
m734(0/12)
BX255081
60,8
F51TW9001CWC3X
m86(2.5/10)
CR393065
64,6
F51TW9001B7DGT
BX255345
F51TW9002H95R3
72,3
CT576125
F51TW9001BEJOH
CT582657
89,3
F51TW9001BXLWK(3/8)
CT576167(3/8)
F51TW9002HXNO9
90,5
F51TW9001AR9BT
BX255250
BX254194
91,8
F51TW9002GF1P4
CT575286
95,5
AL749697(3/8)
m441(0/10)
BX250778
99,2
F51TW9001C6IZ8
m8
100,5
BX250623(0/12)
F51TW9001ANBBN(0/12)

## Slide 12
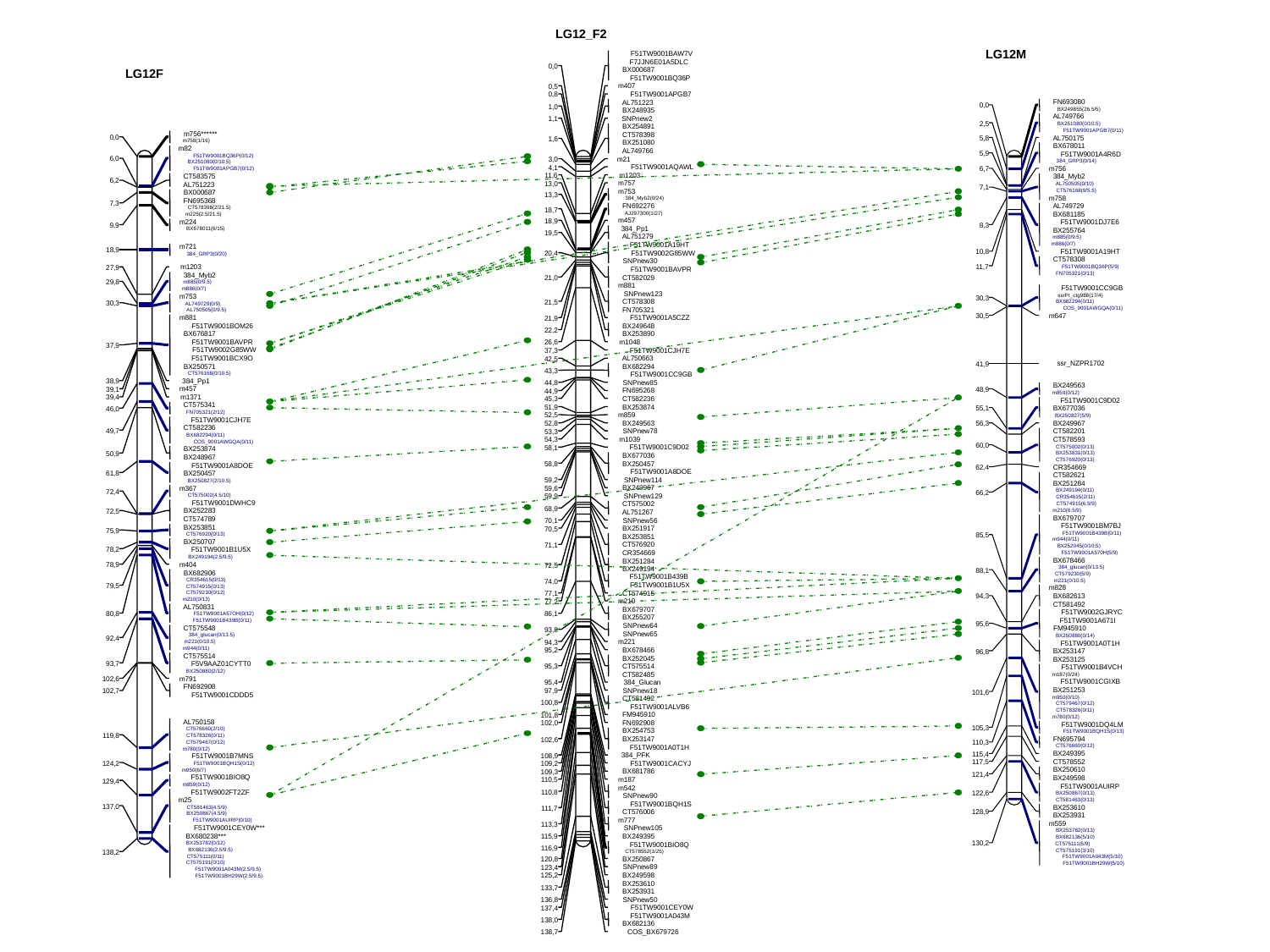

LG12_F2
F51TW9001BAW7V
F7JJN6E01A5DLC
0,0
BX000687
F51TW9001BQ36P
m407
0,5
F51TW9001APGB7
0,8
AL751223
1,0
BX248935
SNPnew2
1,1
BX254891
CT578398
1,6
BX251080
AL749766
m21
3,0
F51TW9001AQAWL
4,1
m1203
11,6
m757
13,0
m753
13,3
384_Myb2(0/24)
FN692276
18,7
AJ297300(1/27)
m457
18,9
384_Pp1
19,5
AL751279
F51TW9001A19HT
F51TW9002G85WW
20,4
SNPnew30
F51TW9001BAVPR
CT582029
21,0
m881
SNPnew123
CT578308
21,5
FN705321
F51TW9001A5CZZ
21,9
BX249648
22,2
BX253890
m1048
26,6
F51TW9001CJH7E
37,3
AL750663
42,5
BX682294
43,3
F51TW9001CC9GB
SNPnew85
44,8
FN695268
44,9
CT582236
45,3
BX253874
51,9
m859
52,5
BX249563
52,8
SNPnew78
53,3
m1039
54,3
F51TW9001C9D02
58,1
BX677036
BX250457
58,8
F51TW9001A8DOE
SNPnew114
59,2
BX249967
59,6
SNPnew129
59,9
CT575002
68,9
AL751267
SNPnew56
70,1
BX251917
70,5
BX253851
CT576920
71,1
CR354669
BX251284
BX249194
72,5
F51TW9001B439B
74,0
F51TW9001B1U5X
CT574915
77,1
m210
77,2
BX679707
86,1
BX255207
SNPnew64
93,8
SNPnew65
m221
94,3
BX678466
95,2
BX252045
CT575514
95,3
CT582485
384_Glucan
95,4
SNPnew18
97,9
CT581492
100,8
F51TW9001ALVB6
FM945910
101,8
FN692908
102,0
BX254753
BX253147
102,6
F51TW9001A0T1H
384_PFK
108,9
F51TW9001CACYJ
109,2
BX681786
109,3
m187
110,5
m542
110,8
SNPnew90
F51TW9001BQH1S
111,7
CT576006
m777
113,3
SNPnew105
BX249395
115,9
F51TW9001BIO8Q
116,9
CT578552(1/25)
BX250867
120,8
SNPnew89
123,4
BX249598
125,2
BX253610
133,7
BX253931
SNPnew50
136,8
F51TW9001CEY0W
137,4
F51TW9001A043M
138,0
BX682136
COS_BX679726
138,7
LG12M
FN693080
0,0
BX249855(26.5/5)
AL749766
2,5
BX251080(0/10.5)
F51TW9001APGB7(0/11)
AL750175
5,8
BX678011
5,9
F51TW9001A4R6D
384_GRP3(0/14)
m756
6,7
384_Myb2
AL750505(0/10)
7,1
CT576168(9/5.5)
m758
AL749729
BX681185
F51TW9001DJ7E6
8,3
BX255764
m885(0/9.5)
m886(0/7)
F51TW9001A19HT
10,8
CT578308
11,7
F51TW9001BQ36P(5/9)
FN705321(0/13)
F51TW9001CC9GB
ssrPt_ctg988(17/4)
30,3
BX682294(0/11)
COS_9001AWGQA(0/11)
m647
30,5
ssr_NZPR1702
41,9
BX249563
48,9
m859(0/12)
F51TW9001C9D02
55,1
BX677036
BX250827(5/9)
BX249967
56,3
CT582201
CT578593
60,0
CT575002(0/13)
BX253831(0/13)
CT576920(0/13)
CR354669
62,4
CT582621
BX251284
BX249194(0/11)
66,2
CR354615(2/11)
CT574915(6.5/9)
m210(6.5/9)
BX679707
F51TW9001BM7BJ
F51TW9001B439B(0/11)
85,5
m944(0/11)
BX252045(0/10.5)
F51TW9001A570H(5/9)
BX678466
384_glucan(0/13.5)
88,1
CT579230(5/9)
m221(0/10.5)
m828
BX682613
94,3
CT581492
F51TW9002GJRYC
F51TW9001A671I
95,6
FM945910
BX250880(0/14)
F51TW9001A0T1H
BX253147
96,8
BX253125
F51TW9001B4VCH
m187(0/24)
F51TW9001CGIXB
BX251253
101,6
m950(0/10)
CT579467(0/12)
CT578326(0/11)
m780(0/12)
F51TW9001DQ4LM
105,3
F51TW9001BQH1S(0/13)
FN695794
110,3
CT576660(0/12)
BX249395
115,4
CT578552
117,5
BX250610
121,4
BX249598
F51TW9001AUIRP
122,6
BX250867(0/13)
CT581463(0/13)
BX253610
128,9
BX253931
m559
BX253782(0/13)
BX682136(5/10)
130,2
CT575111(5/9)
CT575191(3/10)
F51TW9001A043M(5/10)
F51TW9001BH29W(5/10)
LG12F
m756******
0,0
m758(1/16)
m82
F51TW9001BQ36P(0/12)
6,0
BX251080(0/10.5)
F51TW9001APGB7(0/12)
CT583575
6,2
AL751223
BX000687
FN695368
7,3
CT578398(2/21.5)
m225(2.5/21.5)
m224
9,9
BX678011(6/15)
m721
18,9
384_GRP3(0/20)
m1203
27,9
384_Myb2
29,8
m885(0/9.5)
m886(0/7)
m753
30,3
AL749729(0/9)
AL750505(0/9.5)
m881
F51TW9001BOM26
BX676817
F51TW9001BAVPR
37,9
F51TW9002G85WW
F51TW9001BCX9O
BX250571
CT576168(0/10.5)
384_Pp1
38,9
m457
39,1
m1371
39,4
CT575341
46,0
FN705321(2/12)
F51TW9001CJH7E
CT582236
49,7
BX682294(0/11)
COS_9001AWGQA(0/11)
BX253874
50,9
BX248967
F51TW9001A8DOE
61,8
BX250457
BX250827(2/10.5)
m367
72,4
CT575002(4.5/10)
F51TW9001DWHC9
BX252283
72,5
CT574789
BX253851
75,9
CT576920(0/13)
BX250707
78,2
F51TW9001B1U5X
BX249194(2.5/9.5)
m404
78,9
BX682906
CR354615(0/13)
79,5
CT574915(0/13)
CT579230(0/12)
m210(0/13)
AL750831
80,8
F51TW9001A57OH(0/12)
F51TW9001B439B(0/11)
CT575548
384_glucan(0/13.5)
92,4
m221(0/10.5)
m944(0/11)
CT575514
93,7
F5V9AAZ01CYTT0
BX250880(2/12)
m791
102,6
FN692908
102,7
F51TW9001CDDD5
AL750158
CT576660(2/10)
119,8
CT578326(0/11)
CT579467(0/12)
m780(0/12)
F51TW9001B7MNS
124,2
F51TW9001BQH1S(0/12)
m950(6/7)
F51TW9001BIO8Q
129,4
m859(0/12)
F51TW9002FT2ZF
m25
137,0
CT581463(4.5/9)
BX250867(4.5/9)
F51TW9001AUIRP(0/10)
F51TW9001CEY0W***
BX680238***
BX253782(0/12)
BX682136(2.5/9.5)
138,2
CT575111(0/11)
CT575191(0/10)
F51TW9001A043M(2.5/9.5)
F51TW9001BH29W(2.5/9.5)
