# Supplementary material for: High-density linkage mapping in a pine tree reveals a genomic region associated with inbreeding depression and provides clues to the extent and distribution of meiotic recombination
Source: BMC Biol. 2013 Apr 18;11:50. doi: 10.1186/1741-7007-11-50 (PMC3660193; doi:10.1186/1741-7007-11-50)
Supplement: Additional file 4 — Segregation pattern in the F2 progeny before and after validation on a larger sample size. Only SNPs from the 12 k array were genotyped by the iPLEX Sequenom assay. [file 1741-7007-11-50-S4.doc]

**Additional file 4.** Segregation pattern in the F2 progeny before and after validation on a larger sample size. Only SNPs from the 12k assay were genotyped by the iPLEX Sequenom assay.

|  | SNP assay | Marker name | Genotypic proportions before validation: AA/AB/BB (sample size) | Genotypic proportions after iPLEX validation: AA/AB/BB (sample size) |
| --- | --- | --- | --- | --- |
| **Cluster#1 LG2** | 384 plex | m306 | 127/230/81 (438) **** |  |
|  | 384 plex | SNPnew128^1^ | 135/226/81 (442) **** |  |
|  | 384 plex | SNPnew127^1^ | 136/227/81 (444) ***** |  |
|  | 384 plex | SNPnew25 | 144/235/89 (468) **** |  |
|  | 12k plex | BX254087 | 22/40/6 (68) *** | 105/193/79 (377) not distorted |
|  |  |  |  |  |
| **Cluster#2 LG10** | 12k | FN256926 | 14/24/30 (68) **** | 98/172/103 (373) not distorted |
|  | 12k | CT580097 | 14/23/31 (68) ****** | Failed in iPLEX genotyping |
|  | 384plex | SNPnew94 | 115/190/139 (444) **** |  |
|  | 12k | FN256445 | 13/24/31 (68) ****** | 101/175/103 (379) not distorted |
|  | 12k | F51TW9001BKZIN | 13/24/31 (68) ****** | 100/172/107 (379) not distorted |
|  | 12k | BX251885 | 13/24/31 (68) ****** | Failed in iPLEX genotyping |
|  | 12k | F51TW9001B9TZM | 13/24/31 (68) ****** | 103/175/101 (379) not distorted |
|  | 12k | FN694683 | 13/24/31 (68) ****** | Failed in iPLEX genotyping |
|  | 12k | BX254530 | 13/24/31 (68) ****** | 101/177/101 (379) not distorted |
|  | 12k | AL750287 | 14/23/31 (68) ****** | 98/177/103 (378) not distorted |
|  | 12k | BX251885 | 13/24/31 (68) ****** | Failed in iPLEX genotyping |
|  |  |  |  |  |
| **Cluster#3 LG10** | 12k | CT575893 | 29/31/8 (68) **** | 101/189/89 (379) not distorted |
|  | 12k | F7JJN6E01BOY8E | 28/32/8 (68) **** | 100/186/92 (378) not distorted |
|  | 12k | BX253781 | 27/33/8 (68) **** | 97/191/91 (379) not distorted |
|  | 12k | F51TW9001EQO62 | 29/31/8 (68) **** | 98/192/89 (379) not distorted |
|  | 12k | BX252612 | 28/32/8 (68) **** | 95/187/95 (377) not distorted |
|  | 12k | COS_W9001AJ6RO | 28/32/8 (68) **** | 98/186/94 (378) not distorted |
|  | 12k | COS_W9001A7SWZ | 28/31/9 (68) **** | 98/189/92 (379) not distorted |
|  | 12k | BX253504 | 29/32/7 (68) ***** | 93/190/94 (377) not distorted |
|  | 12k | F51TW9001B0OJP | 29/32/7 (68) ***** | Failed in iPLEX genotyping |

^1^ : SNPs on the same contig
