# Supplementary material for: High-density linkage mapping in a pine tree reveals a genomic region associated with inbreeding depression and provides clues to the extent and distribution of meiotic recombination
Source: BMC Biol. 2013 Apr 18;11:50. doi: 10.1186/1741-7007-11-50 (PMC3660193; doi:10.1186/1741-7007-11-50)
Supplement: Additional file 5 — Analysis of allele transmission and segregation distortion in the F2 pedigree. Alleles inherited from the Corsican grandparent are highlighted in green. [file 1741-7007-11-50-S5.docx]

**Additional file 5.** Analysis of allele transmission and segregation distortion in the F2 pedigree. Alleles inherited from the Corsican grandparent are highlighted in green.

| Distorted markers | L146 x C10 | H12 | Number of AA observed /**expected** | Number of AB observed /**expected** | Number of BB observed /**expected** |
| --- | --- | --- | --- | --- | --- |
| m306 | AB x BB | AB | 127 / **109.5** | 230 / 219 | 81 / **109.5** |
| SNPnew127 | AA x BB | AB | 136 / **111** | 227 / 222 | 81 / **111** |
| SNPnew128 | BB x AA | AB | 81 / **110.5** | 226 / 221 | 135 / **110.5** |
| SNPnew25 | BB x AA | AB | 89 / **117** | 235 / 234 | 144 / **117** |
