# Supplementary material for: High-density linkage mapping in a pine tree reveals a genomic region associated with inbreeding depression and provides clues to the extent and distribution of meiotic recombination
Source: BMC Biol. 2013 Apr 18;11:50. doi: 10.1186/1741-7007-11-50 (PMC3660193; doi:10.1186/1741-7007-11-50)
Supplement: Additional file 6 — Summary of polymorphic and mapped markers on map 1 and map 3 for the G2F, G2M and F2 linkage maps. [file 1741-7007-11-50-S6.doc]

**Additional file 6**: Summary of polymorphic and mapped markers on map 1 and map 3 for the G2F, G2M and F2 linkage maps.

|  |  | **G2F** | **G2M** |  |  |  | **F2** |
| --- | --- | --- | --- | --- | --- | --- | --- |
| **1:1 Polymorphic markers** | 12k SNP Infinium assay | 760 SNPs / 543 loci | 900 SNPs/ 615 loci |  | **1:2:1 Polymorphic markers** | 12k SNP Infinium assay | 1,215 SNPs / 881 loci |
|  | 1,536 SNP Goldengate assay | 299 SNPs / 239 loci | |  |  | 1,536 SNP Goldengate assay | 193 SNPs / 172 loci |
|  | SSRs | 17 loci | 20 loci |  |  | 384 SNP Goldengate assay | 137 SNPs/ 124 loci |
|  | EST-Ps | 30 loci | 18 loci |  |  |  |  |
|  |  |  |  |  | **1:2:1 Framework mapped markers (map1)** | 12k SNP Infinium | 865 loci |
| **1:1 Framework mapped markers (map1)** | 12k SNP Infinium assay | 442 loci | 500 loci |  |  | 1,536 SNP Goldengate assay | 130 loci |
|  | 1,536 SNP Goldengate assay | 78 loci | 93 loci |  |  | 384 SNP Veracode assay | 126 loci |
|  | SSRs | 11 loci | 14 loci |  |  | TOTAL | 1,121 loci |
|  | EST-Ps | 19 loci | 12 loci |  |  | Common G2F/F2 | 198 loci |
|  | TOTAL | 550 loci | 619 loci |  |  | Common G2M/F2 | 240 loci |
|  | Common markers | 25 loci | |  |  |  |  |
|  |  |  |  |  | **1:2:1 Accessory mapped markers (map3)** | 12k SNP Infinium assay | 7 loci |
| **1:1 Accessory mapped markers (map3)** | 12k SNP Infinium assay | 42 loci | 65 loci |  |  | 1,536 SNP Goldengate assay | 2 loci |
|  | 1,536 SNP Goldengate assay | 10 loci | 15 loci |  |  | 384 SNP Veracode assay | 1 gene |
|  | SSRs | 0 loci | 1 loci |  |  | TOTAL | 10 loci |
|  | EST-Ps | 4 loci | 1 gene |  |  | Common G2F/F2 | 0 gene |
|  | TOTAL | 56 loci | 82 loci |  |  | Common G2M/F2 | 3 loci |
|  | Common G2F/G2M | 2 loci | |  |  |  |  |

| **1:2:1 Accessory mapped markers** | 12k SNP Infinium assay | 341 loci |
| --- | --- | --- |
|  | 1,536 SNP Goldengate assay | 64 loci |
|  | SSRs | 0 loci |
|  | EST-Ps | 4 loci |
|  | TOTAL | 409 loci |
