# Supplementary material for: High-density linkage mapping in a pine tree reveals a genomic region associated with inbreeding depression and provides clues to the extent and distribution of meiotic recombination
Source: BMC Biol. 2013 Apr 18;11:50. doi: 10.1186/1741-7007-11-50 (PMC3660193; doi:10.1186/1741-7007-11-50)
Supplement: Additional file 7 — Map length and number of markers for the three maps: G2F, G2M and F2. [file 1741-7007-11-50-S7.doc]

**Additional file 7.** Map length and number of markers for the three maps: G2F, G2M and F2.

| G2F | LG1F | LG2F | LG3F | LG4F | LG5F | LG6F | LG7F | LG8F | LG9F | LG10F | LG11F | LG12F | total |
| --- | --- | --- | --- | --- | --- | --- | --- | --- | --- | --- | --- | --- | --- |
| Length of map1 (cM) | 124 | 131 | 129 | 105 | 136 | 110 | 121 | 115 | 109 | 128 | 138 | 100.5 | 1,447 |
| Nb of markers in map1 | 48 | 62 | 49 | 37 | 33 | 60 | 37 | 41 | 42 | 41 | 51 | 49 | 550 |
| 12k SNP array | 40 | 48 | 38 | 31 | 25 | 42 | 29 | 34 | 38 | 35 | 38 | 44 | 442 |
| 1,536 SNP array | 5 | 12 | 6 | 4 | 4 | 11 | 6 | 4 | 4 | 4 | 13 | 5 | 78 |
| SSR/EST | 3 | 2 | 4 | 2 | 4 | 7 | 2 | 3 | 0 | 2 | 0 | 0 | 29 |
| Nb of additional markers in map3 (1:1) | 0 | 1 | 6 | 8 | 3 | 3 | 21 | 0 | 5 | 4 | 5 | 0 | 56 |
| Nb of additional markers in map3 (1:2:1) | 39 | 33 | 34 | 31 | 33 | 36 | 18 | 41 | 40 | 33 | 42 | 29 | 409 |
| Total number of markers | 87 | 96 | 89 | 76 | 69 | 99 | 76 | 82 | 87 | 78 | 98 | 78 | 1,015 |
| **G2M** | **LG1M** | **LG2M** | **LG3M** | **LG4M** | **LG5M** | **LG6M** | **LG7M** | **LG8M** | **LG9M** | **LG10M** | **LG11M** | **LG12M** | **total** |
| Length of map1 (cM) | 116 | 152 | 134 | 121 | 127 | 115 | 80 | 119 | 106 | 121 | 130 | 104 | 1,425 |
| Nb of markers in map1 | 47 | 49 | 57 | 48 | 57 | 52 | 44 | 42 | 49 | 62 | 50 | 62 | 619 |
| 12k SNP array | 39 | 37 | 41 | 38 | 41 | 46 | 38 | 35 | 39 | 49 | 44 | 52 | 499 |
| 1,536 SNP array | 6 | 10 | 11 | 6 | 12 | 4 | 5 | 7 | 9 | 11 | 5 | 7 | 93 |
| SSRs/ESTs | 2 | 2 | 4 | 4 | 4 | 2 | 1 | 0 | 1 | 2 | 1 | 3 | 26 |
| Nb of additional markers in map3 (1:1) | 0 | 0 | 7 | 3 | 12 | 7 | 23 | 11 | 15 | 0 | 4 | 0 | 82 |
| Nb of additional markers in map3 (1:2:1) | 39 | 33 | 34 | 31 | 33 | 36 | 18 | 41 | 40 | 33 | 42 | 29 | 409 |
| Total number of markers | 86 | 82 | 97 | 82 | 102 | 95 | 85 | 94 | 104 | 95 | 96 | 91 | 1,110 |
| **F2** | **LG1** | **LG2** | **LG3** | **LG4** | **LG5** | **LG6** | **LG7** | **LG8*** | **LG9** | **LG10** | **LG11** | **LG12** | **Total** |
| Length of map1 (cM) | 126 | 137.5 | 162.5 | 138 | 155 | 125.5 | 142 | 183 | 136 | 148 | 115 | 139 | 1,708 |
| Nb of markers in map1 | 69 | 90 | 122 | 99 | 92 | 95 | 97 | 70 | 98 | 88 | 94 | 107 | 1,121 |
| 12k SNP array | 57 | 68 | 86 | 84 | 72 | 71 | 75 | 57 | 77 | 68 | 69 | 77 | 861 |
| 1,536 SNP array | 5 | 12 | 17 | 11 | 16 | 12 | 14 | 2 | 12 | 5 | 15 | 15 | 136 |
| 384 SNP assay | 7 | 10 | 19 | 4 | 4 | 12 | 8 | 11 | 9 | 15 | 10 | 15 | 124 |
| Nb of additional markers in map3 (1:2:1) | 3 | 0 | 0 | 0 | 3 | 0 | 1 | 0 | 0 | 0 | 0 | 3 | 10 |
| Total number of markers | 72 | 90 | 122 | 99 | 95 | 95 | 98 | 70 | 98 | 88 | 94 | 110 | 1,131 |

*****LG8 was divided into 2 sub-groups of 36 and 97 cM, 50 cM was added to its length to take into account this gap.
