# Supplementary material for: High-density linkage mapping in a pine tree reveals a genomic region associated with inbreeding depression and provides clues to the extent and distribution of meiotic recombination
Source: BMC Biol. 2013 Apr 18;11:50. doi: 10.1186/1741-7007-11-50 (PMC3660193; doi:10.1186/1741-7007-11-50)
Supplement: Additional file 8 — Result of the Wilcoxon signed rank test (P-values) of pair-wise recombination for markers common to F2 and G2F and to F2 and G2M. [file 1741-7007-11-50-S8.doc]

**Additional file 8.** Result of the Wilcoxon signed rank test (*p*-values) of pair-wise recombination for markers common to F2 and G2F and to F2 and G2M.

|  |  | G2F | G2M |
| --- | --- | --- | --- |
| F2 | whole map | 4.4x10^-7^ | 2.2x10^-10^ |
|  | LG1 | ns | ns |
|  | LG2 | 7.8x10^-13^ | ns |
|  | LG3 | 6.9x10^-13^ | 1.3 x10^-31^ |
|  | LG4 | 4.6 x10^-6^ | 8.5 x10^-11^ |
|  | LG5 | ns | 2.8 x10^-12^ |
|  | LG6 | 2.2 x10^-8^ | 3.1 x10^-5^ |
|  | LG7 | 0.025 | 2.4 x10^-11^ |
|  | LG8 | 0.00065 | 8.2 x10^-7^ |
|  | LG9 | 4 x10^-12^ | 3.5 x10^-17^ |
|  | LG10 | 0.0024 | 0.03 |
|  | LG11 | 2 x10^-10^ | ns |
|  | LG12 | 0.007 | 2.4 x10^-10^ |
