# Supplementary material for: High-density linkage mapping in a pine tree reveals a genomic region associated with inbreeding depression and provides clues to the extent and distribution of meiotic recombination
Source: BMC Biol. 2013 Apr 18;11:50. doi: 10.1186/1741-7007-11-50 (PMC3660193; doi:10.1186/1741-7007-11-50)
Supplement: Additional file 9 — Distribution of the observed number of markers relative to the expected number of markers, assuming a Poisson distribution for the F2, G2F and G2M parental trees. Blocks with the same marker counts were summed and the resulting frequencies compared with the expected frequencies generated from the Poisson distribution function (indeed, if recombination on each chromosome were completely random, a Poisson distribution with a variance equal to the mean would be expected, as suggest by Haldane (1931). Haldane, J. B. S. (1931) The cytological basis of genetical interference. Cytologia 3:54–65). Black squares indicate the lower and upper thresholds (in terms of the number of markers per block) defining hotspots and coldspots of recombination, respectively. [file 1741-7007-11-50-S9.doc]

**Additional file 9**: Distribution of the observed number of markers relative to the expected number of markers, assuming a Poisson distribution for the F2, G2F and G2M parental trees. Blocks with the same marker counts were summed and the resulting frequencies compared with the expected frequencies generated from the Poisson distribution function (indeed, if recombination on each chromosome were completely random, a Poisson distribution with a variance equal to the mean would be expected, as suggest by Haldane (1931). *Haldane, J. B. S. (1931) The cytological basis of genetical interference. Cytologia 3: 54–65)*. Black squares indicate the lower and upper thresholds (in terms of the number of markers per block) defining hotspots and coldspots of recombination, respectively.

**Hot spot threshold**

**Cold spot threshold**
