# Supplementary material for: High-density linkage mapping in a pine tree reveals a genomic region associated with inbreeding depression and provides clues to the extent and distribution of meiotic recombination
Source: BMC Biol. 2013 Apr 18;11:50. doi: 10.1186/1741-7007-11-50 (PMC3660193; doi:10.1186/1741-7007-11-50)
Supplement: Additional file 10 — This supplementary data file contains three items. *A representation of marker density in the linkage groups of the G2F, G2M and F2 maps, highlighting coldspots and hotspots of recombination. Marker density was determined by shifting an interval along the map in 1 cM increments. The horizontal lines indicate the lower and upper thresholds defining gene clusters and gaps, respectively. x-axis: map distance for the whole linkage group (marker position as in Additional file 3, common markers are highlighted in green (between G2F and F2) and in pink (between G2M and F2), and markers common to G2F, G2M and F2 are enclosed in a box. y-axis: number of genes in the interval. Clusters common to the F2 map and at least one G2 map are indicated by orange circles connected by dotted orange lines. Clusters common to the G2F and G2M maps are indicated by black circles connected by dotted black lines. Clusters observed on only one map are indicated by black circles. *A table indicating the number of recombination hot- and coldspots on the G2F, G2M and F2 linkage maps. *A Venn diagram based on the table, to visualize the number of cold- (in black) and hotspots (in red) specific to a given map or common to different maps. [file 1741-7007-11-50-S10.doc]

**Additional file 9 :**  This supplementary data file contains three items.

*A representation of marker density in the linkage groups of the G2F, G2M and F2 maps, highlighting coldspots and hotspots of recombination. Marker density was determined by shifting an interval along the map in 1 cM increments. The horizontal lines indicate the lower and upper thresholds defining gene clusters and gaps, respectively. *x*-axis: map distance for the whole linkage group (marker position as in Additional file 3, common markers are highlighted in green (between G2F and F2) and in pink (between G2M and F2), and markers common to G2F, G2M and F2 are enclosed in a box. *y*-axis: number of genes in the interval. Clusters common to the F2 map and at least one G2 map are indicated by orange circles connected by dotted orange lines. Clusters common to the G2F and G2M maps are indicated by black circles connected by dotted black lines. Clusters observed on only one map are indicated by black circles.

*A table indicated the number of recombination hot- and coldspots on the G2F, G2M and F2 linkage maps.

*A Venn diagram based on the table, to visualize the number of cold- (in black) and hotspots (in red) specific to a given map or common to different maps.


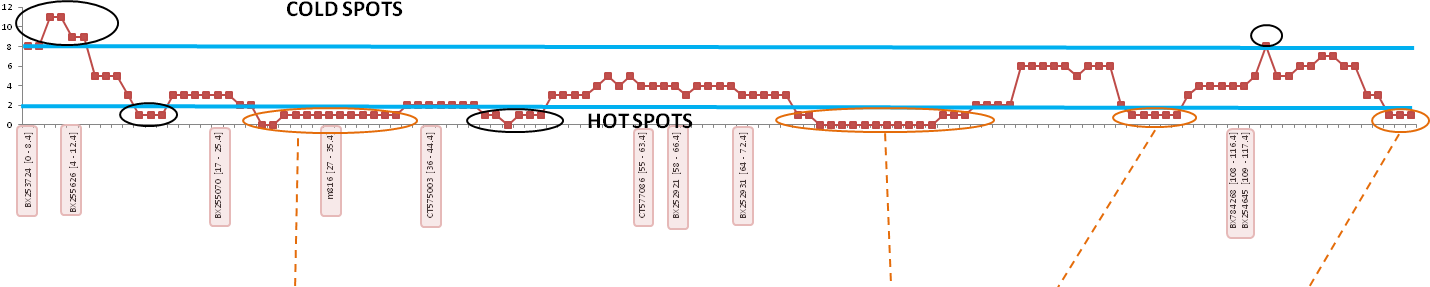


G2F

**LG1**


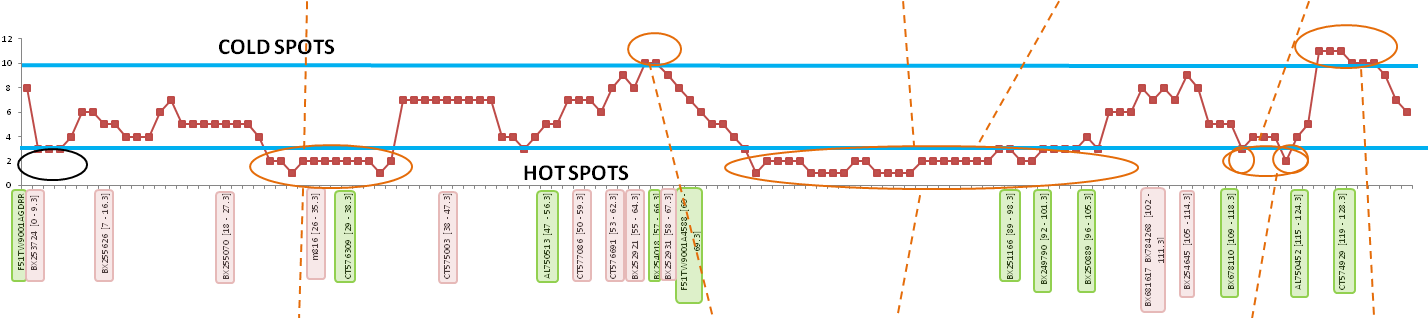


F2


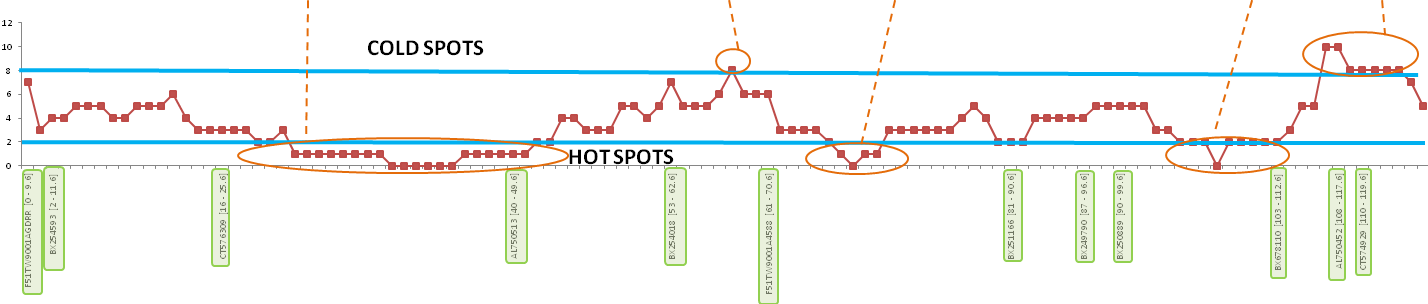


G2M

G2F

**LG2**


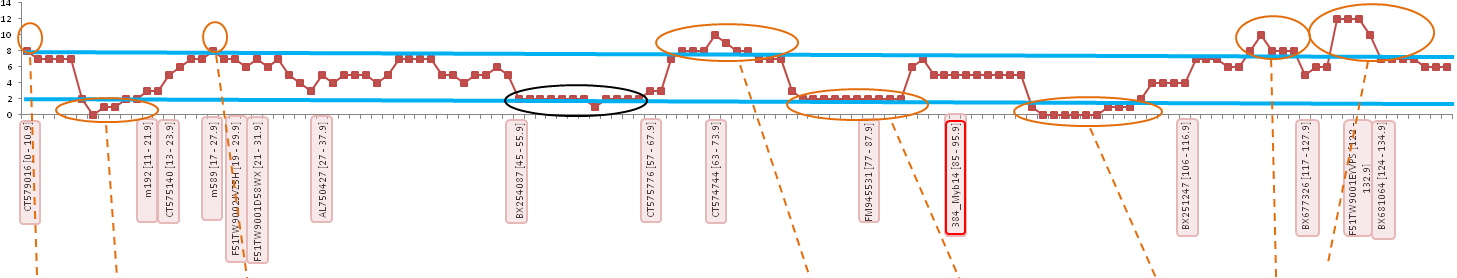


F2


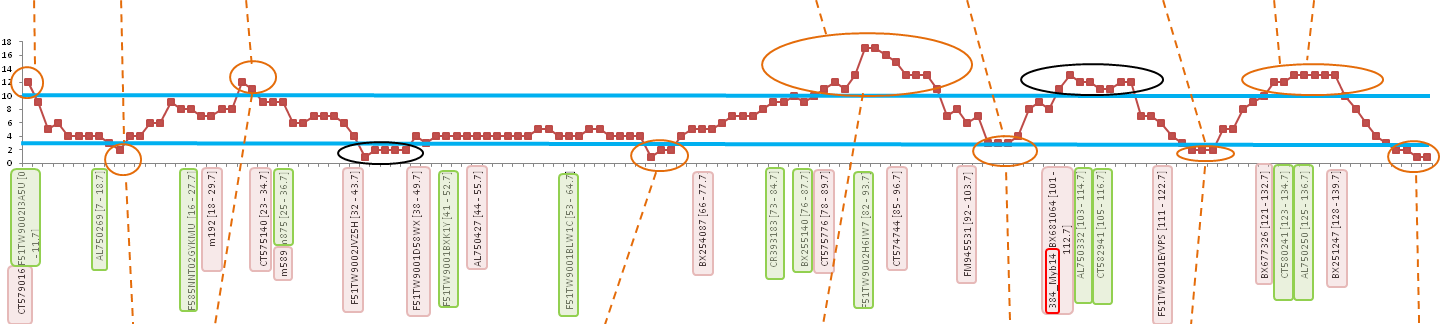


F2


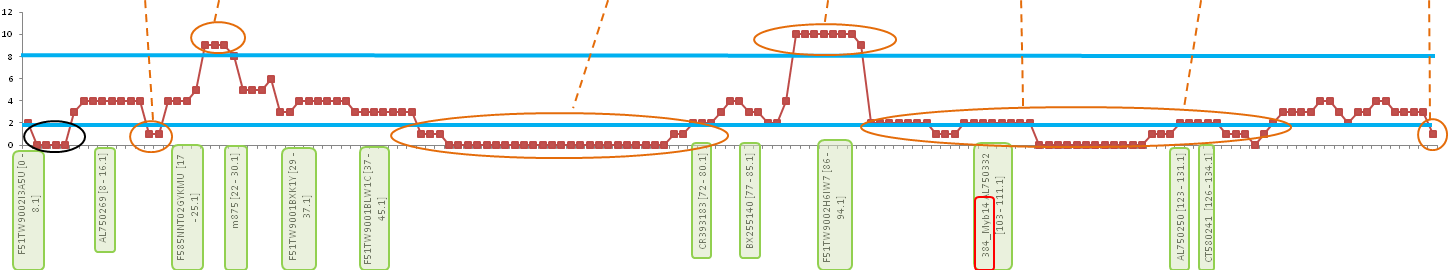


G2M


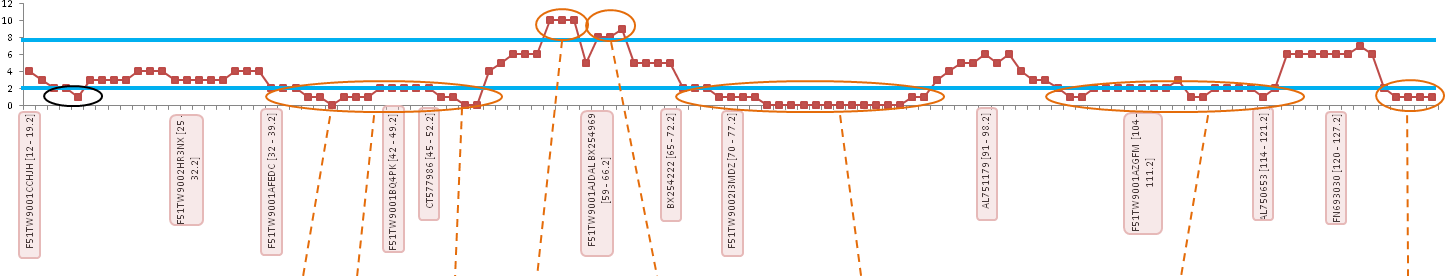


G2F

**LG3**


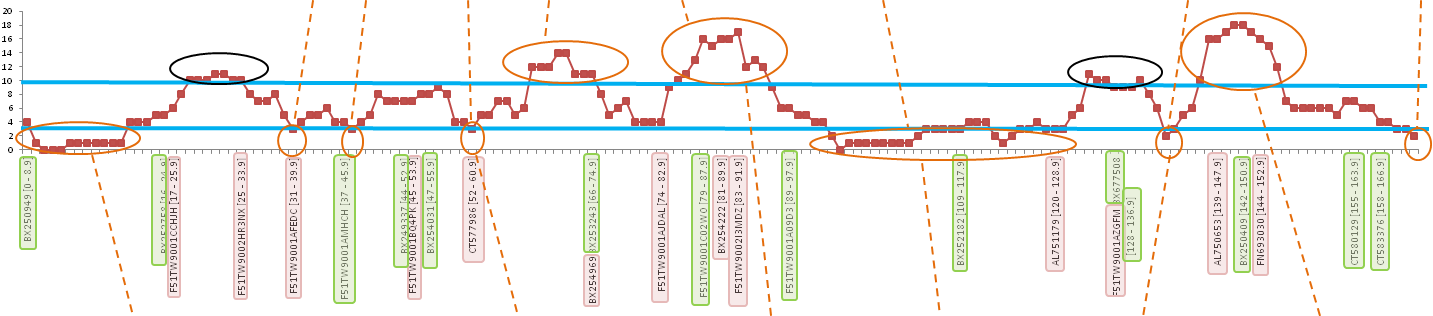


F2


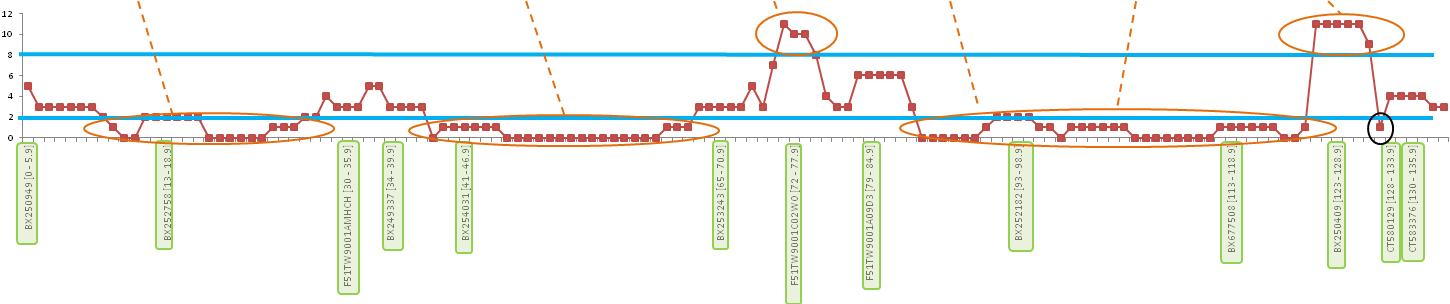


G2M

**
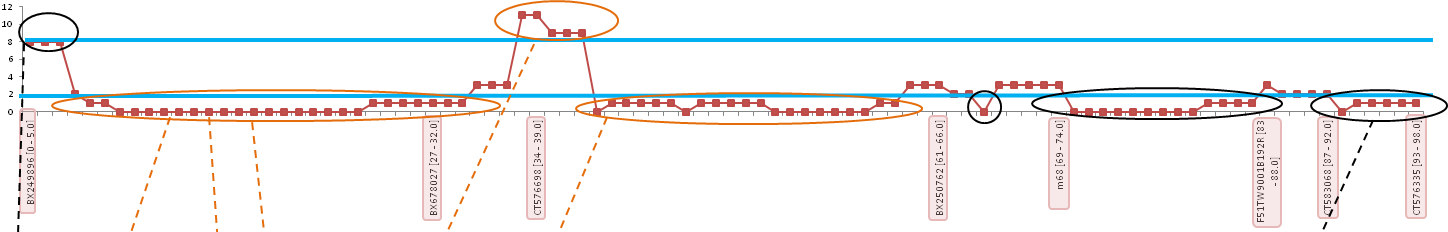
**

G2F

**LG4**


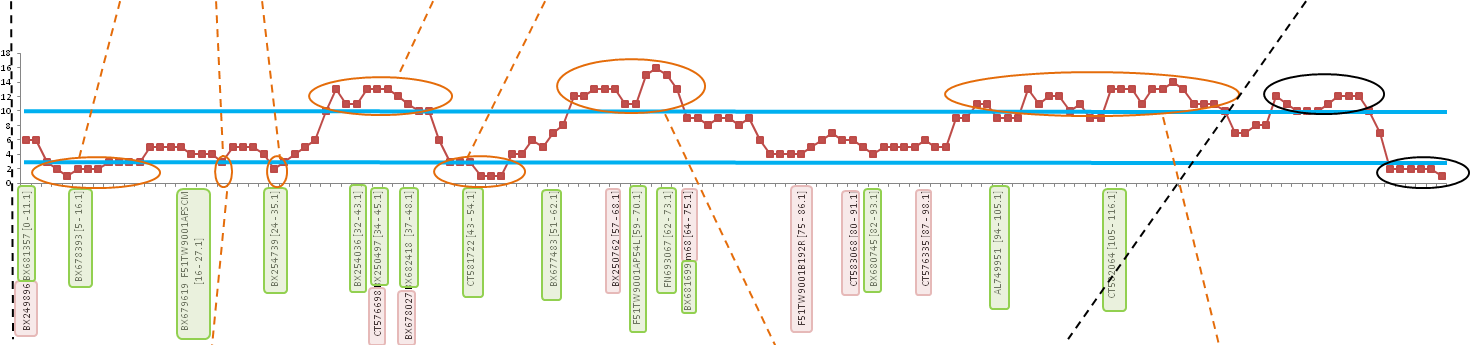


F2


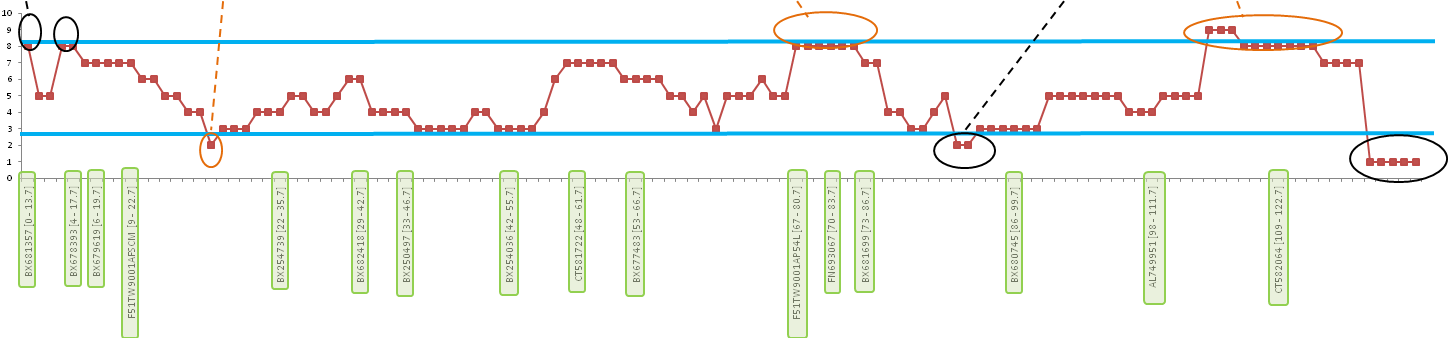


G2M


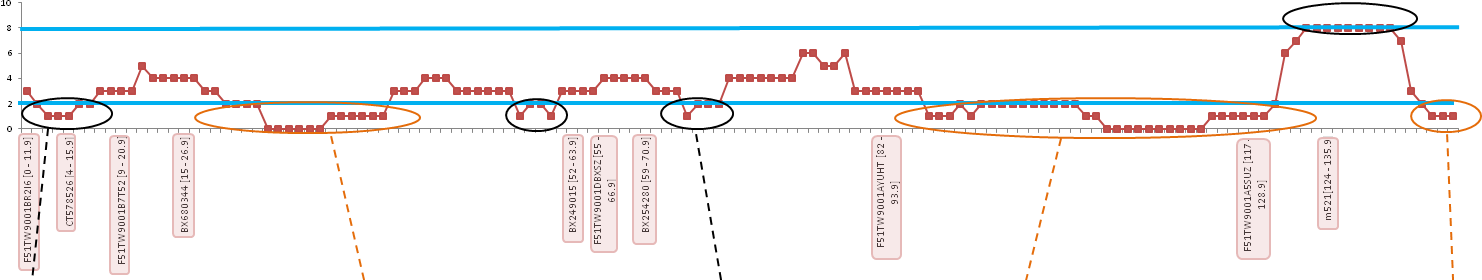


G2F

F2

**LG5**


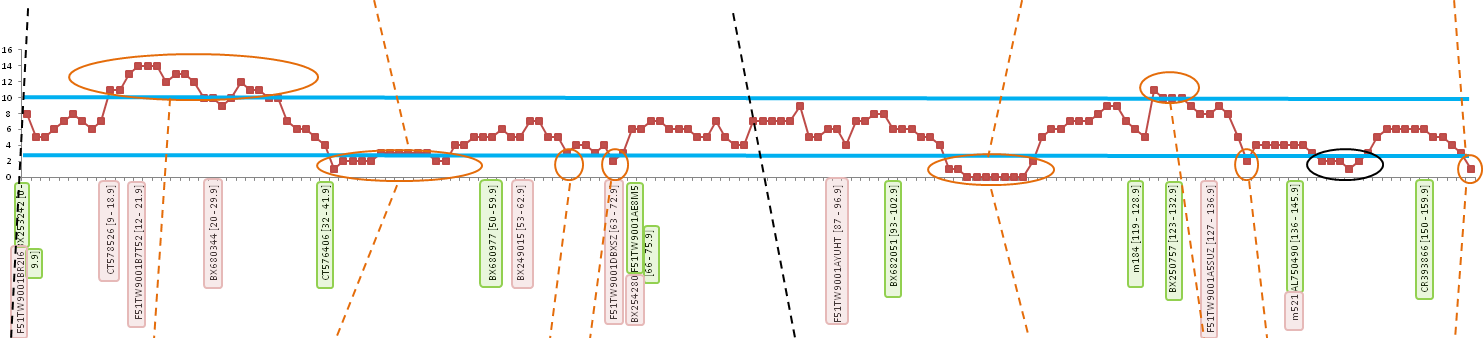


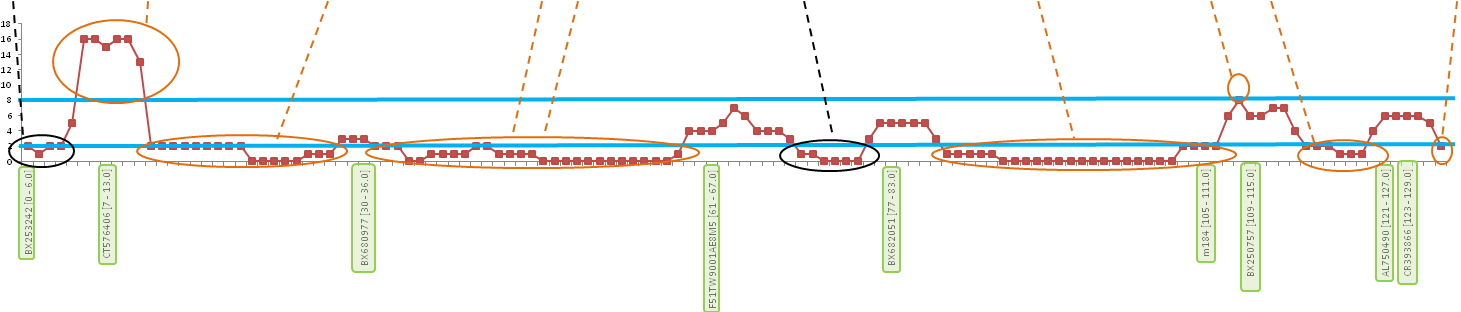


G2M


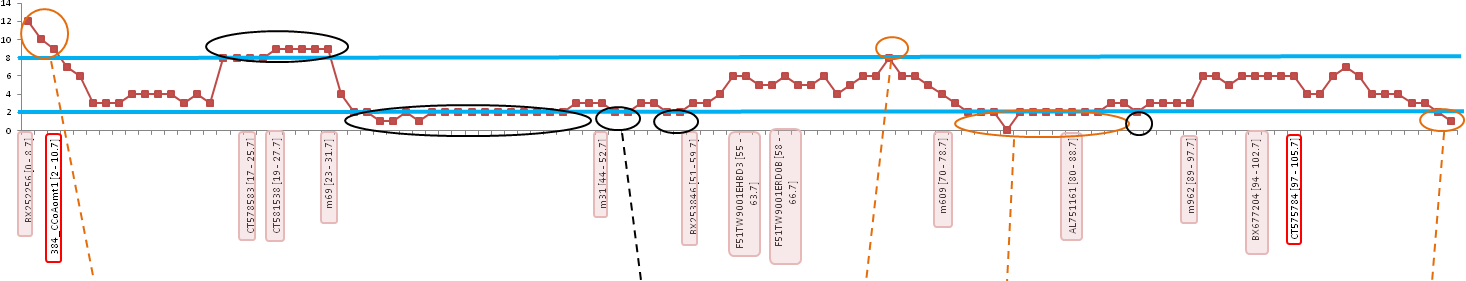


G2F

**LG6**


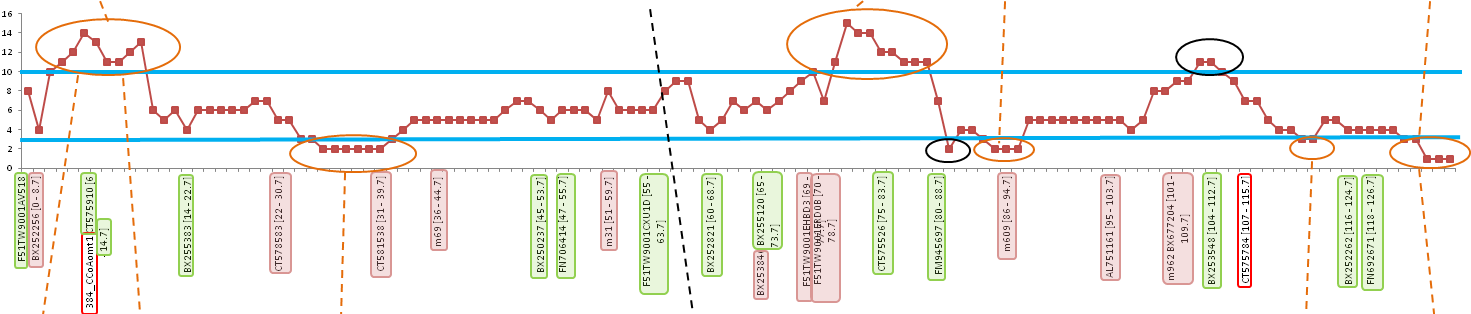


F2


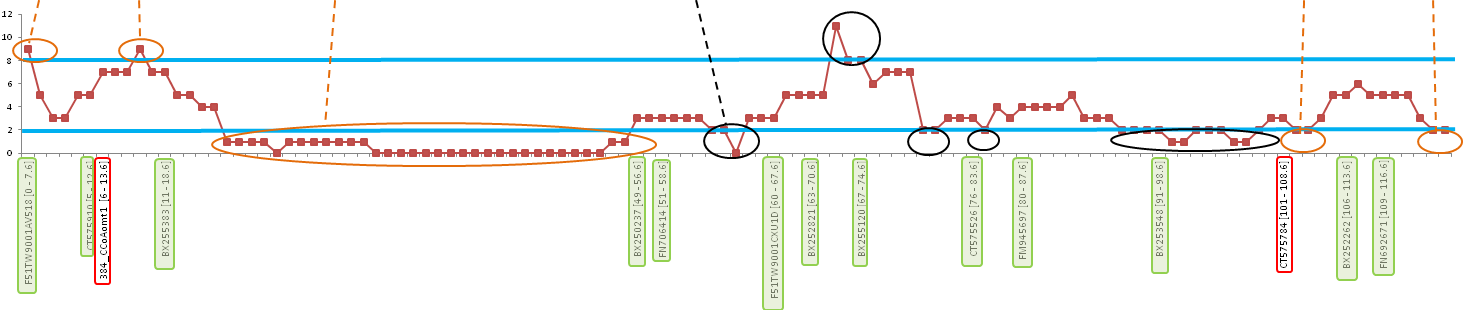


G2M


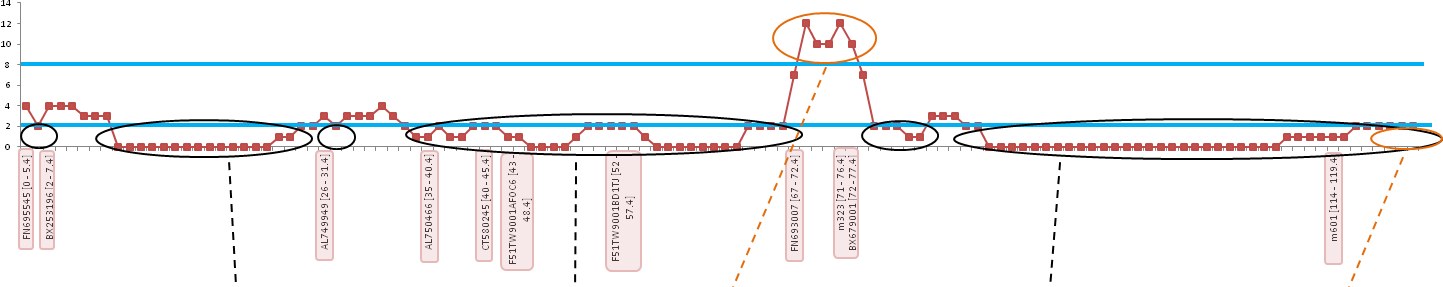


G2F

**LG7**


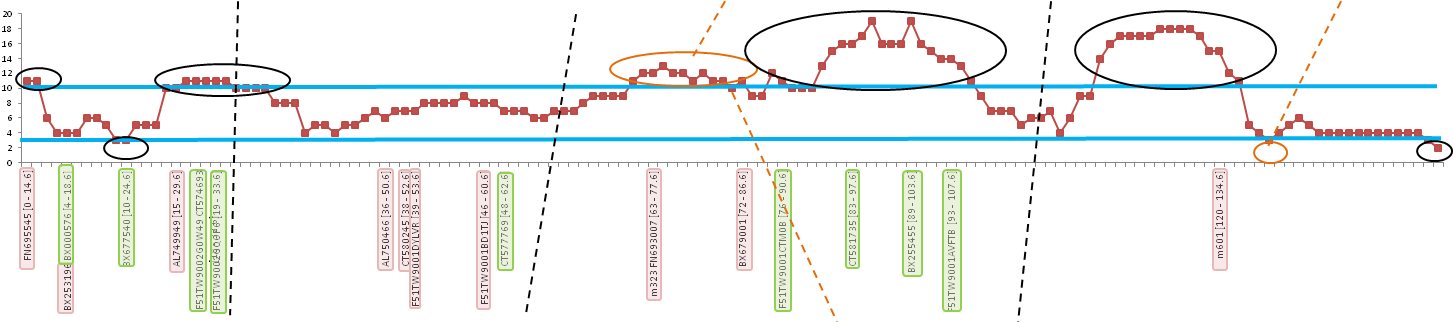


F2


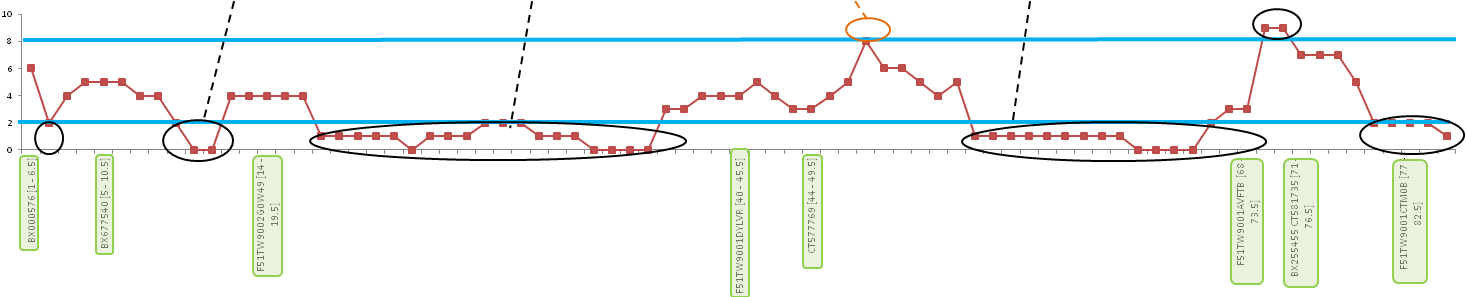


G2M


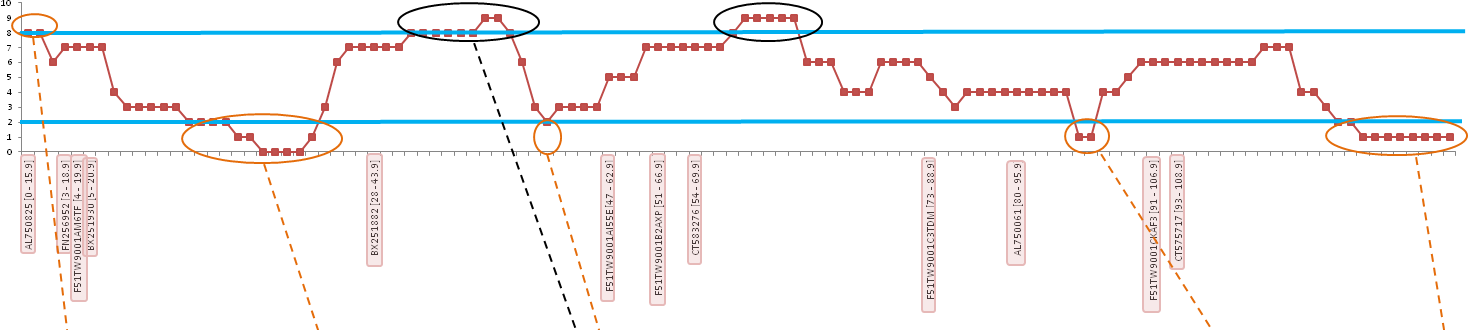

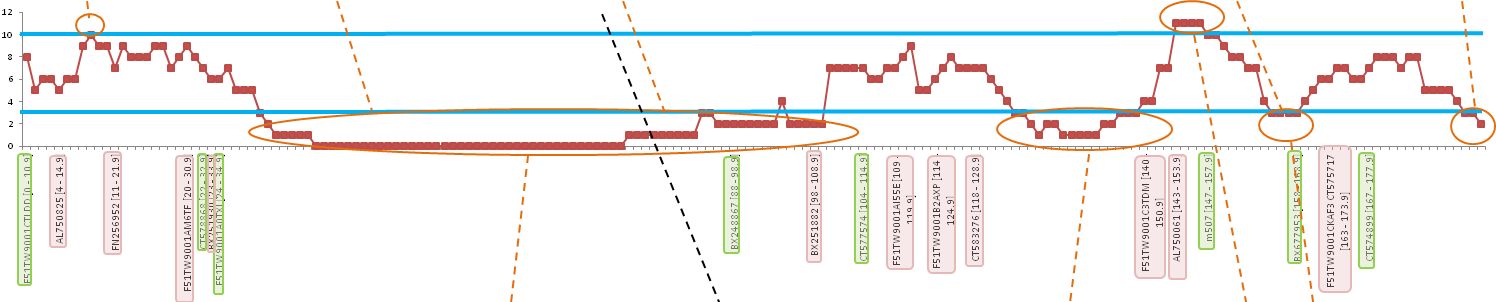


G2F

F2

**LG8**


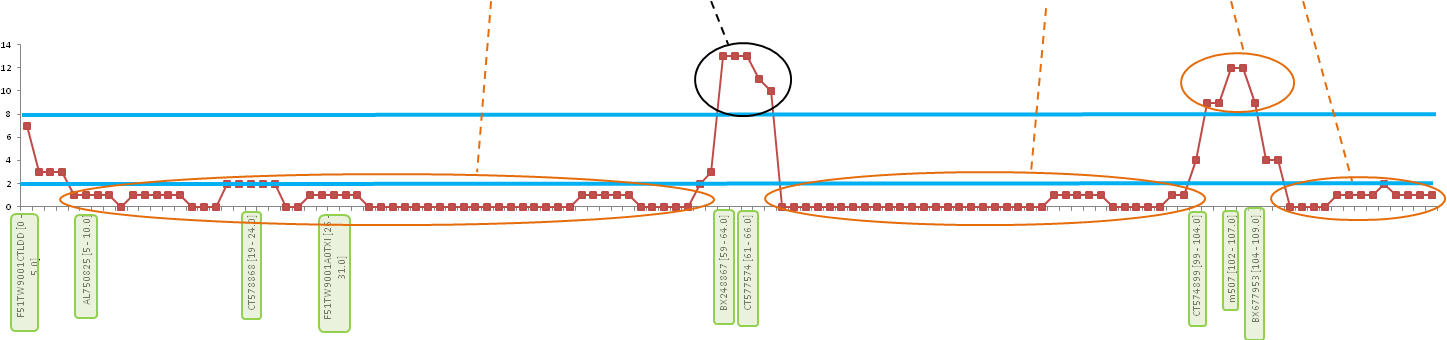


G2M


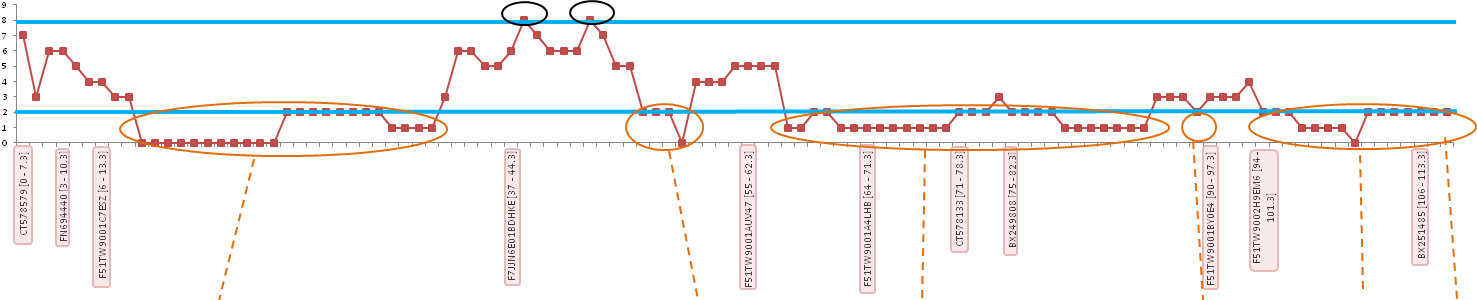


G2F

**LG9**


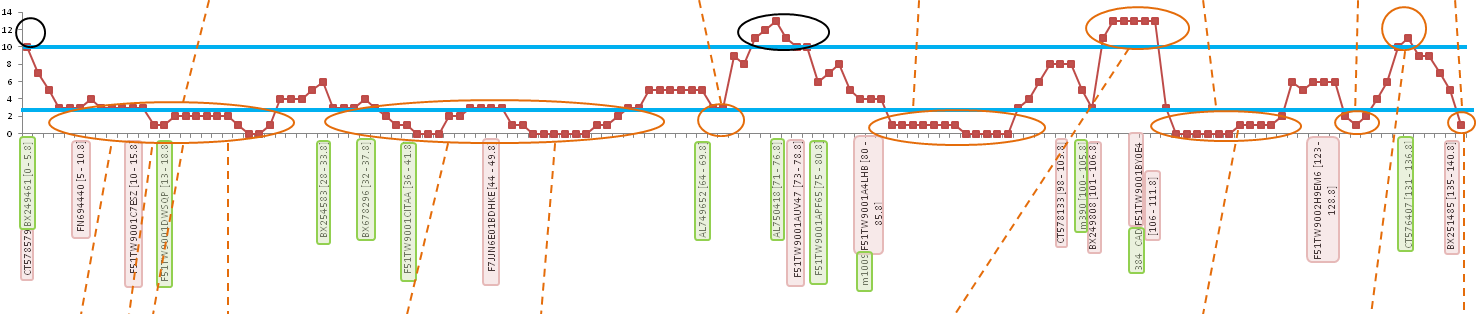


F2


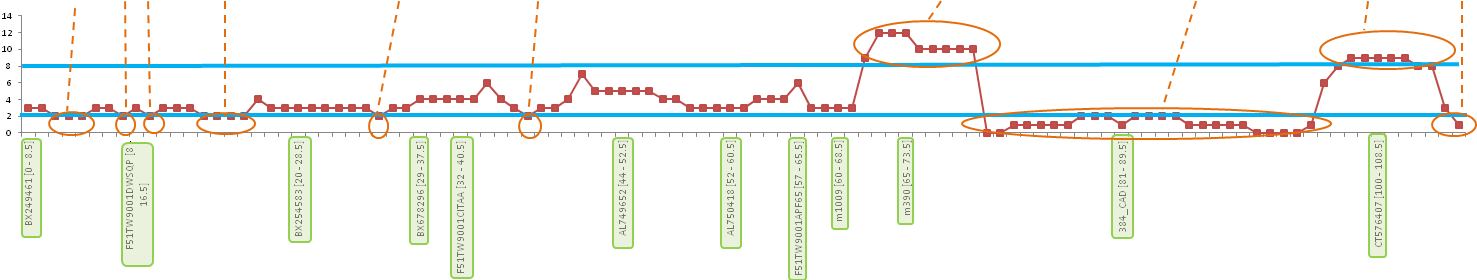


G2M


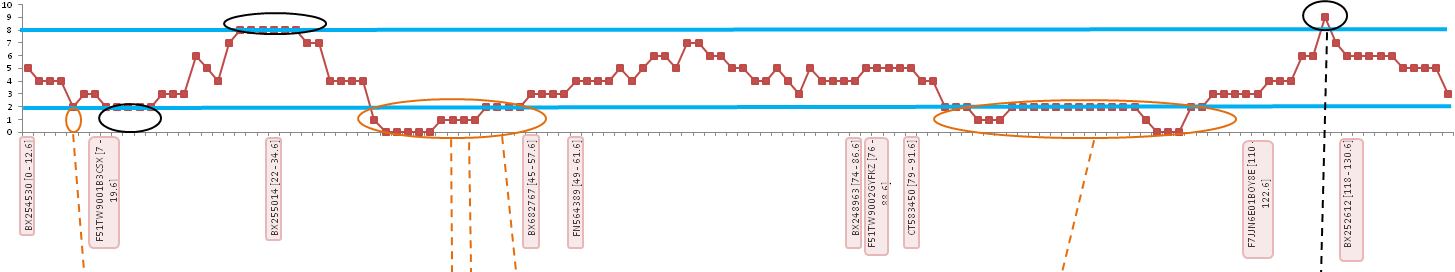


G2F

**LG10**


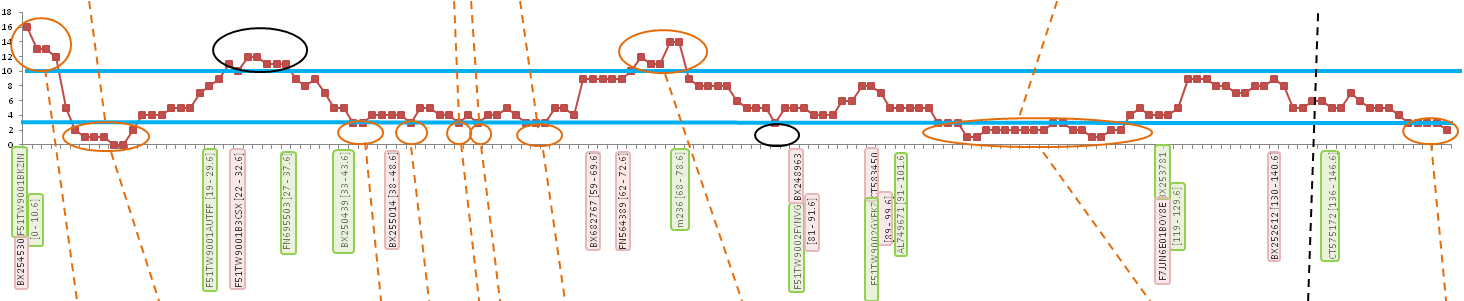


F2


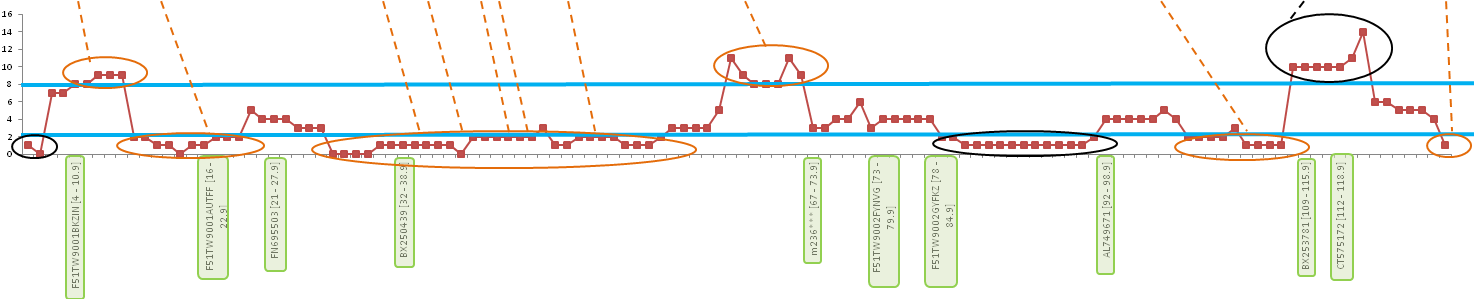


G2M


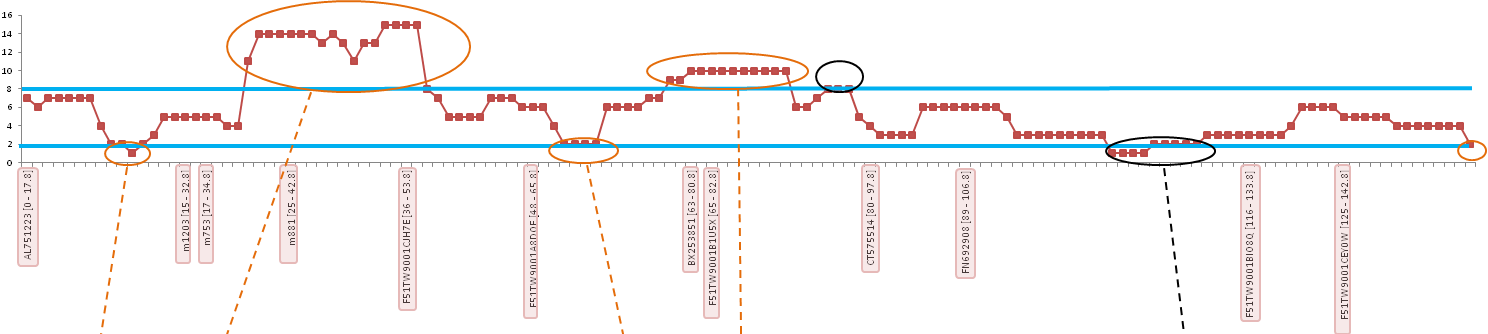


G2F

**LG11**


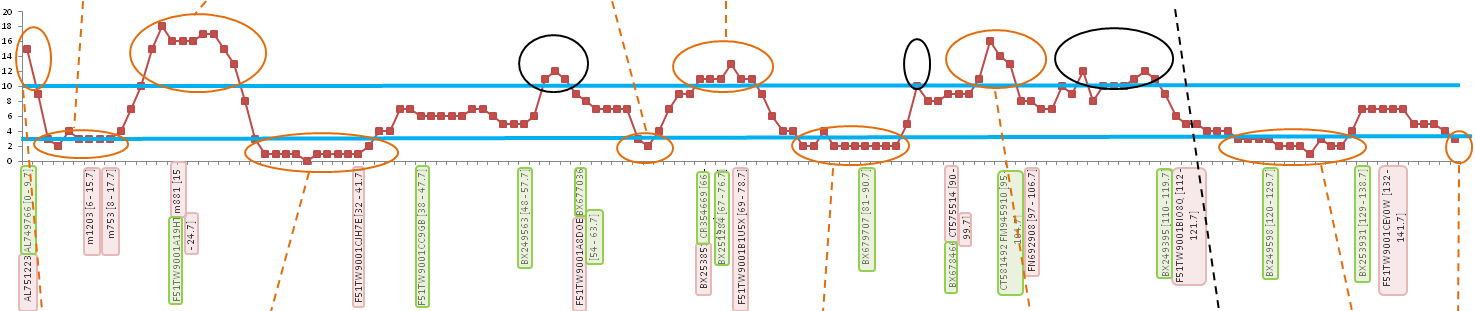


F2


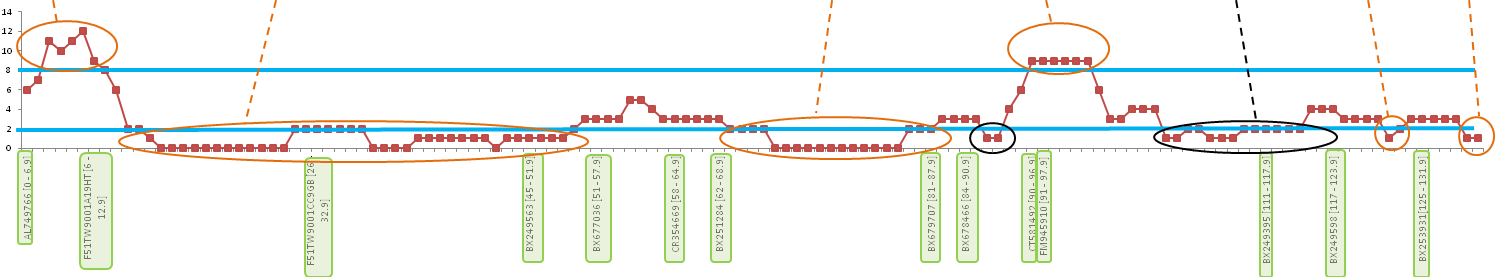


G2M


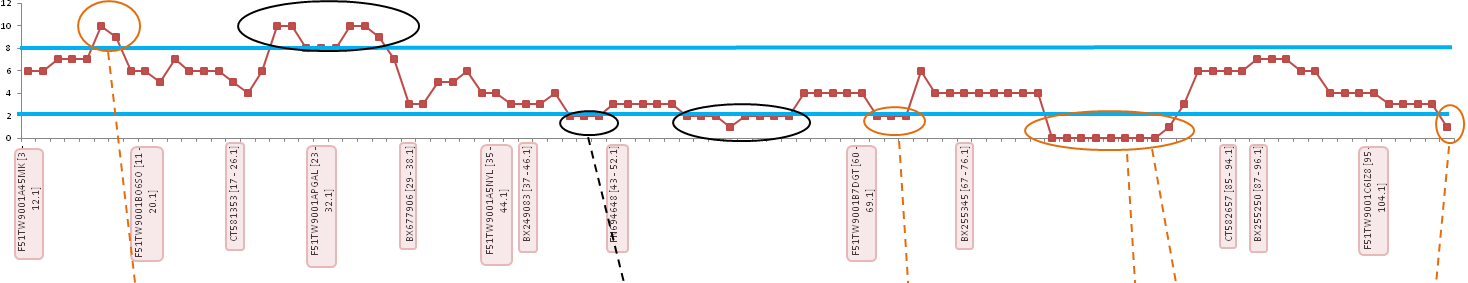
**
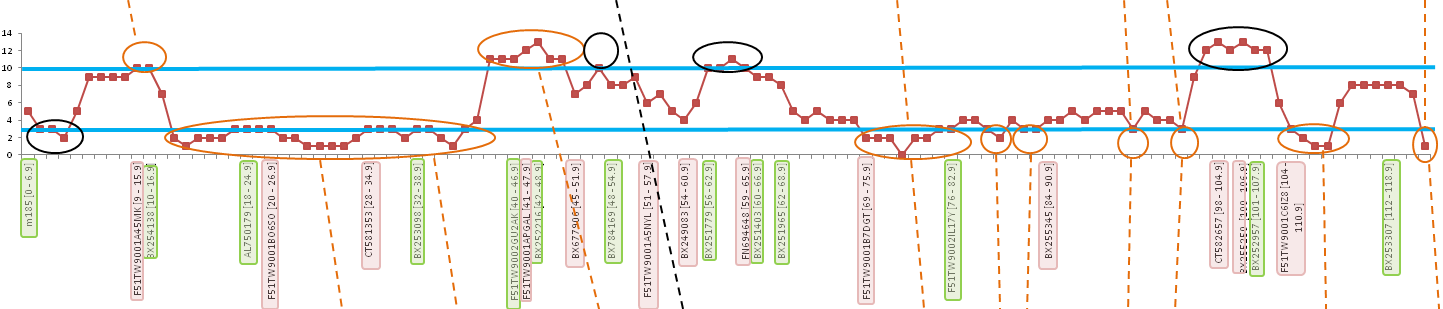
**

G2F

F2

**LG12**


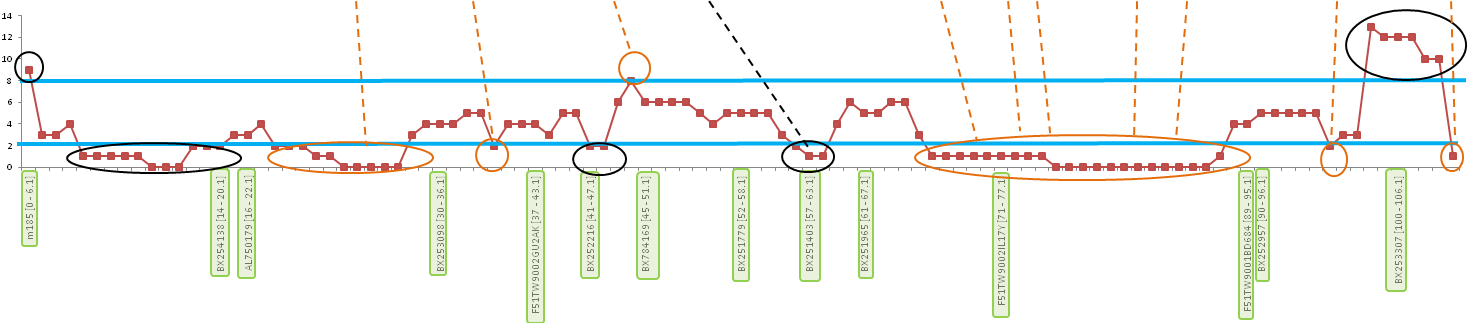


G2M

|  | **Cold spot of recombination** | | | | | | | **Hot spot of recombination** | | | | | | |
| --- | --- | --- | --- | --- | --- | --- | --- | --- | --- | --- | --- | --- | --- | --- |
|  | Unique G2F | Unique G2M | Unique F2 | Common G2F-F2 | Common G2M-F2 | Common G2F-G2M | Common G2F-G2M-F2 | Unique G2F | Unique G2M | Unique F2 | Common G2F-F2 | Common G2M-F2 | Common G2F-G2M | Common G2F-G2M-F2 |
| LG1 | 2 | 0 | 0 | 0 | 2 | 0 | 0 | 2 | 0 | 0 | 1 | 0 | 0 | 3 |
| LG2 | 0 | 0 | 1 | 3 | 0 | 0 | 2 | 1 | 1 | 1 | 0 | 2 | 0 | 3 |
| LG3 | 0 | 0 | 2 | 1 | 1 | 0 | 1 | 1 | 1 | 0 | 3 | 1 | 0 | 3 |
| LG4 | 1 | 2 | 1 | 1 | 2 | 0 | 0 | 2 | 1 | 1 | 3 | 0 | 1 | 1 |
| LG5 | 1 | 0 | 0 | 0 | 2 | 0 | 0 | 0 | 0 | 1 | 0 | 3 | 2 | 3 |
| LG6 | 0 | 1 | 1 | 1 | 0 | 0 | 1 | 3 | 3 | 1 | 0 | 2 | 1 | 1 |
| LG7 | 0 | 1 | 4 | 0 | 0 | 0 | 1 | 3 | 2 | 2 | 1 | 0 | 3 | 0 |
| LG8 | 1 | 0 | 0 | 1 | 1 | 1 | 0 | 0 | 0 | 0 | 1 | 1 | 0 | 2 |
| LG9 | 2 | 0 | 2 | 0 | 2 | 0 | 0 | 0 | 0 | 0 | 3 | 1 | 0 | 3 |
| LG10 | 1 | 0 | 1 | 0 | 2 | 1 | 0 | 1 | 2 | 1 | 0 | 3 | 0 | 5 |
| LG11 | 1 | 0 | 3 | 2 | 2 | 0 | 0 | 0 | 0 | 0 | 2 | 4 | 0 | 0 |
| LG12 | 1 | 2 | 3 | 1 | 1 | 0 | 0 | 0 | 3 | 1 | 0 | 4 | 0 | 4 |

G2F

G2M

10

13

2

7

6

13

5

28

10

14

15

21

18

8

F2
