# Supplementary material for: High-density linkage mapping in a pine tree reveals a genomic region associated with inbreeding depression and provides clues to the extent and distribution of meiotic recombination
Source: BMC Biol. 2013 Apr 18;11:50. doi: 10.1186/1741-7007-11-50 (PMC3660193; doi:10.1186/1741-7007-11-50)
Supplement: Additional file 11 — Distribution of the map distance between two adjacent mapped markers for the three maps (G2F, G2M, F2). x-axis: distance between the markers, y-axis: number of intervals. [file 1741-7007-11-50-S11.doc]

**Additional file 11.** Distribution of the map distance between two adjacent mapped markers for the three maps (G2F, G2M, F2). *x*-axis: distance between the markers, *y*-axis: number of intervals.
