# Supplementary material for: High-density linkage mapping in a pine tree reveals a genomic region associated with inbreeding depression and provides clues to the extent and distribution of meiotic recombination
Source: BMC Biol. 2013 Apr 18;11:50. doi: 10.1186/1741-7007-11-50 (PMC3660193; doi:10.1186/1741-7007-11-50)
Supplement: Additional file 12 — Polymorphism rate (last column) estimated from in silico screening of aligned 454 reads for the three mapped genotypes (10.159.3, G2M map; 9.106.3, G2F map; and H12, F2 map). [file 1741-7007-11-50-S12.doc]

**Additional file 12.** Polymorphism rate (last column) estimated from *in silico* screening of aligned 454 reads for the three mapped genotypes (10.159.3, G2M map; 9.106.3, G2F map; and H12, F2 map).

|  | **Aligned reads^1^** | **Aligned bp** | **Nb of contigs** | **Nb of SNPs^2^ (10x coverage)** | **SNP/bp** |
| --- | --- | --- | --- | --- | --- |
| 10.159.3 | 126,361 | 52,097,318 | 30,150 | 1,672 | 3.21*10^-5^ |
| 9.106.3 | 91,501 | 36,927,456 | 25,574 | 966 | 2.60*10^-5^ |
| H12 | 98,087 | 39,759,775 | 29,628 | 1,106 | 2.78*10^-5^ |

^1^on pine_contigV2, using BWA (V0.6.1-r104)

^2^ detected using a SNP detection pipeline: the alignment files were first process with samtools mpileup (option -Eu) version 0.1.18 (r982:295) to produce a pileup file. This file was used as entry for the samtools vcfutils.pl scripts which called the SNPs. Last, the SNPs were filtered and counted by sam-stats (ea-utils version 1.1.2-r375).
