# Supplementary material for: High-density linkage mapping in a pine tree reveals a genomic region associated with inbreeding depression and provides clues to the extent and distribution of meiotic recombination
Source: BMC Biol. 2013 Apr 18;11:50. doi: 10.1186/1741-7007-11-50 (PMC3660193; doi:10.1186/1741-7007-11-50)
Supplement: Additional file 13 — Pinus pinaster pedigrees used for linkage mapping. [file 1741-7007-11-50-S13.doc]

**Additional file 12.** *Pinus pinaster* pedigrees used for linkage mapping.
