# Supplementary material for: High-density linkage mapping in a pine tree reveals a genomic region associated with inbreeding depression and provides clues to the extent and distribution of meiotic recombination
Source: BMC Biol. 2013 Apr 18;11:50. doi: 10.1186/1741-7007-11-50 (PMC3660193; doi:10.1186/1741-7007-11-50)
Supplement: Additional file 14 — Overview of the EST datasets used to construct PineContig_v2. The accession ID of 454 data is as for the Sequence Read Archive of the NCBI database [82]. [file 1741-7007-11-50-S14.doc]

**Additional file 14.** Overview of the EST datasets used to construct PineContig_v2. The accession ID of 454 data is as for the Sequence Read Archive of the NCBI database (http://www.ncbi.nlm.nih.gov/sra).

| Tissue | Number of genotypes (population) | Library  Short name | Number of clones sequenced | Number of informative reads |  |
| --- | --- | --- | --- | --- | --- |
| **Sanger Method** | | | | |  |
| **Subtractive Suppressive Libraries (SSH)** | | | | |  |
| Mature wood enriched library | 2 (Aquitaine) | 26237 | 1,536 | 1,194 |  |
| Juvenile wood enriched library | 2 (Aquitaine) | 26234 | 1,536 | 1,241 |  |
| Earlywood enriched library | 2 (Corsica) | 24149 | 1,536 | 556 |  |
| Latewood enriched library | 2 (Corsica) | 26230 | 1,536 | 1,073 |  |
| Differentiating xylem enriched library | 2 (Corsica) | 26221 | 1,536 | 1,199 |  |
| **Conventional cDNA libraries** | | | | |  |
| Differentiating xylem | 4 (Corsica) | 12700 | 9,814 | 8,044 |  |
| Buds | 3 2 (Spain) | 19295 | 9,035 | 8,908 |  |
| Roots | 48 (Aquitaine) | 10928 and 10929 | 12,963 | 8,770 |  |
| Needles | 48 (Aquitaine) | 10926 and 10927 | 1,282 | 766 |  |
| Other | sequences recovered from nr database | 26235, 26097 and 12219 | NA | 1,483 |  |
| *Total Sanger* |  |  | *43,132* | *33,234* |  |
| **Pyrosequencing Method (454 Titanium)** | | | | | **SRA** |
| buds sampled on well watered improved seedlings | 9 (Aquitaine) | Pine AH | 192,003 | 70,235 | SRX032960 |
| buds sampled on drought stress improved seedlings | 9 (Aquitaine) | Pine AS | 237,823 | 108,837 | SRX032961 |
| buds sampled on well watered non improved seedlings) | 9 (Aquitaine) | Pine NAH | 190,404 | 49,970 | SRX032962 |
| buds sampled on drought stressed non improved seedlings | 9 (Aquitaine) | Pine NAS | 147,994 | 33,533 | SRX032963 |
| Composite cDNA library: young and old needles, quiescent and swelling buds | 1 (Aquitaine) | 0284-1 | 384,364 | 195,772 | SRX031546 |
| Composite cDNa library: young and old needles, quiescent and swelling buds | 1 (Corsica) | 110-4019-1 | 188,494 | 107,082 | SRX031589 |
| Composite cDNa library: young and old needles, quiescent and swelling buds | 1 (Aquitaine) | 10-159_3 | 200,979 | 125,186 | SRX031587 |
| Composite cDNa library: young and old needles, quiescent and swelling buds | 1 (Morroco) | 112-4-1 | 458,882 | 225828 | SRX031592 |
| Composite cDNa library: young and old needles, quiescent and swelling buds | 1 (Aquitaine) | 9-106-3 | 166,130 | 90,502 | SRX031590 |
| Composite cDNa library: young and old needles, quiescent and swelling buds | 1 (Corsica x Aquitaine hybrid) | H12 | 172,892 | 96,851 | SRX031594 |
| Somatic embryo | (Aquitaine) | Embryome | 990,405 | 878,630 | SRX022618 |
| *total pyrosequencing* |  |  | *3,330,370* | *1,982,426* |  |
|  |  |  |  |  |  |
| ***Total Sanger+Pyrosequencing*** |  |  | ***3,373,502*** | ***2,015,660*** |  |
