# Supplementary material for: High-density linkage mapping in a pine tree reveals a genomic region associated with inbreeding depression and provides clues to the extent and distribution of meiotic recombination
Source: BMC Biol. 2013 Apr 18;11:50. doi: 10.1186/1741-7007-11-50 (PMC3660193; doi:10.1186/1741-7007-11-50)
Supplement: Additional file 15 — PineContig_v2 libraries produced with Aquitaine genotypes of maritime pine. [file 1741-7007-11-50-S15.doc]

**Additional file 15.** PineContig_v2 libraries produced with Aquitaine genotypes of maritime pine.

| **Library ID** | **Read type** | **Number of reads before cleaning** | **Number of reads after cleaning** |
| --- | --- | --- | --- |
| sample_0284_1 | 454 | 195 772 | 166 219 |
| sample_10_159_3 | 454 | 125 186 | 107 840 |
| sample_9_106_3 | 454 | 90 502 | 75 927 |
| pine_AH_run_F7JJN6E0x | 454 | 37 271 | 31 729 |
| pine_AH_run_F7QVD2L0x | 454 | 32 964 | 28 171 |
| pine_AS_run_F7JJN6E0x | 454 | 36 469 | 30 618 |
| pine_AS_run_F7QVD2L0x | 454 | 72 368 | 61 365 |
| pine_NAH_run_F7JJN6E0x | 454 | 28 684 | 24 640 |
| pine_NAH_run_F7QVD2L0x | 454 | 21 286 | 18 397 |
| pine_NAS_run_F7QVD2L0x | 454 | 11 115 | 9 477 |
| pine_NAS_run_F7JJN6E0x | 454 | 22 418 | 19 088 |
| 26235 | Sanger | 1 114 | 1 028 |
| 26234 | Sanger | 1 241 | 1 070 |
| 10929 | Sanger | 4 291 | 3 815 |
| 10928 | Sanger | 4 479 | 4 049 |
| 10927 | Sanger | 518 | 435 |
| 10926 | Sanger | 248 | 221 |
| **17 libraries** | **-** | **685,926** | **584,089** |
