# Supplementary figures and images for: High-density linkage mapping in a pine tree reveals a genomic region associated with inbreeding depression and provides clues to the extent and distribution of meiotic recombination
Source: BMC Biol. 2013 Apr 18;11:50. doi: 10.1186/1741-7007-11-50 (PMC3660193; doi:10.1186/1741-7007-11-50)

**Additional file 16.** Distribution of the 584,089 cleaned reads obtained from Aquitaine genotypes.

**
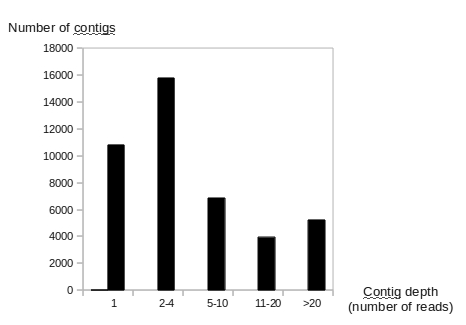
**

Supplement: Additional file 16 — Distribution of the 584,089 cleaned reads obtained from Aquitaine genotypes. [file 1741-7007-11-50-S16.doc]
