# Supplementary material for: High-density linkage mapping in a pine tree reveals a genomic region associated with inbreeding depression and provides clues to the extent and distribution of meiotic recombination
Source: BMC Biol. 2013 Apr 18;11:50. doi: 10.1186/1741-7007-11-50 (PMC3660193; doi:10.1186/1741-7007-11-50)
Supplement: Additional file 17 — Summary of SNPs included in the 12 k bead type Infinium assay. [file 1741-7007-11-50-S17.doc]

**Additional file 17.** Summary of SNPs included in the 12 k bead type Infinium assay.

| Source of SNPs | Number of SNPs* | Number of bead types |
| --- | --- | --- |
| *Set#1: In silico* SNPs from Pine_V2 | 10,171 (3,255) | 11,443 |
| Set#2: *in silico* SNPs in COS markers defined by Chancerel *et al.* (2011) | 185 (66) | 209 |
| Set#3: *in vitro* SNPs from Lepoittevin *et al.* (2010) | 223 (0) | 269 |
| Set#4: *in silico* SNPs in Contig BX248795 | 14 (6) | 17 |
| **Total** | **10,593 (3,327)** | **11,938** |

*1bp Indel are indicated in parenthesis
