# Supplementary material for: High-density linkage mapping in a pine tree reveals a genomic region associated with inbreeding depression and provides clues to the extent and distribution of meiotic recombination
Source: BMC Biol. 2013 Apr 18;11:50. doi: 10.1186/1741-7007-11-50 (PMC3660193; doi:10.1186/1741-7007-11-50)
Supplement: Additional file 19 — Summary of the various types of markers combined with the 12 k-SNP markers for the construction of the G2 (A) and F2 (B) linkage maps. [file 1741-7007-11-50-S19.doc]

**Additional file 19.** Summary of the various types of markers combined with the 12 k-SNP markers for the construction of the G2 (A) and F2 (B) linkage maps.

| A | 1,536-SNP assay (Chancerel et al. 2011^1^) | EST-Ps  (Chagné et al. 2003^2^) | SSRs  (Chagné et al. 2004^3^) |
| --- | --- | --- | --- |
| Number of markers | 299 | 50 | 31 |
| Number of offspring | 89 | 90 | 90 |

| B | 1,536-SNP assay (Chancerel *et al.* 2011) | 384-SNP assay (unpublished) |
| --- | --- | --- |
| Number of markers | 193 | 137 |
| Number of offspring | 88 | 472 |

^1^Chancerel E, Lepoittevin C, Le Provost G, Lin YC, Jaramillo-Correa JP, Eckert AJ, Wegrzyn JL, Zelenika D, Boland A, Frigerio JM, Chaumeil P, Garnier-Géré P, Boury C, Grivet D, Gonzalez-Martinez SC, Rouzé P, van de Peer Y, Neale DB, Cervera MT, Kremer A, Plomion C: **Development and implementation of a highly-multiplexed SNP array for genetic mapping in maritime pine and comparative mapping with loblolly pine**. *BMC Genomics* 2011, **12**:368.

^2^Chagné D, Brown G, Lalanne C, Madur D, Pot D, Neale D, Plomion C: **Comparative genome and QTL mapping between maritime and loblolly pines**. *Mol Breed* 2003, **12**:185-195.

^3^Chagné D, Chaumeil P, Ramboer A, Collada C, Guevara A, Cervera M-T, Vendramin GG, Garcia V, Frigerio JM, Echt C, Richardson T, Plomion C: **Cross species transferability and mapping of genomic and cDNA SSRs in pines**. *Theor Appl Genet* 2004, **109**:1204-1214.
