# Supplementary material for: High-density linkage mapping in a pine tree reveals a genomic region associated with inbreeding depression and provides clues to the extent and distribution of meiotic recombination
Source: BMC Biol. 2013 Apr 18;11:50. doi: 10.1186/1741-7007-11-50 (PMC3660193; doi:10.1186/1741-7007-11-50)
Supplement: Additional file 20 — Bandwidth values obtained from Kernel density analysis for the F2, G2F and G2M linkage maps. [file 1741-7007-11-50-S20.doc]

**Additional file 20.** Bandwidth values obtained from Kernel density analysis for the F2, G2F and G2M linkage maps.

|  | Bandwith in cM  (from Kernel density analysis) | | |
| --- | --- | --- | --- |
|  | F2 | G2F | G2M |
| LG1 | 9.3 | 8.4 | 9.6 |
| LG2 | 11.7 | 10.9 | 8.1 |
| LG3 | 8.9 | 7.2 | 5.9 |
| LG4 | 11.1 | 5 | 13.7 |
| LG5 | 9.9 | 11.9 | 6 |
| LG6 | 8.7 | 8.7 | 7.6 |
| LG7 | 14.6 | 5.4 | 5.5 |
| LG8 | 10.9 | 15.9 | 5 |
| LG9 | 5.8 | 7.3 | 8.5 |
| LG10 | 10.6 | 12.6 | 6.9 |
| LG11 | 6.9 | 17.8 | 6.9 |
| LG12 | 9.7 | 9.1 | 6.1 |
